# Unraveling *Gardnerella vaginalis* Surface Proteins Using Cell Shaving Proteomics

**DOI:** 10.3389/fmicb.2018.00975

**Published:** 2018-05-15

**Authors:** Elvira Marín, Annelies Haesaert, Laura Padilla, Jaume Adán, María L. Hernáez, Lucía Monteoliva, Concha Gil

**Affiliations:** ^1^Departamento de Microbiología y Parasitología, Facultad de Farmacia, Universidad Complutense de Madrid, Madrid, Spain; ^2^Health and Biomed Division, LEITAT Technological Center, Barcelona, Spain; ^3^Unidad de Proteómica, Facultad de Farmacia, Universidad Complutense de Madrid, Madrid, Spain; ^4^Instituto Ramón y Cajal de Investigación Sanitaria (IRYCIS), Madrid, Spain

**Keywords:** *Gardnerella vaginalis*, cell shaving, surface proteins, ABC-transporters, trypsin digestion, GroEL, Cna

## Abstract

*Gardnerella vaginalis* is one of the main etiologic agents of bacterial vaginosis (BV). This infection is responsible for a wide range of public health costs and is associated with several adverse outcomes during pregnancy. Improving our understanding of *G. vaginalis* protein cell surface will assist in BV diagnosis. This study represents the first proteomic approach that has analyzed the exposed proteins on *G. vaginalis* cell surface using a shaving approach. The 261 *G. vaginalis* proteins identified using this approach were analyzed with bioinformatic tools to detect characteristic motifs from surface-exposed proteins, such as signal peptides (36 proteins), lipobox domains (17 proteins), LPXTG motifs (5 proteins) and transmembrane alpha-helices (66 proteins). One third of the identified proteins were found to have at least one typical motif of surface-exposed proteins. Furthermore, the subcellular location was examined using two predictors (PSORT and Gpos-mPLoc). These bioinformatic tools classified 17% of the identified proteins as surface-associated proteins. Interestingly, we identified 13 members of the ATP-binding cassette (ABC) superfamily, which were mainly involved in the translocation of various substrates across membranes. To validate the location of the *G. vaginalis* surface-exposed proteins, an immunofluorescence assay with antibodies against *Escherichia coli* GroEL was performed to reveal the extracellular location of the moonlighting GroEL. In addition, monoclonal antibodies (mAb) against *G. vaginalis* Cna protein were produced and used to validate the location of Cna on the surface of the *G. vaginalis*. These high affinity anti-Cna mAb represent a useful tool for the study of this pathogenic microorganism and the BV.

## Introduction

Bacterial vaginosis (BV) is the most common vaginal disorder among women of reproductive age (Koumans et al., 2007). Its prevalence is high among vulvovaginal infections, although its exact percentage depends on the study group (Sobel, 2000; Sabour et al., 2018). It is responsible for various symptoms including vaginal discharge, which is typically homogenously milky or gray-colored and malodorous. BV causes a rise in the production of amines that increase vaginal pH to over 4.5 and is characterized by the presence of epithelial “clue cells,” which are indicative of the *Gardnerella vaginalis* infection; however, it is usually asymptomatic and does not feature an inflammatory reaction (Catlin, 1992). In healthy vaginal epithelium, commensal *Lactobacillus* species produce hydrogen peroxide and lactic acid, resulting in an acidic pH and inhibiting the proliferation of other bacteria (Machado et al., 2013). BV is characterized by an imbalance in this vaginal microbiota from the commensal lactobacilli to obligate anaerobes; for this reason, BV has a polymicrobial etiology (Kenyon and Osbak, 2014). BV has been linked to serious public health consequences, including postoperative infections (Kavoussi et al., 2006) and the acquisition and transmission of the human immunodeficiency virus (HIV) (Atashili et al., 2008; Masson et al., 2014). It also increases susceptibility to acquire the human papillomavirus (HPV) (Peres et al., 2015), the herpes simplex virus type 2 (HSV-2) (Kaul et al., 2007) and other pathogens that infect the lower genital tract (St John et al., 2007). Furthermore, BV enhances the risk of preterm birth and is associated with several adverse outcomes in pregnancy (Bretelle et al., 2015; Giakoumelou et al., 2015). Due to the lack of specific symptoms of BV (Kenyon and Osbak, 2014), highly accurate molecular assays are needed. With this objective, methods as quantitative real-time PCR (qPCR) have been used in order to obtain molecular cutoff values for BV diagnosis (Menard et al., 2008) and also a confident laboratory tool to assist in the asymptomatic BV (Hilbert et al., 2016). But these techniques require trained specialist and equipment, for all these reasons, developing a test based on an immunoassay could be an alternative for the diagnosis of BV at any point of care, even in developing countries.

*Gardnerella vaginalis* had been found in 87% of women without a BV diagnosis and in almost all BV-positive samples (Janulaitiene et al., 2017). *G. vaginalis* appears in association with other anaerobes in BV, such as *Atopobium vaginae, Mobiluncus mulieris, Prevotella bivia, Fusobacterium nucleatum*, and *Peptoniphilus* species, highlighting the polymicrobial etiology of this pathology (Machado and Cerca, 2015; Jung et al., 2017). While the specific role of *G. vaginalis* in BV remains controversial, two outcomes are generally recognized: the formation of a biofilm on the vaginal epithelium and the presence of *G. vaginalis* as the predominant species of bacteria in this pathology (Machado and Cerca, 2015). *G. vaginalis* is a Gram-positive, rod-shaped bacterium with a cell wall composed of a thin peptidoglycan (PG) layer (Catlin, 1992). It is characterized by Gram-variable staining and a high GC-content. The taxonomic classification of *G. vaginalis* has proved controversial as it was initially named *Haemophilus vaginalis* (Gardner and Dukes, 1955) then renamed *Corynebacterium vaginale* (Zinnemann and Turner, 1963). Finally, a new genus with only one species was categorized as *G. vaginalis*.

The cell wall of the microorganism is the first point of contact with the environment and is associated with the initial adherence of the bacteria to the vaginal epithelium. The cell wall contains cell surface proteins, which are involved in the signaling, transport and up-take of nutrients, in addition to playing an important role in pathogenesis due to inter- and intracellular interactions (Navarre and Schneewind, 1999). Gram-positive bacteria have specific mechanisms by which proteins can move from the cytoplasm into or over the membrane, such as twin-arginine protein translocation (Tat) and general secretory pathways (Sec; SecYEG translocon) (Schneewind and Missiakas, 2012; Goosens et al., 2014). Proteins are directed toward the secretory systems by N-signal peptides, followed by their translocation across the membrane where they are cleaved by peptidase I (Schneewind and Missiakas, 2014). Proteins can be retained in the cell wall through covalent attachment to the PG, which is mediated by the C-terminal sorting signal LPXTG motif, a mechanism that is catalyzed by sortase enzymes (Schneewind and Missiakas, 2014). In general, pre-pro-lipoproteins gain access to the membrane via the Sec pathway or the Tat pathway (Zuckert, 2014). Peptidase II often cleaves immediately before the conserved cysteine residue of the lipobox motif (Dalbey et al., 2012; Schneewind and Missiakas, 2014). This cysteine residue is also a target for the lipid modification of lipoproteins to retain these proteins in the plasma membrane-cell wall interface (Kovacs-Simon et al., 2011; Krishnappa et al., 2013).

The identification of surface proteins, or surfome, by shaving involves the application of a protease treatment to whole cells to generate peptides followed by a LC-MS/MS analysis. This has been used in eukaryotic (Hernaez et al., 2010; Vialas et al., 2012; Gil-Bona et al., 2015; Marin et al., 2015) and prokaryotic microorganisms mainly in Gram-positive bacteria (Olaya-Abril et al., 2014). The shaving procedure bypasses several problems associated with surface protein analyses, such as low abundance when compared with cytoplasmic proteins and low solubility, both of which make protein extraction more difficult. Moreover, it avoids subcellular pre-fractionation. However, cell lysis must be controlled to avoid cytoplasmic protein contamination. Overall, shaving is a fast and reliable way to identify cell wall proteins, integral membrane proteins and associated surface proteins.

In this study, we aimed to investigate the surface-associated proteins of *G. vaginalis* to identify diagnostic markers or therapeutic targets of BV. We carried out a gel-free proteomic approach by direct trypsin digestion (shaving) over whole *G. vaginalis* bacteria. To the best of our knowledge this is the first time this approach has been used for this purpose. We identified 261 *G. vaginalis* proteins, one third of which predicted motifs typical of surface-associated proteins, including signal peptide (SP), lipobox, LPXTG motif and transmembrane alpha-helix domains (TMDs).

## Materials and Methods

### Bacterial Strains and Growth Conditions

The *G. vaginalis* strain used in this study was ATCC14018 (JCM 11026T), it was isolated from vaginal samples (Oshima et al., 2015). Bacteria cells were exclusively cultured in Brain Heart Infusion (BHI) at 37°C and 5% CO_2_.

The strain of *Escherichia coli* used for cloning was DH10B T1R, and for gene expression it was BL21 DE3. Both strains were provided as gifts from the Dr. Luis A. Fernández Laboratory. The *E. coli* strains used in the experiments were grown in Luria Bertani (LB) medium at 37°C and 200 rpm. The antibiotic used was kanamycin (Km) at 50 μg/ml.

### Surface Shaving

Bacteria cells from an early exponential growth phase culture (100 ml; OD_600_ ∼ 0.2) were harvested by centrifugation and washed three times with sterile-filtered phosphate-buffered saline (PBS). Cells were re-suspended in 1 ml PBS containing 30% sucrose and 3 μg of recombinant sequencing grade trypsin (ROCHE) was added. Incubation was done during 30 min at 37°C and 300 rpm. After the trypsin treatment, samples were centrifuged at 4000 rpm for 10 min and the supernatant (containing protein and peptides) was filtered with a filter unit of 0.22 μm. The flow-through was re-digested overnight with 2 μg of fresh recombinant trypsin in the same conditions described above. A volume of 100 μl of trifluoroacetic acid (TFA) 0.1% (v/v) was added to stop the proteolytic reaction. Subsequently, originated peptides were cleaned up with a Poros R2 resin (AB Sciex, Framingham, MA, United States). Peptides were eluted with 80% acetonitrile in 0.1% TFA, dried in a Speed-Vac and re-suspended in 0.1% formic acid. The samples were stored at -20°C prior to nano-LC-MS/MS analysis. Cell pellets were collected before and after the first trypsin incubation, and the bacterial cell viability was evaluated by plating on Agar *Gardnerella* (Biomerieux) and colony-forming units (CFU) were counted. The experiment was performed in triplicate.

### LTQ-Orbitrap Velos Analysis and Protein Identification

Peptides were analyzed using RP-LC/MS in an Easy-nLC II system coupled to an ion trap LTQ-Orbitrap-Velos-Pro mass spectrometer (Thermo Scientific). The peptides were concentrated (on-line) by reverse phase chromatography using a 0.1 mm × 20 mm C18 RP pre-column (Thermo Scientific), and then separated using a 0.075 mm × 250 mm C18 RP column (Thermo Scientific) operating at 0.3 μl/min. Peptides were eluted using a 110-min gradient from 0 to 40% solvent B (solvent A: 0.1% formic acid in water; solvent B: 0.1% formic acid, 80% acetonitrile in water). ESI ionization was achieve using a Nano-bore emitters Stainless Steel ID 30 μm (Proxeon) interface. Peptides were detected in survey scans from 400 to 1600 amu (1 μscan), followed by fragmentation of the 15 most intense ions by Collision Induced Dissociation using an isolation width of 2 (in mass-to-charge ratio units), normalized collision energy of 35%, and dynamic exclusion applied in 30 s intervals.

Protein identification from mass spectra raw files was carried out using Proteome Discoverer software version 1.4.1.14 (Thermo Scientific) on a licensed version of the search engine MASCOT 2.3.0. Data Base Searchers were performed to identify peptides and proteins of *G. vaginalis* ATCC14018/JCM 11026 strain (1,277sequences), data available on NCBI^1^. The following search parameters were used: tryptic cleavage after arginine and lysine, up to two missed cleavage sites allowed, tolerances of 20 ppm for precursor ions and 0.8 Da for MS/MS fragment ions, optional Methionine oxidation and fixed carbamido-methylation of cysteine.

A search of the decoy database (adopting the integrated decoy approach) was used to calculate the FDR. The MASCOT percolator filter was applied to the MASCOT results. The acceptances criteria for protein identification were: a FDR < 1% and at least one peptide identified with high confidence (CI > 95%). The proteins identified in two out of three replicates with at least two peptides in one were used in further analysis.

The mass spectrometry proteomics data have been deposited to the ProteomeXchange Consortium (Vizcaino et al., 2014) via the PRIDE partner repository^2^ with the dataset identifiers PXD003192 and 10.6019/PXD003192.

### Bioinformatic Analysis

The signal peptide (SP) of the Sec secretion pathway was predicted using SignalP4.1^3^ (Petersen et al., 2011). For Tat secretion pathway, the SP was predicted using TatP 1.0^4^ and the lipo-SP was predicted using PRED-LIPO^5^. Transmembrane alpha-helix domains (TMD) were predicted using TMHMM^6^. LPXTG domain and lipobox identification were achieved through the PATTINPROT program^7^. The pattern used to identify the lipobox was [DERK](6)-[LIVMFWSTAG](2)-[LIVMFYSTAGCQ]-[AGS]-C, which was taken from (Sutcliffe and Harrington, 2002). Using the LocateP database of *G. vaginalis* ATCC14019 strain, we were able to identify additional proteins with LPXTG domain^8^ by homology between protein sequences. Always identity between protein sequences was between 95 and 100%. Subcellular localization probabilities were determined using the PSORT server^9^, which also predicted SP and TMDs. Additionally, subcellular location was achieved using the Gpos-mPLoc server, which is specific for Gram-positive bacterial proteins^10^. We created topological representations of proteins using the PROTTER program, which identified SP and TMDs^11^. The Pfam server^12^ allows the analysis of the protein primary sequence to find a Pfam family classification. Blastp was used to identify homology with proteins in other microorganisms^13^. Finally, to represent the consensus sequence of the ABC transporters WebLogo tools were used^14^.

### Plasmid, DNA Constructs and Oligonucleotides

DNA manipulation, ligation, transformation and plasmid preparation were performed following standard techniques. All DNA constructs were sequenced in the Center of Genomic and Proteomics of Universidad Complutense of Madrid. PCR reactions were performed using the Expand High Fidelity PCR system (ROCHE). Plasmid selected for gene expression was pET-29a (+) (Novagen) with a 6xHis-tag at C-terminal and a Km resistance cassette as marker. Sigma Genosys was used to synthesize the oligonucleotides NdeI-up 5′-GGAATTCCATATGCAGTCGAGCAATGATAATGCTT-3′ and XhoI-down 5′- CTGGCTCGAGGTTAGCATCAAACCACACGC-3′ (restriction enzymes were underlined). DNA fragment corresponding to amino acids 35 to 540 of Cna (indicated in Supplementary Data) was subject to PCR amplification using the genomic DNA of *G. vaginalis* ATCC 14018 with these oligonucleotides, digested with NdeI and XhoI, and ligated into the same sites of the vector backbone pET29a. The M protein repeat protein was cloned following the same procedure, for the amino acid 51 to the end. The oligonucleotides designed were NdeI-up 5′-GGAATTCCATATGGCCGACGCGACTACAA-3′ and XhoI-down 5′- CTGGCTCGAGCTTGCGACGGATTCG-3′.

### Protein Purification

The purification of the His-tagged C-terminal Cna protein was performed as described below. The *E. coli* BL21 DE3 cells carrying plasmid pET29a-Cna were grown in 1 liter culture of LB broth at 37°C with agitation (250 rpm). When the OD_600_ reached around 0.5, they were induced with 0.1 mM IPTG for 4 h. Cells were subsequently harvested by centrifugation (4,000 × *g* for 10 min) and each gram of cell-pellet was resuspended in 5 ml of purification buffer [buffer P: 50 mM NaH_2_PO_4_, 200 mM NaCl at pH 8 containing a cocktail of protease inhibitors (Complete EDTA-free; Roche)]. Lysozyme was added at 1 mg/ml and incubated for 30 min at 4°C. The following steps were carried out at 4°C. The suspension of cells was sonicated with ten pulses of 20 s (Vibra-cell; Sonics & Materials), followed by centrifugation (4,000 × *g* for 10 min) to discard non-lysed cells. The supernatant was centrifuged once more (22,000 × *g* for 30 min). The pellet was resuspended in 10 ml of buffer P containing 1.5% (wt/vol) *N*-lauroylsarcosine sodium salt (Sarkosyl; Sigma) and a cocktail of protease inhibitors, incubated for 1 h in a wheel and sonicated briefly to favor solubilization. After incubation, the mixture was centrifuged again (22,000 × *g* for 30 min). An 8-ml aliquot of a nickel-containing agarose resin (50%, vol/vol) (Ni-NTA) equilibrated in buffer P was then added. The resulting suspension was incubated overnight with slow agitation on a gyratory wheel to favor binding of the Cna-His-tagged protein. The next day, this mixture was passed through a chromatography column containing an additional 2 ml of Ni-NTA resin. This column was washed with buffer P containing imidazole, first with 10 mM and a second time with 50 mM. The Cna-His-tagged protein was eluted in 1-ml fractions with the same buffer containing 150 mM of imidazole. Aliquots with a higher amount of protein were concentrated with a centrifugal filter unit cut-off of 50 kDa (Amicon; Millipore) and dialyzed against water with a dialysis cassette cut-off of 10 kDa (Slide-A-Lyzer; Thermo Scientific).

### Custom Mouse Monoclonal Antibody Production

Monoclonal antibody fusion, enzyme-linked immunosorbent assays (ELISA) screening and sub-cloning were performed using standard technologies (Kohler and Milstein, 1975). The maintenance, expansion and scaling up of the cell cultures were carried out in a humidified atmosphere (94% air and 6% CO_2_) at 37°C. Female BALB/cAnNHsd mice (Harlan) were immunized with a recombinant Cna fusion protein according to the following protocol. Seventy-five micrograms of Cna protein diluted in PBS was used as an emulsion with a Complete Freund’s adjuvant (Sigma) for the initial subcutaneous immunization. Subsequent immunizations were given at days 14 and 35 with an Incomplete Freund’s adjuvant. At day 50, a final boost of 40 μg of Cna protein diluted in PBS was given to the mouse via intraperitoneal injection using the highest titrated serum. Fusion was done four days after the last injection. Clones were derived from the fusion of myeloma cells with spleen cells from the selected mouse at a ratio of 1/10, using PEG-1500 (Roche Diagnostics) as a fusion inducer. Then, cells were plated in 96 microwell dishes in a medium containing HAT (Invitrogen) for hybrid selection. Hybridoma supernatants were screened using ELISA for reactivity against recombinant Cna coated at 1 μg/ml. Ninety-five positives clones were re-screened using ELISA for their ability to recognize the native antigen present on the surface of *G. vaginalis*, which was achieved by coating 10^8^ cells/ml and comparing these with the un-specific signals of *E. coli* cells (data not shown). Finally, by limiting dilution seven selected clones with highly antigen-specific reactivity were subcloned to obtain hybridoma secretory cell lines. For subsequent experiments, the purified monoclonal antibody from each selected hybridoma cell line was obtained. To this end, cells were cultured in serum free conditions. After filtration, supernatants were purified on protein A columns (MabSelect Sure^TM^ LX; 25 ml; Amersham) using an ÄKTA purifier FPLC system. Fractions were analyzed by SDS-PAGE. The elution buffer was exchanged to PBS and the antibody was concentrated with Amicon^®^ Ultra-15 centrifugal filter devices with low-binding Ultracel^®^ membranes (30000 NMWL; Millipore). The final purified antibodies were quantified at 280 nm.

### Enzyme-Linked Immunosorbent Assays (ELISA)

A volume of 100 μl of intact bacterial cells or total extracts were absorbed into the ELISA plates (Maxisorb; Nunc) at an OD_600_ of 3.0 and 2 μg/μl, in PBS for 2 h. Next, plates were blocked for 1.5 h with PBS containing 3% (w/v) of skimmed milk. Anti-GroEL POD conjugate (Sigma-Aldrich) was added at 1:5000 dilution to the same buffer and incubated for an additional hour. Anti-Cna mAb mouse custom antibodies were used at 10 μg/ml in the same buffer for 1 h. The plates were then washed five times with PBS, and the presence of bound antibodies were developed using *O*-phenylenediamine (OPD; Sigma), and absorbance was read at 490 nm. The ELISA values reported were from two independent experiments performed in quadruplicates. The Excel Software was used to create the Graphs of the mean and standard deviation values. Total extracts were obtained from the same culture of intact cells resuspended on PBS and briefly sonicated through three pulses of 20 s (Vibra-cell; Sonics & Materials). All incubations were at room temperature.

The statistical significance of the differences in absorbance measures was evaluated using the Student *t*-test (^∗^*p* < 0.05, ^∗∗^*p* < 0.01).

### Confocal Fluorescence Microscopy

Bacterial cultures were centrifuged and resuspended at an OD_600_ of 3.0, and were then incubated for 2 h on glass coverslips pre-coated with poly-L-lysine (1 mg/ml). Cells were fixed with formaldehyde 4% (w/v) (in PBS) for 15 min at room temperature (RT). Glass slides were washed twice with PBS. Then, slides were blocked for 1 h at RT with buffer B [PBS with bovine serum albumin (BSA) at 1 mg/ml]. The slides were washed twice again with PBS and then incubated for 1.5 h at RT in the same buffer with an anti-GroEL (Rabbit; Sigma-Aldrich) at 1:2000 dilution, an anti-Cna mAb at 1:1000 dilution (custom mouse antibodies, number 41) or in buffer only, as indicated on the figures. The slides were washed three times with PBS and further incubation for 1 h with an anti-rabbit IgG or anti-mouse IgG, both conjugated with Alexa-488 diluted at 1:500 in buffer B. Nuclei were stained with DAPI dye (5 μg/ml; 5 min at RT). Mounting medium Fluoromount-G (SouthernBiotech) was added to the preparations. The epifluorescence of the cells was then examined and images were collected using an Olympus FV1200 microscope.

## Results

### Optimization of the Shaving Approach for the Identification of *G. vaginalis* Surface-Associated Proteins

This study describes a proteomic approach to investigate the surface protein composition of *G. vaginalis*, a poorly studied microorganism. *G. vaginalis* is a small, rod-shaped bacterium with a thin PG layer surrounding the plasma membrane, which was considered during the shaving procedure. Our methodology was based on a previous study, which used the shaving approach for *Streptococcus pneumoniae*, a microorganism that is highly susceptible to autolysis (Olaya-Abril et al., 2012). We firstly optimized the shaving process for use with *G. vaginalis* to avoid cell lysis during trypsin treatment. *G. vaginalis* cells were collected at the exponential growth phase, when the rate of cell death is lowest than in any other growth phase, to reduce cytoplasmic protein contamination. The trypsin digestion of *G. vaginalis* cells was initially performed in PBS, but cell lysis was observed. Therefore, we added 30% sucrose to the PBS and tested different amounts of trypsin per sample (1, 2, 3, 5, or 10 μg). To determine the cell integrity of *G. vaginalis*, plate counting was performed to the number of colony-forming units (CFUs) before and after trypsin treatment. We found that 5 and 10 μg of trypsin induced cell lysis, but the number of CFUs in other trypsin amounts (1, 2, and 3 μg trypsin) were comparable. Finally, 3 μg of trypsin was chosen for the first trypsin digestion, and 2 μg of trypsin was used for re-digestion of the supernatant obtained. This treatment rendered good protein digestion for peptide identification using LC-MS/MS analysis.

### Protein Identification and Subcellular Location of *G. vaginalis* Proteins

The cell-surface trypsin shaving and LC-MS/MS analysis performed on three biological replicates, enabling the identification of 261 *G. vaginalis* proteins. These proteins were identified in at least two replicates with at least two peptides in one of these (Supplementary Table S1). Most of the proteins (84.3%) were identified in all replicates. Twenty-five proteins were identified with an average of greater than 10 peptides, and almost half of these were classified as plasma membrane proteins using the PSORT server (**Table 1**).

**Table 1:** *Gardnerella vaginalis* proteins identified with more than 10 peptides and their subcellular location.

Protein_ID^a^	Gene name^a^	Description^a^	Peptide average^b^	Unique peptides^c^	Location by PSORT^d^
**BAQ32758**		**M protein repeat protein^e^**	**53**	**67**	**Plasmatic membrane**
BAQ33431		Hypothetical protein	33	47	Plasmatic membrane
BAQ33644		Hypothetical protein	26	36	Plasmatic membrane
BAQ33076		Xylulose-5-phosphate/fructose-6-phosphate phosphoketolase	23	29	Cytoplasm
BAQ32771		Conserved hypothetical protein	22	27	Plasmatic membrane
BAQ32803		Dipeptide/oligopeptide ABC transporter substrate binding component	21	23	Plasmatic membrane (lipoprotein)
BAQ33274		Transketolase	17	22	Cytoplasm
BAQ33156	*clp-ATP*	ATP-dependent Clp protease ATP-Binding subunit	17	24	Cytoplasm
BAQ32981	*ef-G*	Elongation factor G	16	20	Cytoplasm
BAQ33548	*alaS*	Alanyl-tRNA synthase	15	22	Plasmatic membrane
BAQ33427		Putative cell surface protein	15	21	Plasmatic membrane
BAQ33074	*ackA*	Acetate kinase	14	17	Cytoplasm
BAQ33315		Conserved hypothetical protein	13	21	Cytoplasm
BAQ33818	*atpD*	ATP synthase beta subunit	13	16	Cytoplasm
**BAQ32792**		**Cna protein B-type domain^e^**	**13**	**22**	**Plasmatic membrane**
BAQ33052		Conserved hypothetical protein	12	16	Cytoplasm
BAQ33322		Dehydrogenase	12	13	Cytoplasm
BAQ32818		Putative ABC transporter substrate binding component	12	16	Plasmatic membrane (lipoprotein)
BAQ33450	*pyK*	Pyruvate kinase	12	15	Cytoplasm
BAQ32849		Conserved hypothetical protein	11	16	Cytoplasm
BAQ33816		Hypothetical protein	11	11	Plasmatic membrane
BAQ33428	*rplY*	50S ribosomal protein L25	11	13	Cytoplasm
BAQ33018		Conserved hypothetical protein	10	13	Cytoplasm
BAQ33606		Conserved hypothetical protein	10	13	Plasmatic membrane
BAQ33723	*rpmC*	50S ribosomal protein L29	10	12	Cytoplasm

^a^*Protein_ID, gene name and description from (http://www.ncbi.nlm.nih.gov/Taxonomy/Browser/wwwtax.cgi?id=585528) (Oshima et al., 2015), listed in order of peptide average. ^*b*^Peptide average of triplicate biological replicates. ^*c*^Total number of unique peptides identified in all biological triplicates. ^*d*^Subcellular location was analyzed with PSORT server (http://psort.hgc.jp/form.html). ^*e*^Proteins selected for antibodies production are indicated in bold*.

**FIGURE 1:**
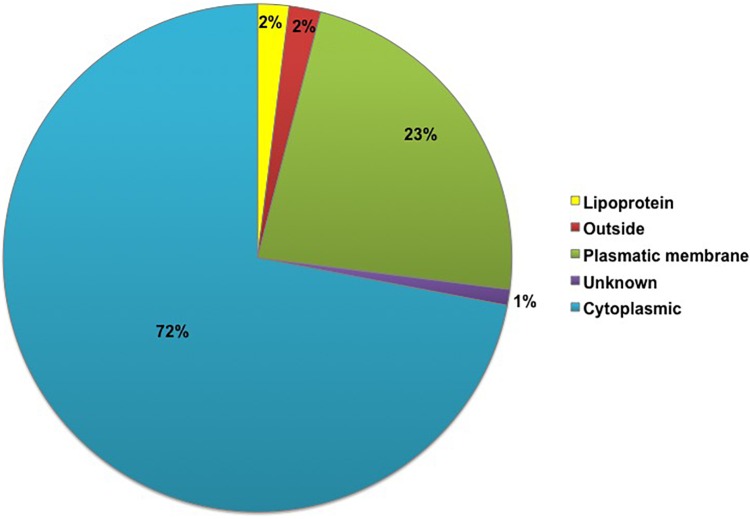
Percentage representation of the subcellular classification of proteins identified in *Gardnerella vaginalis* using a shaving proteomic approach, as determined using the PSORT server. In total, 261 *G. vaginalis* proteins were identified by shaving and were classified by the PSORT server in the following categories: lipoprotein, outside, plasmatic membrane, unclassified and cytoplasm.

The subcellular locations of all 261 proteins identified in the *G. vaginalis* surfome were analyzed *in silico* using the PSORT and Gpos-mPLoc servers in parallel (Supplementary Table S2). Initially, the proteins were categorized into five groups using PSORT: outside, lipoprotein, plasmatic membrane, cytoplasmic and unknown. Among the proteins categorized as cytoplasmic, eight where labeled as ambiguous because other bioinformatic tools detected motifs typical of surface-exposed proteins, as shown in Supplementary Table S1. The percentage of identified proteins in each group is shown in **Figure 1**. After the proteins located in the cytoplasm, the largest number of proteins was found to be in the plasma membrane, with 23% of *G. vaginalis* proteins located there. Three proteins were classified as unknown by PSORT due to their low scores, which did not allow classification into any subcellular location (BAQ32908, BAQ33209, and BAQ33277). The double analysis, by PSORT and Gpos-mPLoc, separated the identified proteins into three main groups according to the prediction of subcellular location: (i) “Inside,” (ii) “Both,” and (iii) “Surface-associated” (Supplementary Table S2). Among the proteins identified, 43 (17%) were predicted to have an extra-cytoplasmic location by the two servers. Alternatively, 86 proteins (33%) were predicted to be classified as both, as one server predicted they were cytoplasmic and the other predicted they were extra-cytoplasmic.

Among the proteins classified as “inside” there are several described in other Gram-positive microorganisms with dual locations (cytoplasm and bacterial surface) or as moonlighting proteins (indicated with a “^∗^” in Supplementary Table S1). These include: enolase (Eno), glyceraldehyde-3-phosphate dehydrogenase (Gap) (Henderson and Martin, 2011; Wang et al., 2014), phosphoglycerate mutase (GpmA), inosine 5′-monophosphate dehydrogenase (IMPDH), pyruvate kinase (PyK), DnaK, GroEL, the elongation factors Tu (EF-Tu) and G (EF-G) and the protein translocase subunit A (SecA).

### Comprehensive *in Silico* Analysis of Protein Motifs Typical of Surface-Exposed Proteins

**FIGURE 2:**
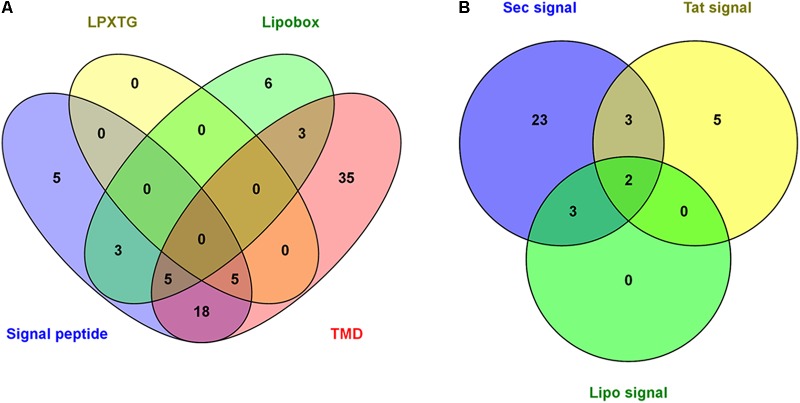
Representation of *G. vaginalis* identified proteins for which surface-exposed domains were found. **(A)** The number of proteins with motifs characteristic of surface-exposed proteins including LPXTG, lipobox, signal peptide (SP) and transmembrane alpha-helix domain (TMD). **(B)** SP prediction for the different secretion system of Gram-positive bacteria. SPs were identified using SignalP 4.1, the PSORT server, the TatP server and the PRED-LIPO server. The lipobox of lipid-anchored proteins and LPXTG motif of cell wall proteins were identified using the PATTINPROT program. TMDs were identified using the TMHMM, PROTTER and PSORT servers. There were 80 unique proteins identified with surface-exposed domains in the *G. vaginalis* surfome.

An exhaustive analysis of the identified proteins was performed using bioinformatic tools to detect the characteristic motifs of surface-exposed proteins, such as the SP, lipobox domain, LPXTG PG-anchoring motif and TMD (Supplementary Table S1). These motifs were detected in a total of 80 proteins, accounting for 31% of the proteins identified. The number of proteins predicted to contain each motif is shown in **Figure 2A**. A SP was identified in 36 *G. vaginalis* proteins using different bioinformatics tools, and these were associated with the following secretion pathways: 31 with the Sec secretion pathway, 10 with the Tat secretion pathway and 5 included lipo-SP motifs (Supplementary Table S3 and **Figure 2B**). For some proteins, the SP was predicted for more than one secretion pathway simultaneously. The five proteins containing the LPXTG motif identified in our study are annotated in the databases as follows: one as a hypothetical protein, two as putative cell surface proteins, one as a conserved hypothetical protein and one as a cell wall associated fibronectin-binding protein (Supplementary Table S1).

**FIGURE 3:**
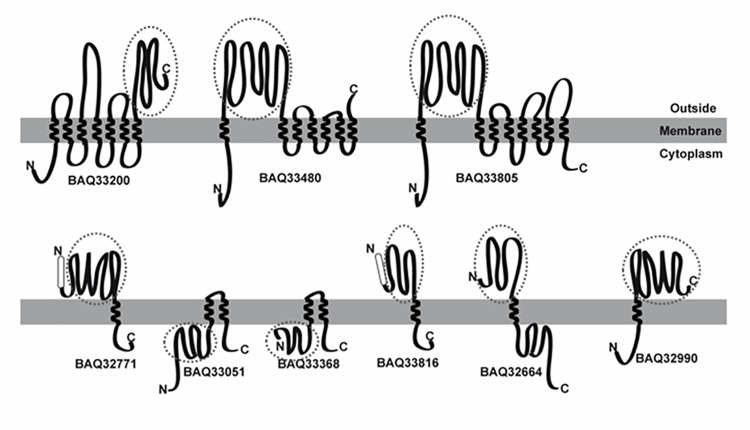
Schematic representation of membrane proteins using the PROTTER server to show-up types of protein architectures. The **(upper)** panel represents membrane proteins with more than two predicted transmembrane alpha-helix domains (TMDs). The **(lower)** panel represents proteins with different topologies, with and without a signal peptide (SP) or with either the N-terminal or C-terminal exposed on the outer membrane leaflet. The protein_ID is shown under each scheme. The SP is represented by a white rectangle, and the TMD as a transmembrane helix. For each schematic protein representation, the N-terminal is depicted on the left and the C-terminal on the right. The identified peptides were matched with the primary sequence, and a circle indicates the matched region.

The presence of TMDs, distinctive of integral membrane proteins, was detected using the PSORT, PROTTER, and TMHMM servers. The number of TMDs predicted using each bioinformatic tool is summarized in Supplementary Table S3. TMDs were detected in 66 *G. vaginalis* proteins, 56 of which had 1 TMD, 7 of which had 2 TMDs and 3 of which had more than 6 TMDs. A schematic of the secondary prediction of poly-transmembrane proteins (more than two TMDs) and examples of proteins with different topologies according to the PROTTER server are shown in **Figure 3**.

A more comprehensive analysis of the sequence of the 43 proteins with surface-exposed motifs mapping the MS identified peptides was done (Supplementary Data). Overlapping was detected between the identified peptides and the surface-exposed region, excluding BAQ33051 and BAQ33368 where the peptides correspond to a cytoplasmic region (**Figure 3**).

### Analysis of Relevant Groups of Proteins

**FIGURE 4:**
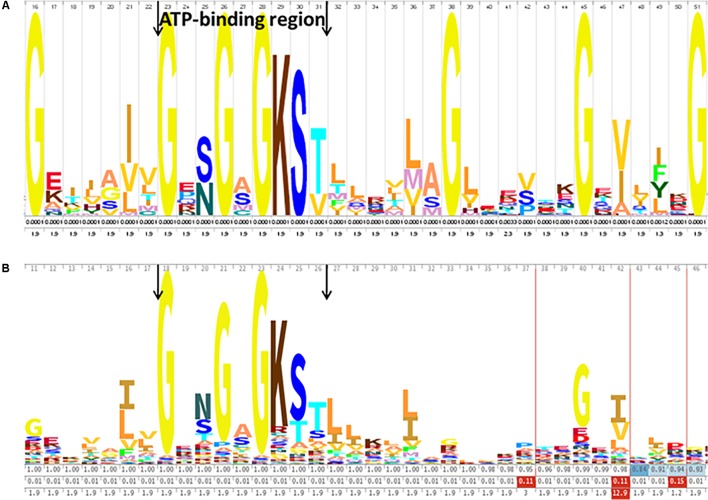
ATP binding cassette (ABC) conserved sequence of the ABC transporters identified in *G. vaginalis*. **(A)** The consensus sequence of the seven ABC transporters identified in the surfome of *G. vaginalis* ATCC14018 strain, represented using WebLogo. **(B)** The hidden Markov model (HMM) logo from the ABC transporter family (Pfam PF00005) is shown. The ABC is shown between the arrows (Walker A motif).

The proteins identified in this study include proteins involved in important functions. Thirteen proteins belonging to the ATP binding cassette (ABC) superfamily were identified, seven of which were classified as ABC transporters using the Pfam server (Supplementary Table S4). ABC transporters are composed of two regions that can be organized into one or two polypeptides, with a highly conserved ABC and a less conserved TMD. The primary sequence of the seven ABC transporters of *G. vaginalis* identified by shaving was analyzed by looking for the typical phosphate-binding loop (Walker A motif), which contained the conserved lysine amino acid (**Figure 4**). The Walker A motif GXXGXGKS/T (where X represents any residue) was clearly observed in this family (Rees et al., 2009). The logo obtained for the seven *G. vaginalis* ABC transporters (**Figure 4A**) was very similar to the logo of the ABC transporter family (Pfam PF00005) (**Figure 4B**).

The 52 *G. vaginalis* proteins identified by the shaving approach and annotated as conserved hypothetical proteins, putative cell surface proteins and hypothetical proteins were analyzed using Pfam. Of these, 38 were mapped to a Pfam family (Supplementary Table S5). The protein sequences were also analyzed with Blastp against the *G. vaginalis* strain ATCC14019, which has a better-annotated genome. Most of the Pfam family predictions and Blastp results were consistent. Remarkably, two proteins were identified as being involved in septum formation (BAQ33018 and BAQ3210), one was identified as being involved in cell division (BAQ32849) and another two were described as proteins with an uncharacterized sugar-binding domain (BAQ32771 and BAQ33606). Two proteins, BAQ33051 and BAQ33368, were found to be membrane proteins in ATCC14019. Furthermore, BAQ33427 was classified as a member of proteins with a *Listeria*-bacteroides repeat domain found in families of internalins of *Listeria* species (Breitsprecher et al., 2014), and BAQ33672 was classified as having a Rib/alpha-like repeat, which is present in bacterial surface proteins of group B streptococci (Larsson et al., 2006).

### Surface Location of GroEL and Cna on the Cell Surface of *G. vaginalis*

We did not found any specific antibodies against *G. vaginalis*’s identified proteins to validate their surface-exposed location by immunodetection. Therefore, two strategies were designed to facilitate experimentation in this study: first, the use of available antibodies against the conserved proteins of other species homologous to those of *G. vaginalis* and, secondly, the production of antibodies against proteins identified in this surfome.

GroEL, FtsZ, and DnaK proteins were interesting proteins identified in the *G. vaginalis* surfome, and antibodies against the homologous proteins of *E. coli* were available. These three proteins were described as cytoplasmic in the bibliography and using the two servers employed in this work to evaluate protein subcellular location. At the same time, GroEL and DnaK were described as moonlighting proteins in other microorganisms (see section “Discussion”) and FtsZ can be found on the surface due to its role in septum formation.

The protein sequences of GroEL, FtsZ, and DnaK of *E. coli* and the homologous *G. vaginalis* proteins are 56, 42, and 56% identical, respectively. Therefore, cellular location on the cell surface of these non-classically secreted proteins might be tested in *G. vaginalis* cells with antibodies against the *E. coli* proteins. For FtsZ and DnaK proteins, an ELISA using these antibodies tested with *G. vaginalis* total extract did not detect specific signal (data not shown).

**FIGURE 5:**
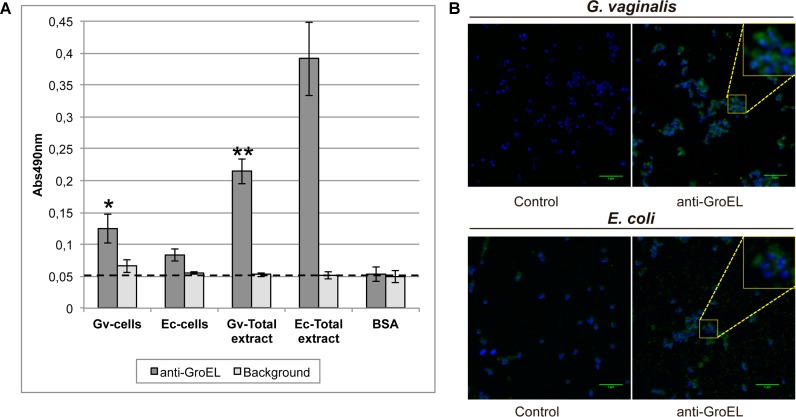
Detection of GroEL on the surface of *G. vaginalis* and *Escherichia coli*. **(A)** ELISA assay is used to detect GroEL on the cell surfaced of *G. vaginalis* and *E. coli*. Total extracts were used as positive controls and BSA represents the background of the antibodies used. Statistically significant differences relative to *E. coli* samples were indicated (^∗^*p* < 0.05, ^∗∗^*p* < 0.01). Each value is presented as the average of two independent experiment results with four replicates. The background represents the signal without any antibody. **(B)** Immunofluorescence assay is used to detect GroEL on the cell surface of *G. vaginalis* and *E. coli*. Control images showed the background of the secondary antibody (anti-rabbit-A488 IgG). Cell nuclei were stained with DAPI in all images (blue color). The green line in the bottom right corner indicates a 5 μm scale.

The surface-exposed location of the chaperone GroEL in intact cells and total protein extracts of *G. vaginalis* and *E. coli* was determined by ELISA and by immunofluorescence (**Figure 5**). The accessibility of GroEL in the ELISA was significantly higher on the cell surface of *G. vaginalis* compared to that of *E. coli*, despite the antibody being specific to *E. coli* (**Figure 5A**). The same result was observed by immunofluorescence, with a more intense signal found for *G. vaginalis* cells than *E. coli* cells (**Figure 5B**). The signal was increased meaningfully when the full protein extracts were tested for both microorganisms, and the GroEL signal observed for *E. coli* was significantly higher than for *G. vaginalis*.

In contrast, two proteins identified in the surfome were selected due to the higher number of peptides detected by mass spectrometry (**Table 1**) and their low similarity with other microbial proteins as determined by Blastp analysis. These proteins are annotated as M protein repeat protein (BAQ32758) and Cna protein B-type domain (BAQ32792). The genes were cloned with a Histidine-tag and the proteins expressed and purified to produce monoclonal antibodies (mAb) against them to check their subcellular location in *G. vaginalis* cells. The expression was made in *E. coli*, but only Cna purification rendered sufficient amounts of the protein to allow mouse immunization. The M protein expression in *E. coli* reduced the growth rate of the bacteria and resulted in a low protein yield.

**FIGURE 6:**
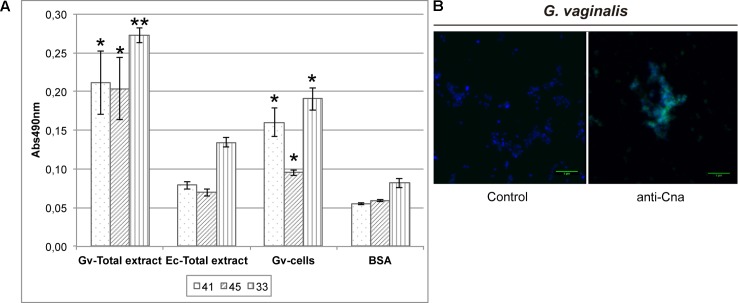
Detection of Cna location on the surface of *G. vaginalis*. **(A)** Analysis of Cna on the surface of *G. vaginalis* is achieved with ELISA using three customized monoclonal antibodies (mAb), number 41, 45, and 33. *E. coli* total extract was used to check the cross-reactivity of the mAb. BSA was used as a negative control in the ELISA. Statistically significant differences relative to *E. coli* sample were indicated (^∗^*p* < 0.05, ^∗∗^*p* < 0.01). Each value is presented as the average of two independent experiment results with four replicates. **(B)** The anti-Cna mAb number 41 was checked by immunofluorescence assay. Control images showed the background of the secondary antibody (anti-mouse-A488 IgG). Cell nuclei were stained with DAPI in all images (blue color). The green line in the bottom right corner indicates a 5 μm scale.

Monoclonal antibodies against Cna were produced as indicated in the Section “Materials and Methods.” The best three mAb (41, 45, and 33) were purified and showed significant differences in terms of the signal obtained with *G. vaginalis* samples compared with the *E. coli* total extract using ELISA (**Figure 6A**). The total protein extract of *E. coli* was used to discard any cross-reactivity of the mAb since the Cna protein was purified from *E. coli.* Furthermore, the specific signal on the surface of *G. vaginalis* was observed by immunofluorescence with the best mAb chosen using the ELISA results (**Figure 6B**).

## Discussion

### *In Silico* Analysis of the Subcellular Location of *G. vaginalis* Identified Proteins

The cell surface shaving procedure followed by an LC-MS/MS analysis identified 261 *G. vaginalis* proteins. To obtain a robust prediction of the subcellular location of *G. vaginalis* identified proteins, they were analyzed using two servers in parallel, PSORT and Gpos-mPLoc, which was specifically designed for Gram-positive bacterial proteins (Shen and Chou, 2009).

Only 3 of the 25 identified proteins with more than 10 peptides (**Table 1**) were predicted to be in different cell compartments according to the server used. BAQ33548 was localized in the plasma membrane using PSORT and in the cytoplasm using Gpos-mPLoc. Both BAQ32849 and BAQ33018 were localized in the cytoplasm with PSORT and in the cell membrane with Gpos-mPLoc (more details in Supplementary Table S2). In the literature, it is common to find discrepancies between the *in silico*-predicted topology and the experimental data (Rodriguez-Ortega et al., 2006; Lee et al., 2015). We only found two discrepancies when comparing the characteristic motifs of surface-exposed proteins predicted using bioinformatics tools with the mapping of the MS identified peptides on the primary sequence of the proteins (Supplementary Table S1 and Supplementary Data). This can be explained due to the poor scores of the surface-exposed motifs predicted, which do not represent the physiological situation of these proteins. The discrepancies can be resolved when these proteins or a homolog have a well-known structure, which helps to discern which part of the protein is exposed to the extracellular medium.

Interestingly, two of the proteins included in **Table 1** were classified as lipoproteins. Lipoproteins can be secreted or incorporated into the plasma membrane outer leaflet in Gram-positive bacteria (Zuckert, 2014). The lipid modification of lipoproteins served to retain these lipoproteins in the membrane or cell wall interface; however, a previous study showed that the lipobox motif can be removed at the conserved cysteine residue, resulting in the release of the unmodified mature lipoprotein into the growth medium (Krishnappa et al., 2013). Consistent with these findings, we did not identify any peptide from the lipobox domains (Supplementary Data). These regions can also be protected from trypsin digestion if they are inserted into the membrane. Furthermore, a surface-associated HtrA protein was identified, which is known to play a relevant role as a chaperone and protease, and cleaves several lipoproteins from the cell surface in *Bacillus subtilis* (Krishnappa et al., 2013).

Different protein motifs typical of surface-exposed proteins were detected in 80 of the 261 identified proteins (SP, LPXTG PG-anchoring motif, lipobox domain and TMD). Most comprise TMDs or SPs. Regarding the detected SPs, most are for the Sec secretion pathway. The SPs identified for the Tat secretion pathway are typical of proteins that are secreted in a completely folded state or as cofactors (Song et al., 2015). The lipo-SP corresponded to the lipoprotein SP of the Gram-positive bacteria (Bagos et al., 2008). The LPXTG motif was detected in five of the identified proteins in *G. vaginalis*. The LPXTG motif is responsible for the covalent attachment of proteins to the PG layer by sortase enzymes (Hendrickx et al., 2011). Sortases are integral membrane proteins responsible for recognizing and cleaving the carboxyl-terminal sorting signal (LPXTG). In the comparative genomic analysis of the ATCC14019 strain of *G. vaginalis*, 4 sortase enzymes, and 13 LPXTG proteins were identified (Yeoman et al., 2010). Moreover, four sortase enzymes have been identified by Blastp in the ATCC14018 genome (BAQ32669, BAQ33004, BAQ33565, and BAQ33653), which were 100% identical to the corresponding enzymes in the ATCC14019 genome. However, the identification of these genes did not ensure their expression under the conditions tested in the present study. Likewise, the failure to detect more proteins with the LPXTG motif may be related to their low abundance in the cell wall and high hydrophobicity.

### Non-classical Secreted Proteins or Moonlighting Proteins

Several cytoplasmic proteins without any predicted export/retention signals have been identified in the surfome of different bacteria. These proteins are classified as being cytoplasmic proteins; however, they are more correctly named non-classical secreted proteins (Bendtsen et al., 2005). Of the 261 identified proteins, 70% were classified as cytoplasmic, a result comparable to the findings obtained in relation to other Gram-positive and negative bacterial surfomes (Olaya-Abril et al., 2014). Furthermore, some cytoplasmic proteins are described as moonlighting due to their different functions according to their subcellular location. Interestingly, a meta-analysis of many surface proteomics studies reveals novel candidates for intracellular/surface moonlighting proteins in Gram-positive and negative bacteria (Wang and Jeffery, 2016). Many of these proteins, found on the surface of bacteria and classified as intracellular, are involved in central metabolic pathways or stress responses if found in the cytoplasm, as this work attests. We identified several *Gardnerella* proteins homologous to moonlighting proteins described in other Gram-positive microorganisms involved in metabolism, such as enolase (Eno) (Kainulainen and Korhonen, 2014; Wang et al., 2014), glyceraldehyde-3-phosphate dehydrogenase (Gap) (Henderson and Martin, 2011; Wang et al., 2014), phosphoglycerate mutase (GpmA), inosine 5′-monophosphate dehydrogenase (IMPDH) (Kainulainen and Korhonen, 2014) and pyruvate kinase (PyK) (Henderson and Martin, 2011; Kainulainen and Korhonen, 2014). Also, certain relevant chaperones, such as DnaK (Kainulainen and Korhonen, 2014; Wang et al., 2014) and GroEL (Bendtsen et al., 2005; Kainulainen and Korhonen, 2014), were identified. The co-chaperonin GroES is not described to be a classical moonlighting protein; however, it forms a cytoplasmic complex with GroEL, which is a moonlighting protein (Xu et al., 1997). This finding supports a previous study that identified GroES and GroEL on the surface of *Lactobacillus rhamnosus* using a shaving approach (Espino et al., 2015). GroEL was described as part of the interactions between microorganisms and insect (Kupper et al., 2014). The elongation factors Tu (EF-Tu) (Kainulainen and Korhonen, 2014; Wang et al., 2014) and G (EF-G) and the protein translocase subunit A (SecA) (Kainulainen and Korhonen, 2014) were also identified. Notably, in yeast cells, metabolic proteins, chaperones or stress-related proteins and elongation factors are also consistently identified as surface proteins since many are moonlighting proteins, as recently reported in relation to the opportunistic pathogen (Gil-Bona et al., 2015, 2017; Marin et al., 2015).

In bacteria, through non-classical secretion, these proteins can reach the surface of the microorganism or the extracellular media, developing important roles in virulence, modulating the host immune response and adhesion to or competition with other bacteria. This is due to protein’s ability to bind to several components of the host, such as plasminogen and salivary mucin, or other bacteria (Dallo et al., 2002; Bendtsen et al., 2005; Henderson and Martin, 2011; Kainulainen and Korhonen, 2014; Wang et al., 2014; Espino et al., 2015). Curiously, some moonlighting proteins of *Candida albicans* also have the ability to bind plasminogen, which is relevant to infection (Jong et al., 2003). As previously stated, in-depth analysis based on 22 surface proteomics studies, elaborated with 10 Gram-negative and 12 Gram-positive microorganisms, was undertaken by Wang and Jeffery (2016). The authors examine the relevance of bacterial cell surface in infection and virulence and their study can be applied in vaccine and biomarker development.

In the *in silico* analysis presented in this work, of the 17 *G. vaginalis* surface proteins identified and described as moonlighting in other microorganisms, 15 were classified as “inside” and only 2 (Eno and IMPDH) were identified as “both,” indicating that the bioinformatic tools do not predict the extracellular location of this type of proteins in most cases. Interestingly, the two servers (PSORT and Gpos-mPLoc) classified SecA (BAQ33096) as “inside” and AtpD as “both,” while according to the Universal Protein Resource database (Uniprot), these proteins are located in the cell membrane, as peripheral membrane proteins. SecA is a peripheral component of the membrane translocon SecYEG, which mediates the general secretion pathway across the cytoplasmic membrane (Randall et al., 2005), which explains their detection using our shaving approach. Another discrepancy of the predicted locations was observed for FtsY (BAQ33899), as although it was classified as “outside,” it is also known to be involved in protein secretion across the plasma membrane and located in both the cytoplasm and the plasma membrane inner leaflet (Angelini et al., 2005). FtsZ and FtsE were classified as located in the cytoplasm, and both proteins were involved in septum formation and assembling the cytoplasmic membrane, which may explain why these proteins were found to be surface-exposed (Huang et al., 2013). During cell division, due to septum formation and remodeling of the cell wall, some of the cytoplasm components are released into the medium and exposed on the cell surface.

### The ABC Superfamily, Peptidoglycan-Related Proteins and Hypothetical Proteins

Seven proteins identified in this study belong to the ATP binding cassette (ABC) superfamily. The analysis of their sequence showed that they include a typical phosphate-binding loop (Walker A motif). The strong similarity between the logo obtained for the *G. vaginalis* ABC transporters and the logo of the ABC transporter family (Pfam PF00005) demonstrates that this domain is highly conserved in *G. vaginalis*.

A different group of relevant membrane-associated proteins are the penicillin-binding proteins. Among the *G. vaginalis* proteins identified, two penicillin-binding proteins were identified (BAQ32970 and BAQ32781). In Gram-positive microorganisms, these proteins can selectively interact and non-covalently bind to penicillin or any other antibiotic that contains a condensed beta-lactam thiazolidine ring. Therefore, these proteins play an important role in pathogenesis due to their contribution to the development of antibiotic resistance. Interestingly, four proteins involved in PG biosynthesis, essential for the integrity of the cell wall, were also identified: DdI, MurA, MurD, and MurC.

In addition, the *in silico* analysis of the hypothetical proteins identified in this work revealed noteworthy results as some of the proteins have typical roles or domains found in cell-wall associated proteins. There are proteins involved in cell division and septum formation, proteins with an uncharacterized sugar-binding domain, with a *Listeria*-bacteroides domain of internalins and having a Rib/alpha-like repeat. These analyses support the location of the *G. vaginalis* identified proteins on the surface as found using the shaving proteomic approach.

### Validation of *G. vaginalis* Surface Proteins

The data presented above supports our ability to identify many relevant *G. vaginalis* surface proteins, even though most are classified as located inside the cell by the bioinformatic tools. However, as in any other proteomic analysis, the validation assays are of outstanding interest. Also, it must be considered that, although cell lysis controls were introduced, a very low level of contamination with intracellular proteins remains possible. For these reasons, and despite of the lack of antibodies, the surface localization of the GroEL chaperone and Cna were tested using immunodetection. Surprisingly, using the antibodies anti GroEL from *E. coli*, the signal intensities obtained with *G. vaginalis* were higher than with *E. coli* cells. The good recognition of *G. vaginalis* GroEL at the cell surface may be due to *G. vaginalis* being a Gram-positive bacterium with a thin PG layer as its cell envelope is more permeable for protein secretion and/or the accessibility of antibodies. GroEL has been found on the surface of several Gram-positive microorganisms, including *Clostridium difficile* (Hennequin et al., 2001), *Mycobacterium tuberculosis* (DnaK was also identified) (Hickey et al., 2009), *Bacillus anthracis* (Somani et al., 2016) and *Lactobacillus rhamnosus* as stated above (Espino et al., 2015). Furthermore, GroEL and DnaK were found as part of the cell wall and secreted in *Streptococcus pyogenes* (Cole et al., 2005). For the immunodetection of Cna, 1 of the 25 more abundant proteins detected by shaving at the *G. vaginalis* cell surface, mAb were generated. The Cna of *Staphylococcus aureus* is a collagen-binding surface protein with a B-type domain. Cna has a collagen-binding domain that is necessary and sufficient for *S. aureus* cells to adhere to cartilage (Patti et al., 1994). Cna is also able to attach to complement system protein C1q and to the extracellular matrix protein laminin (Valotteau et al., 2017). For these reasons, the generated antibodies are a useful tool for studying the putative role of *G. vaginalis* Cna in pathogenesis, and they would be also useful in the development of future diagnostic immunoassays for BV in combination with antibodies against other of the anaerobes present in this disorder. Immunochromatography assays are easy and rapid (approximately 15 min) and they would be alternative methods to other diagnostic assays as qPCR (Kikuta et al., 2008). In some cases, they show up less sensitivity and specificity than qPCR; but, they represent an interesting alternative that do not require equipment or experienced personal. Thus, a new immunochromatography assay could be developed not specifically to be used in hospitals but as a point of care diagnostic test also in developing countries.

## Conclusion

This study represents the first proteomic approach adopted to investigate the surface of *G. vaginalis*, one of the main etiological agents responsible for BV. Cell surface trypsin shaving and LC-MS/MS analysis allowed the identification of 261 surface-associated proteins of *G. vaginalis*. Bioinformatics tools were used to provide a comprehensive analysis of the motifs characteristic of surface-exposed proteins, and 80 *G. vaginalis* proteins were found to have these motifs. Among these, 36 proteins had a SP motif, 17 had a lipobox domain, 5 proteins had a LPXTG motif, 56 proteins had a TMD, 7 proteins had 2 TMDs and 3 proteins had 6 or more TMDs. Furthermore, close to one third of the identified proteins were classified as surface-exposed proteins by the PSORT server. Subcellular location was also analyzed using the Gpos-mPLoc server, which validated the classification of half of the surface-exposed proteins found by the PSORT server. Moreover, the surface location of GroEL and Cna was validated by ELISA and immunofluorescence assays. mAb against *G. vaginalis* Cna could be a useful tool to enable the identification of this microorganism in biological samples and for further studies of *G. vaginalis*, considering the narrow availability of specific antibodies. To conclude, these results contribute to our understanding of this fastidious and poorly understood microorganism.

## Ethics Statement

BALB/c mice were maintained under specific pathogen-free conditions and handled in laminar-flow isolation hoods in the Animal Facility Unit of the Comitè d’Ètica d’Experimentació Animal (PCB). All the animal manipulations were performed under the experimental protocol approved by the Comitè d’Ètica d’Experimentació Animal del PCB, CEEA-PCB no. 9154-P1.

## Author Contributions

EM designed and performed the experiments, analysis of results, and writing of the manuscript. AH performed the experiments and analysis results. LP performed the mice immunization experiments and analysis of results with antibodies. JA performed the mice immunization experiments and analysis of results with antibodies. MH performed the analysis of results. LM designed the experiments, analysis of results, and writing of the manuscript. CG designed the experiments, analysis of results, and critically revised the manuscript.

## Conflict of Interest Statement

The authors declare that the research was conducted in the absence of any commercial or financial relationships that could be construed as a potential conflict of interest.

## Footnotes

1 http://www.ncbi.nlm.nih.gov/nuccore/AP012332.1

2 https://www.ebi.ac.uk/pride/archive/

3 http://www.cbs.dtu.dk/services/SignalP

4 http://www.cbs.dtu.dk/services/TatP

5 http://www.compgen.org/tools/PRED-LIPO

6 http://www.cbs.dtu.dk/services/TMHMM

7 https://npsa-prabi.ibcp.fr

8 http://bamics2.cmbi.ru.nl/websoftware/locatep2/locatep2_start.php

9 http://psort.hgc.jp/form.html

10 http://www.csbio.sjtu.edu.cn/bioinf/Gpos-multi/

11 http://wlab.ethz.ch/protter/start/

12 http://pfam.xfam.org/

13 https://blast.ncbi.nlm.nih.gov/Blast.cgi?PAGE=Proteins

14 http://weblogo.berkeley.edu/logo.cgi

## References

[B1] Angelini S. Deitermann S. Koch H. G. (2005). FtsY, the bacterial signal-recognition particle receptor, interacts functionally and physically with the SecYEG translocon. 6 476–481. 10.1038/sj.embor.7400385 15815684 PMC1299298

[B2] Atashili J. Poole C. Ndumbe P. M. Adimora A. A. Smith J. S. (2008). Bacterial vaginosis and HIV acquisition: a meta-analysis of published studies. 22 1493–1501. 10.1097/QAD.0b013e3283021a37 18614873 PMC2788489

[B3] Bagos P. G. Tsirigos K. D. Liakopoulos T. D. Hamodrakas S. J. (2008). Prediction of lipoprotein signal peptides in Gram-positive bacteria with a Hidden Markov Model. 7 5082–5093. 10.1021/pr800162c 19367716

[B4] Bendtsen J. D. Kiemer L. Fausboll A. Brunak S. (2005). Non-classical protein secretion in bacteria. 5:58. 10.1186/1471-2180-5-58 16212653 PMC1266369

[B5] Breitsprecher D. Gherardi E. Bleymuller W. M. Niemann H. H. (2014). Crystal structure of an engineered YopM-InlB hybrid protein. 14:12. 10.1186/1472-6807-14-12 24669959 PMC3986869

[B6] Bretelle F. Rozenberg P. Pascal A. Favre R. Bohec C. Loundou A. (2015). High *Atopobium vaginae* and *Gardnerella vaginalis* vaginal loads are associated with preterm birth. 60 860–867. 10.1093/cid/ciu966 25452591

[B7] Catlin B. W. (1992). *Gardnerella vaginalis*: characteristics, clinical considerations, and controversies. 5 213–237. 1498765 10.1128/cmr.5.3.213 PMC358241

[B8] Cole J. N. Ramirez R. D. Currie B. J. Cordwell S. J. Djordjevic S. P. Walker M. J. (2005). Surface analyses and immune reactivities of major cell wall-associated proteins of group a streptococcus. 73 3137–3146. 10.1128/IAI.73.5.3137-3146.2005 15845522 PMC1087385

[B9] Dalbey R. E. Wang P. Van Dijl J. M. (2012). Membrane proteases in the bacterial protein secretion and quality control pathway. 76 311–330. 10.1128/MMBR.05019-11 22688815 PMC3372248

[B10] Dallo S. F. Kannan T. R. Blaylock M. W. Baseman J. B. (2002). Elongation factor Tu and E1 beta subunit of pyruvate dehydrogenase complex act as fibronectin binding proteins in *Mycoplasma pneumoniae*. 46 1041–1051. 10.1046/j.1365-2958.2002.03207.x 12421310

[B11] Espino E. Koskenniemi K. Mato-Rodriguez L. Nyman T. A. Reunanen J. Koponen J. (2015). Uncovering surface-exposed antigens of *Lactobacillus rhamnosus* by cell shaving proteomics and two-dimensional immunoblotting. 14 1010–1024. 10.1021/pr501041a 25531588

[B12] Gardner H. L. Dukes C. D. (1955). *Haemophilus vaginalis* vaginitis: a newly defined specific infection previously classified non-specific vaginitis. 69 962–976. 14361525

[B13] Giakoumelou S. Wheelhouse N. Cuschieri K. Entrican G. Howie S. E. Horne A. W. (2015). The role of infection in miscarriage. 22 116–133. 10.1093/humupd/dmv041 26386469 PMC4664130

[B14] Gil-Bona A. Amador-Garcia A. Gil C. Monteoliva L. (2017). The external face of *Candida albicans*: a proteomic view of the cell surface and the extracellular environment. 10.1016/j.jprot.2017.12.002 [Epub ahead of print]. 29223801

[B15] Gil-Bona A. Parra-Giraldo C. M. Hernaez M. L. Reales-Calderon J. A. Solis N. V. Filler S. G. (2015). *Candida albicans* cell shaving uncovers new proteins involved in cell wall integrity, yeast to hypha transition, stress response and host-pathogen interaction. 127 340–351. 10.1016/j.jprot.2015.06.006 26087349 PMC4721921

[B16] Goosens V. J. Monteferrante C. G. Van Dijl J. M. (2014). The Tat system of Gram-positive bacteria. 1843 1698–1706. 10.1016/j.bbamcr.2013.10.008 24140208

[B17] Henderson B. Martin A. (2011). Bacterial virulence in the moonlight: multitasking bacterial moonlighting proteins are virulence determinants in infectious disease. 79 3476–3491. 10.1128/IAI.00179-11 21646455 PMC3165470

[B18] Hendrickx A. P. Budzik J. M. Oh S. Y. Schneewind O. (2011). Architects at the bacterial surface - sortases and the assembly of pili with isopeptide bonds. 9 166–176. 10.1038/nrmicro2520 21326273

[B19] Hennequin C. Collignon A. Karjalainen T. (2001). Analysis of expression of GroEL (Hsp60) of *Clostridium difficile* in response to stress. 31 255–260. 10.1006/mpat.2001.0468 11710845

[B20] Hernaez M. L. Ximenez-Embun P. Martinez-Gomariz M. Gutierrez-Blazquez M. D. Nombela C. Gil C. (2010). Identification of *Candida albicans* exposed surface proteins in vivo by a rapid proteomic approach. 73 1404–1409. 10.1016/j.jprot.2010.02.008 20167299

[B21] Hickey T. B. Thorson L. M. Speert D. P. Daffe M. Stokes R. W. (2009). *Mycobacterium tuberculosis* Cpn60.2 and DnaK are located on the bacterial surface, where Cpn60.2 facilitates efficient bacterial association with macrophages. 77 3389–3401. 10.1128/IAI.00143-09 19470749 PMC2715658

[B22] Hilbert D. W. Smith W. L. Chadwick S. G. Toner G. Mordechai E. Adelson M. E. (2016). Development and validation of a highly accurate quantitative real-time PCR assay for diagnosis of bacterial vaginosis. 54 1017–1024. 10.1128/JCM.03104-15 26818677 PMC4809904

[B23] Huang K. H. Durand-Heredia J. Janakiraman A. (2013). FtsZ ring stability: of bundles, tubules, crosslinks, and curves. 195 1859–1868. 10.1128/JB.02157-12 23457247 PMC3624584

[B24] Janulaitiene M. Paliulyte V. Grinceviciene S. Zakareviciene J. Vladisauskiene A. Marcinkute A. (2017). Prevalence and distribution of *Gardnerella vaginalis* subgroups in women with and without bacterial vaginosis. 17:394. 10.1186/s12879-017-2501-y 28583109 PMC5460423

[B25] Jong A. Y. Chen S. H. Stins M. F. Kim K. S. Tuan T. L. Huang S. H. (2003). Binding of *Candida albicans* enolase to plasmin(ogen) results in enhanced invasion of human brain microvascular endothelial cells. 52 615–622. 10.1099/jmm.0.05060-0 12867553

[B26] Jung H. S. Ehlers M. M. Lombaard H. Redelinghuys M. J. Kock M. M. (2017). Etiology of bacterial vaginosis and polymicrobial biofilm formation. 43 651–667. 10.1080/1040841X.2017.1291579 28358585

[B27] Kainulainen V. Korhonen T. K. (2014). Dancing to another tune-adhesive moonlighting proteins in bacteria. 3 178–204. 10.3390/biology3010178 24833341 PMC4009768

[B28] Kaul R. Nagelkerke N. J. Kimani J. Ngugi E. Bwayo J. J. Macdonald K. S. (2007). Prevalent herpes simplex virus type 2 infection is associated with altered vaginal flora and an increased susceptibility to multiple sexually transmitted infections. 196 1692–1697. 10.1086/522006 18008255

[B29] Kavoussi S. K. Pearlman M. D. Burke W. M. Lebovic D. I. (2006). Endometrioma complicated by tubo-ovarian abscess in a woman with bacterial vaginosis. 2006:84140. 10.1155/IDOG/2006/84140 17485813 PMC1779615

[B30] Kenyon C. R. Osbak K. (2014). Recent progress in understanding the epidemiology of bacterial vaginosis. 26 448–454. 10.1097/GCO.0000000000000112 25304606

[B31] Kikuta H. Sakata C. Gamo R. Ishizaka A. Koga Y. Konno M. (2008). Comparison of a lateral-flow immunochromatography assay with real-time reverse transcription-PCR for detection of human metapneumovirus. 46 928–932. 10.1128/JCM.01888-07 18174301 PMC2268371

[B32] Kohler G. Milstein C. (1975). Continuous cultures of fused cells secreting antibody of predefined specificity. 256 495–497. 10.1038/256495a0 1172191

[B33] Koumans E. H. Sternberg M. Bruce C. Mcquillan G. Kendrick J. Sutton M. (2007). The prevalence of bacterial vaginosis in the United States, 2001-2004; associations with symptoms, sexual behaviors, and reproductive health. 34 864–869. 10.1097/OLQ.0b013e318074e565 17621244

[B34] Kovacs-Simon A. Titball R. W. Michell S. L. (2011). Lipoproteins of bacterial pathogens. 79 548–561. 10.1128/IAI.00682-10 20974828 PMC3028857

[B35] Krishnappa L. Dreisbach A. Otto A. Goosens V. J. Cranenburgh R. M. Harwood C. R. (2013). Extracytoplasmic proteases determining the cleavage and release of secreted proteins, lipoproteins, and membrane proteins in *Bacillus subtilis*. 12 4101–4110. 10.1021/pr400433h 23937099

[B36] Kupper M. Gupta S. K. Feldhaar H. Gross R. (2014). Versatile roles of the chaperonin GroEL in microorganism-insect interactions. 353 1–10. 10.1111/1574-6968.12390 24460534

[B37] Larsson C. Lindroth M. Nordin P. Stalhammar-Carlemalm M. Lindahl G. Krantz I. (2006). Association between low concentrations of antibodies to protein alpha and Rib and invasive neonatal group B streptococcal infection. 91 F403–F408. 10.1136/adc.2005.090472 17056838 PMC2672751

[B38] Lee C. H. Chou C. C. Hsu M. F. Wang A. H. (2015). Determining the N-terminal orientations of recombinant transmembrane proteins in the *Escherichia coli* plasma membrane. 5:15086. 10.1038/srep15086 26462555 PMC4604451

[B39] Machado A. Cerca N. (2015). Influence of biofilm formation by *Gardnerella vaginalis* and other anaerobes on bacterial vaginosis. 212 1856–1861. 10.1093/infdis/jiv338 26080369

[B40] Machado A. Jefferson K. K. Cerca N. (2013). Interactions between *Lactobacillus crispatus* and Bacterial Vaginosis (BV)-associated bacterial species in initial attachment and biofilm formation. 14 12004–12012. 10.3390/ijms140612004 23739678 PMC3709769

[B41] Marin E. Parra-Giraldo C. M. Hernandez-Haro C. Hernaez M. L. Nombela C. Monteoliva L. (2015). *Candida albicans* shaving to profile human serum proteins on hyphal surface. 6:1343. 10.3389/fmicb.2015.01343 26696967 PMC4672057

[B42] Masson L. Mlisana K. Little F. Werner L. Mkhize N. N. Ronacher K. (2014). Defining genital tract cytokine signatures of sexually transmitted infections and bacterial vaginosis in women at high risk of HIV infection: a cross-sectional study. 90 580–587. 10.1136/sextrans-2014-051601 25107710

[B43] Menard J. P. Fenollar F. Henry M. Bretelle F. Raoult D. (2008). Molecular quantification of *Gardnerella vaginalis* and *Atopobium vaginae* loads to predict bacterial vaginosis. 47 33–43. 10.1086/588661 18513147

[B44] Navarre W. W. Schneewind O. (1999). Surface proteins of gram-positive bacteria and mechanisms of their targeting to the cell wall envelope. 63 174–229. 10.1128/mmbr.63.1.174-229.1999 PMC98962 10066836

[B45] Olaya-Abril A. Gomez-Gascon L. Jimenez-Munguia I. Obando I. Rodriguez-Ortega M. J. (2012). Another turn of the screw in shaving Gram-positive bacteria: optimization of proteomics surface protein identification in *Streptococcus pneumoniae*. 75 3733–3746. 10.1016/j.jprot.2012.04.037 22575384

[B46] Olaya-Abril A. Jimenez-Munguia I. Gomez-Gascon L. Rodriguez-Ortega M. J. (2014). Surfomics: shaving live organisms for a fast proteomic identification of surface proteins. 97 164–176. 10.1016/j.jprot.2013.03.035 23624344

[B47] Oshima K. Hisamatsu S. Toh H. Nakano A. Kiuchi M. Kuroyanagi H. (2015). Complete genome sequence of *Gardnerella vaginalis* strain JCM 11026T, isolated from vaginal tracts of women. 3:e00286-15. 10.1128/genomeA.00286-15 25858849 PMC4392161

[B48] Patti J. M. Bremell T. Krajewska-Pietrasik D. Abdelnour A. Tarkowski A. Ryden C. (1994). The *Staphylococcus aureus* collagen adhesin is a virulence determinant in experimental septic arthritis. 62 152–161. 8262622 10.1128/iai.62.1.152-161.1994 PMC186080

[B49] Peres A. L. Camarotti J. R. Cartaxo M. Alencar N. Stocco R. C. Becak W. (2015). Molecular analysis and conventional cytology: association between HPV and bacterial vaginosis in the cervical abnormalities of a Brazilian population. 14 9497–9505. 10.4238/2015.August.14.13 26345883

[B50] Petersen T. N. Brunak S. Von Heijne G. Nielsen H. (2011). SignalP 4.0: discriminating signal peptides from transmembrane regions. 8 785–786. 10.1038/nmeth.1701 21959131

[B51] Randall L. L. Crane J. M. Lilly A. A. Liu G. Mao C. Patel C. N. (2005). Asymmetric binding between SecA and SecB two symmetric proteins: implications for function in export. 348 479–489. 10.1016/j.jmb.2005.02.036 15811382

[B52] Rees D. C. Johnson E. Lewinson O. (2009). ABC transporters: the power to change. 10 218–227. 10.1038/nrm2646 19234479 PMC2830722

[B53] Rodriguez-Ortega M. J. Norais N. Bensi G. Liberatori S. Capo S. Mora M. (2006). Characterization and identification of vaccine candidate proteins through analysis of the group A *Streptococcus* surface proteome. 24 191–197. 10.1038/nbt1179 16415855

[B54] Sabour S. Arzanlou M. Vaez H. Rahimi G. Sahebkar A. Khademi F. (2018). Prevalence of bacterial vaginosis in pregnant and non-pregnant Iranian women: a systematic review and meta-analysis. 297 1101–1113. 10.1007/s00404-018-4722-8 29455377

[B55] Schneewind O. Missiakas D. (2014). Sec-secretion and sortase-mediated anchoring of proteins in Gram-positive bacteria. 1843 1687–1697. 10.1016/j.bbamcr.2013.11.009 24269844 PMC4031296

[B56] Schneewind O. Missiakas D. M. (2012). Protein secretion and surface display in Gram-positive bacteria. 367 1123–1139. 10.1098/rstb.2011.0210 22411983 PMC3297441

[B57] Shen H. B. Chou K. C. (2009). Gpos-mPLoc: a top-down approach to improve the quality of predicting subcellular localization of Gram-positive bacterial proteins. 16 1478–1484. 20001911 10.2174/092986609789839322

[B58] Sobel J. D. (2000). Bacterial vaginosis. 51 349–356. 10.1146/annurev.med.51.1.349 10774469

[B59] Somani V. K. Aggarwal S. Singh D. Prasad T. Bhatnagar R. (2016). Identification of novel raft marker protein, FlotP in *Bacillus anthracis*. 7:169. 10.3389/fmicb.2016.00169 26925042 PMC4756111

[B60] Song Y. Nikoloff J. M. Zhang D. (2015). Improving protein production on the level of regulation of both expression and secretion pathways in *Bacillus subtilis*. 25 963–977. 10.4014/jmb.1501.01028 25737123

[B61] St John E. Mares D. Spear G. T. (2007). Bacterial vaginosis and host immunity. 4 22–28. 10.1007/s11904-007-0004-y 17338857

[B62] Sutcliffe I. C. Harrington D. J. (2002). Pattern searches for the identification of putative lipoprotein genes in Gram-positive bacterial genomes. 148 2065–2077. 10.1099/00221287-148-7-2065 12101295

[B63] Valotteau C. Prystopiuk V. Pietrocola G. Rindi S. Peterle D. De Filippis V. (2017). Single-cell and single-molecule analysis unravels the multifunctionality of the *Staphylococcus aureus* collagen-binding protein Cna. 11 2160–2170. 10.1021/acsnano.6b08404 28151647

[B64] Vialas V. Perumal P. Gutierrez D. Ximenez-Embun P. Nombela C. Gil C. (2012). Cell surface shaving of *Candida albicans* biofilms, hyphae, and yeast form cells. 12 2331–2339. 10.1002/pmic.201100588 22685022

[B65] Vizcaino J. A. Deutsch E. W. Wang R. Csordas A. Reisinger F. Rios D. (2014). ProteomeXchange provides globally coordinated proteomics data submission and dissemination. 32 223–226. 10.1038/nbt.2839 24727771 PMC3986813

[B66] Wang G. Xia Y. Cui J. Gu Z. Song Y. Chen Y. Q. (2014). The roles of moonlighting proteins in bacteria. 16 15–22. 23872606

[B67] Wang W. Jeffery C. J. (2016). An analysis of surface proteomics results reveals novel candidates for intracellular/surface moonlighting proteins in bacteria. 12 1420–1431. 10.1039/c5mb00550g 26938107

[B68] Xu Z. Horwich A. L. Sigler P. B. (1997). The crystal structure of the asymmetric GroEL-GroES-(ADP)7 chaperonin complex. 388 741–750. 10.1038/41944 9285585

[B69] Yeoman C. J. Yildirim S. Thomas S. M. Durkin A. S. Torralba M. Sutton G. (2010). Comparative genomics of *Gardnerella vaginalis* strains reveals substantial differences in metabolic and virulence potential. 5:e12411. 10.1371/journal.pone.0012411 20865041 PMC2928729

[B70] Zinnemann K. Turner G. C. (1963). The taxonomic position of “*Haemophilus vaginalis*” [*Corynebacterium vaginale*]. 85 213–219.

[B71] Zuckert W. R. (2014). Secretion of bacterial lipoproteins: through the cytoplasmic membrane, the periplasm and beyond. 1843 1509–1516. 10.1016/j.bbamcr.2014.04.022 24780125 PMC4070597

