# Supplementary material for: Unraveling Gardnerella vaginalis Surface Proteins Using Cell Shaving Proteomics
Source: Front Microbiol. 2018 May 15;9:975. doi: 10.3389/fmicb.2018.00975 (PMC5962675; doi:10.3389/fmicb.2018.00975)
Supplement: Supplementary file 1 [file Table_1.DOCX]

**Table S1.** List of proteins identified in *G. vaginalis* surfome:

| Protein_ID^a^ | Gene name^a^ | Description^a^ | Replicates  (Peptides)^b^ | SP^c^ | Domain^d^ | TMD^e^ |
| --- | --- | --- | --- | --- | --- | --- |
| BAQ32657 | *gyrB* | DNA gyrase subunit B | 3 (5,2,4) |  |  |  |
| BAQ32662 |  | conserved hypothetical protein | 3 (2,2,1) |  |  |  |
| BAQ32664 |  | putative phosphatase | 2 (2,2) |  |  | 1 |
| BAQ32680 |  | phosphoenolpyruvate carboxylase | 3 (2,1,1) |  | lipobox | 1 |
| BAQ32685 |  | conserved hypothetical protein | 3 (3,2,4) |  |  |  |
| BAQ32687 |  | ferredoxin/ferredoxin-NADP reductase | 3 (14,11,5) |  |  |  |
| BAQ32694 |  | hypothetical protein | 3 (5,4,5) | yes | LPXTG | 1 |
| BAQ32696 |  | conserved hypothetical protein | 3 (4,3,4) | yes |  | 1 |
| BAQ32710 | *htrA* | serine protease | 3 (2,1,3) |  |  | 1 |
| BAQ32713 |  | RNA methyltransferase | 3 (2,2,1) |  |  |  |
| BAQ32724 |  | 2-dehydropantoate 2-reductase | 3 (6,6,2) |  |  |  |
| BAQ32758 |  | M protein repeat protein | 3 (46,48,64) | yes |  | 1 |
| BAQ32759 | *pulA* | pullulanase precursor | 3 (2,1,2) |  |  | 1 |
| BAQ32762 |  | putative ABC transporter substrate binding component | 3 (9,8,6) | yes | lipobox | 1 |
| BAQ32771 |  | conserved hypothetical protein | 3 (23,21,23) | yes |  | 2 |
| BAQ32776 | *dnaK** | chaperone protein DnaK | 3 (3,3,2) |  |  |  |
| BAQ32777 | *grpE* | chaperone protein GrpE | 3 (2,2,3) |  |  |  |
| BAQ32780 |  | conserved hypothetical protein | 3 (4,4,3) |  |  |  |
| BAQ32781 |  | putative penicillin-binding protein | 3 (1,1,2) | yes |  | 2 |
| BAQ32789 |  | conserved hypothetical protein | 3 (3,4,2) |  |  |  |
| BAQ32791 |  | formate-tetrahydrofolate ligase | 3 (10,3,9) |  |  | 1 |
| BAQ32792 |  | Cna protein B-type domain-containing protein | 3 (9,8,22) | yes |  | 1 |
| BAQ32799 |  | aminopeptidase C | 3 (2,1,4) |  |  |  |
| BAQ32802 |  | dipeptide/oligopeptide ABC transporter ATP-binding component | 2 (10,3) |  |  | 1 |
| BAQ32803 |  | dipeptide/oligopeptide ABC transporter substrate binding component | 3 (22,19,21) | yes | lipobox | 1 |
| BAQ32810 |  | carbohydrate kinase | 3 (4,4,2) | yes | lipobox |  |
| BAQ32812 |  | 6-phosphogluconate dehydrogenase-like protein | 3 (6,5,6) |  |  |  |
| BAQ32815 |  | hypothetical protein | 3 (4,4,8) | yes |  | 1 |
| BAQ32817 |  | hypothetical protein | 3 (2,1,2) | yes |  | 1 |
| BAQ32818 |  | putative ABC transporter substrate binding component | 3 (14,11,11) | yes | lipobox |  |
| BAQ32822 | *gpsA* | glycerol-3-phosphate dehydrogenase | 3 (7,6,6) | yes |  | 1 |
| BAQ32823 | *ddl* | D-alanine-D-alanine ligase | 3 (4,2,2) |  | lipobox |  |
| BAQ32826 |  | homoserine dehydrogenase | 3 (4,3,3) |  |  |  |
| BAQ32829 |  | hypothetical protein | 3 (8,5,9) | yes |  | 1 |
| BAQ32831 |  | ribonuclease G | 3 (7,6,5) |  |  |  |
| BAQ32832 | *rplU* | 50S ribosomal protein L21 | 3 (6,5,5) |  |  |  |
| BAQ32833 | *rpmA* | 50S ribosomal protein L27 | 3 (6,5,3) |  |  |  |
| BAQ32834 | *obg* | GTPaseObg | 2 (1,2) |  |  |  |
| BAQ32838 | *nusG* | transcription antitermination protein | 3 (3,2,2) |  |  |  |
| BAQ32840 | *murA* | UDP-N-acetylglucosamine 1-carboxyvinyltransferase | 2 (2,1) |  |  | 1 |
| BAQ32844 | *rpsO* | 30S ribosomal protein S15 | 3 (2,1,1) |  |  |  |
| BAQ32845 | *pnp* | polynucleotide phosphorylase/polyadenylase | 3 (5,5,4) |  |  | 1 |
| BAQ32846 |  | putative oxidoreductase | 3 (3,1,3) |  |  |  |
| BAQ32849 |  | conserved hypothetical protein | 3 (15,12,7) |  |  |  |
| BAQ32850 |  | aminopeptidase | 2 (1,2) |  |  |  |
| BAQ32853 | *infB* | translation initiation factor IF-2 | 3 (10,6,6) |  |  |  |
| BAQ32856 |  | riboflavin kinase | 2 (2,2) |  |  |  |
| BAQ32865 | *rplJ* | 50S ribosomal protein L10 | 3 (6,6,6) |  |  |  |
| BAQ32866 | *rplL** | 50S ribosomal protein L7/L12 | 3 (4,4,4) |  |  |  |
| BAQ32867 | *rplK** | 50S ribosomal protein L11 | 3 (3,2,1) |  |  |  |
| BAQ32868 | *rplA* | 50S ribosomal protein L1 | 3 (9,7,6) |  |  |  |
| BAQ32876 |  | putative sugar ABC transporter substrate binding component | 3 (1,1,2) | yes | lipobox | 1 |
| BAQ32877 |  | putative sugar ABC transporter ATP-binding component | 3 (9,5,2) |  |  |  |
| BAQ32880 |  | putative carbohydrate kinase | 2 (2,1) |  |  |  |
| BAQ32883 |  | putative reductase | 3 (7,3,2) |  |  |  |
| BAQ32894 | *rpiA* | ribose-5-phosphate isomerase A | 2 (2,2) |  |  |  |
| BAQ32896 |  | phosphoglucomutase | 2 (2,1) |  |  |  |
| BAQ32898 |  | conserved hypothetical protein | 3 (4,5,5) |  |  | 1 |
| BAQ32907 |  | glucose-1-phosphate thymidylyltransferase | 2 (2,1) |  |  | 1 |
| BAQ32908 |  | dTDP-4-dehydrorhamnose reductase/dTDP-4-keto-6-deoxyglucose-3,5-epimerase | 3 (1,1,2) |  |  |  |
| BAQ32912 |  | putative NAD-dependent epimerase/dehydratase | 3 (2,3,2) |  |  |  |
| BAQ32921 | *serS* | seryl-tRNA ligase | 3 (11,11,6) |  |  |  |
| BAQ32922 |  | thioredoxin | 3 (3,2,1) |  |  |  |
| BAQ32935 | *glmM* | phosphoglucosamine mutase | 3 (3,3,2) |  |  | 1 |
| BAQ32936 | *def* | peptide deformylase | 3 (1,1,2) |  |  | 1 |
| BAQ32945 |  | hypoxanthine-guanine phosphoribosyltransferase | 3 (12,7,5) |  |  | 1 |
| BAQ32949 |  | ABC transporter ATP-binding component | 3 (7,2,2) |  |  |  |
| BAQ32957 |  | hypothetical protein | 3 (2,2,4) | yes | lipobox |  |
| BAQ32960 |  | peptidyl-prolyl cis-trans isomerase | 3 (2,2,2) |  |  |  |
| BAQ32961 | *greA* | transcription elongation factor GreA | 3 (8,7,8) |  |  |  |
| BAQ32970 |  | penicillin-binding protein | 3 (6,2,8) |  |  | 1 |
| BAQ32974 | *murD* | UDP-N-acetylmuramoylalanine-D-glutamate ligase | 3 (2,1,1) |  |  | 1 |
| BAQ32977 | *murC* | UDP-N-acetylmuramate-L-alanine ligase | 3 (3,1,1) |  |  |  |
| BAQ32980 | *rpsG* | 30S ribosomal protein S7 | 3 (11,9,6) |  |  |  |
| BAQ32981 | *ef-G* * | elongation factor G (FusA) | 3 (17,14,16) |  |  |  |
| BAQ32982 | *ef-Tu** | elongation factor Tu | 3 (4,3,3) |  |  |  |
| BAQ32987 | *ileS* | isoleucyl-tRNA ligase | 3 (2,1,3) |  |  |  |
| BAQ32990 |  | putative ABC transporter substrate binding component | 3 (5,3,9) |  | lipobox | 1 |
| BAQ33005 |  | conserved hypothetical protein | 3 (4,3,2) |  |  |  |
| BAQ33013 | *glyQS* | glycyl-tRNA ligase | 3 (8,6,4) |  |  |  |
| BAQ33015 | *ftsZ* | cell division protein FtsZ | 3 (10,6,5) |  |  |  |
| BAQ33018 |  | conserved hypothetical protein | 3 (12,12,7) |  |  |  |
| BAQ33037 | *lysS* | lysyl-tRNA ligase | 3 (7,6,4) |  |  |  |
| BAQ33038 | *gpmA** | phosphoglycerate mutase | 3 (2,1,2) |  |  |  |
| BAQ33039 | *phoU* | phosphate-specific transport system accessory protein | 3 (8,5,1) |  |  |  |
| BAQ33044 |  | conserved hypothetical protein | 2 (5,3) |  |  |  |
| BAQ33047 | *thyA* | thymidylate synthase | 3 (2,3,3) |  |  |  |
| BAQ33049 |  | conserved hypothetical protein | 3 (2,1,2) |  |  |  |
| BAQ33051 |  | conserved hypothetical protein | 3 (2,1,1) |  |  | 2 |
| BAQ33052 |  | conserved hypothetical protein | 3 (16,11,10) |  |  |  |
| BAQ33061 |  | conserved hypothetical protein | 3 (6,6,6) |  |  |  |
| BAQ33074 | *ackA* | acetate kinase | 3 (15,14,14) |  |  |  |
| BAQ33075 |  | phosphate acetyltransferase | 3 (8,7,6) |  |  | 2 |
| BAQ33076 |  | xylulose-5-phosphate/fructose-6-phosphate phosphoketolase | 3 (26,16,27) |  |  |  |
| BAQ33077 | *guaA* | bifunctional GMP synthase and glutamine amidotransferase protein | 3 (5,3,3) |  |  | 1 |
| BAQ33083 | *pheS* | phenylalanyl-tRNA ligase alpha subunit | 3 (11,7,8) |  |  |  |
| BAQ33084 | *pheT* | phenylalanyl-tRNA ligase beta subunit | 3 (12,6,12) |  |  |  |
| BAQ33088 | *tyrS* | tyrosyl-tRNA ligase | 2 (3,2) |  |  |  |
| BAQ33095 |  | conserved hypothetical protein | 3 (6,4,2) |  |  |  |
| BAQ33096 | *secA* * | preprotein translocase subunit | 2 (4,1) |  |  |  |
| BAQ33102 | *sigA* | RNA polymerase sigma factor | 2 (4,1) |  |  |  |
| BAQ33117 |  | conserved hypothetical protein | 3 (2,1,2) |  |  |  |
| BAQ33121 |  | peptidyl-prolyl cis-trans isomerase | 3 (7,6,7) |  |  |  |
| BAQ33132 | *tig* | trigger factor | 3 (10,6,4) |  |  |  |
| BAQ33134 | *clpP* | protease Clp proteolytic subunit | 3 (3,2,2) |  |  | 1 |
| BAQ33136 |  | DNA-binding protein | 3 (4,4,3) |  |  |  |
| BAQ33138 |  | adenylosuccinatelyase | 3 (2,2,1) |  |  |  |
| BAQ33149 | *groEL** | chaperonin GroEL | 3 (9,5,7) |  |  |  |
| BAQ33152 |  | two-component response regulator | 3 (4,2,1) |  |  |  |
| BAQ33154 |  | cold shock protein | 3 (1,2,1) |  |  |  |
| BAQ33155 |  | conserved hypothetical protein | 2 (2,1) |  |  |  |
| BAQ33156 | *clp-ATP* | ATP-dependent Clp protease ATP-binding subunit | 3 (20,19,12) |  |  |  |
| BAQ33195 | *ychF* | Ribosome-binding ATPase | 3 (4,4,6) |  |  |  |
| BAQ33200 |  | conserved hypothetical protein | 3 (3,1,2) | yes |  | 7 |
| BAQ33208 | *eno** | enolase | 2 (2,1) |  |  | 1 |
| BAQ33209 | *ldh* | L-lactate dehydrogenase | 3 (3,2,2) | yes |  |  |
| BAQ33210 |  | conserved hypothetical protein | 2 (3,2) |  |  | 1 |
| BAQ33222 | *leuS* | leucyl-tRNA ligase | 3 (7,2,8) |  | lipobox |  |
| BAQ33226 |  | conserved hypothetical protein | 2 (3,2) |  |  |  |
| BAQ33240 | *pyrE* | orotatephosphoribosyltransferase | 2 (3,3) |  |  |  |
| BAQ33242 | *ef-P* | elongation factor P | 3 (2,3,2) |  |  |  |
| BAQ33244 | *carA* | carbamoyl phosphate synthase small subunit | 3 (4,1,2) |  |  |  |
| BAQ33245 | *carB* | carbamoyl phosphate synthase large subunit | 3 (8,6,2) |  |  |  |
| BAQ33247 | *gmk* | guanylate kinase | 2 (3,1) |  |  |  |
| BAQ33248 | *rpoZ* | DNA-directed RNA polymerase omega subunit | 3 (2,2,1) |  |  |  |
| BAQ33257 |  | conserved hypothetical protein | 3 (2,2,1) |  |  |  |
| BAQ33273 | *tal* | transaldolase | 3 (4,3,8) |  |  |  |
| BAQ33274 |  | transketolase | 3 (20,15,16) |  |  |  |
| BAQ33277 | *fmt* | methionyl-tRNAformyltransferase | 3 (3,2,1) |  |  |  |
| BAQ33280 |  | conserved hypothetical protein | 3 (5,5,3) |  |  | 1 |
| BAQ33288 |  | long-chain-fatty acid CoA ligase | 3 (4,3,3) |  | lipobox | 1 |
| BAQ33290 | *rpsB* | 30S ribosomal protein S2 | 3 (4,2,5) |  |  |  |
| BAQ33291 | *tsf** | elongation factor Ts | 3 (9,8,12) |  |  |  |
| BAQ33292 | *pyrH* | uridylate kinase | 3 (4,3,6) |  |  |  |
| BAQ33293 | *frr* | ribosome recycling factor | 3 (9,8,9) |  |  |  |
| BAQ33303 |  | truncated hydrolase | 2 (2,2) |  | lipobox |  |
| BAQ33304 | *rbpA* | RNA polymerase-binding protein | 3 (2,1,1) |  |  |  |
| BAQ33307 |  | conserved hypothetical protein | 2 (3,2) |  |  |  |
| BAQ33313 | *aspS* | aspartyl-tRNAsinthetase | 3 (5,2,2) |  |  |  |
| BAQ33314 | *hisS* | histidyl-tRNA ligase | 3 (10,6,3) |  |  |  |
| BAQ33315 |  | conserved hypothetical protein | 3 (17,8,15) |  |  |  |
| BAQ33322 |  | dehydrogenase | 3 (13,12,11) |  |  |  |
| BAQ33340 |  | conserved hypothetical protein | 3 (3,2,3) |  |  |  |
| BAQ33345 | *proA* | gamma-glutamyl phosphate reductase | 3 (2,1,2) |  |  |  |
| BAQ33357 | *purM* | phosphoribosylformylglycinamidinecyclo-ligase | 3 (3,4,2) |  |  |  |
| BAQ33363 | *purK* | phosphoribosylaminoimidazole carboxylase ATPase subunit | 2 (3,1) | yes |  |  |
| BAQ33365 |  | ABC transporter ATP-binding component | 3 (4,1,1) |  |  | 1 |
| BAQ33368 |  | conserved hypothetical protein | 2 (3,3) | yes |  | 2 |
| BAQ33371 | *rpsT* | 30S ribosomal protein S20 | 3 (1,1,3) |  |  |  |
| BAQ33399 | *pdxT* | pyridoxal 5´-phosphate synthase subunit | 2 (4,1) |  |  | 1 |
| BAQ33408 |  | conserved hypothetical protein | 3 (6,6,8) | yes | lipobox | 1 |
| BAQ33410 | *ftsE* | cell division ATP-binding protein FtsE | 3 (2,1,1) |  |  |  |
| BAQ33411 | *prfB* | peptide chain release factor 2 | 3 (6,3,4) |  |  |  |
| BAQ33412 | *map* | methionine aminopeptidase | 3 (2,2,2) |  |  |  |
| BAQ33413 |  | metalloendopeptidase | 3 (4,3,4) |  |  | 1 |
| BAQ33416 | *proS* | prolyl-tRNA ligase | 3 (7,6,7) |  |  |  |
| BAQ33418 |  | oligoribonuclease | 3 (2,2,2) |  |  |  |
| BAQ33421 | *impdh** | inosine-5'-monophosphate dehydrogenase (GuaB) | 3 (4,2,3) |  | lipobox |  |
| BAQ33425 | *prfA* | peptide chain release factor 1 | 2 (5,3) |  |  |  |
| BAQ33427 |  | putative cell surface protein | 3 (16,12,17) | yes | LPXTG | 2 |
| BAQ33428 | *rplY* | 50S ribosomal protein L25 | 3 (13,11,8) |  |  |  |
| BAQ33431 |  | hypothetical protein | 3 (31,28,39) | yes |  | 1 |
| BAQ33436 |  | conserved hypothetical protein | 3 (3,3,2) |  |  |  |
| BAQ33444 |  | conserved hypothetical protein | 3 (4,4,5) |  |  |  |
| BAQ33450 | *pyK** | pyruvate kinase | 3 (11,11,13) |  |  |  |
| BAQ33456 |  | 30S ribosomal protein S1 | 3 (7,4,8) |  |  | 1 |
| BAQ33457 | *folD* | bifunctional methylenetetrahydrofolate dehydrogenase and methenyltetrahydrofolatecyclohydrolase | 3 (2,1,2) |  |  |  |
| BAQ33477 |  | NADH oxidase | 3 (2,1,1) |  | lipobox |  |
| BAQ33480 | *yhgE* | YhgE/Pip N-terminal domain-containing protein | 2 (2,1) |  |  | 6 |
| BAQ33494 | *cysK* | cysteine synthase | 3 (10,5,6) |  |  |  |
| BAQ33535 |  | conserved hypothetical protein | 3 (5,4,1) |  |  |  |
| BAQ33541 | *pyrG* | CTP synthase | 3 (10,6,6) |  |  |  |
| BAQ33544 | *aroC* | chorismate synthase | 3 (7,4,3) |  |  |  |
| BAQ33548 | *alaS* | alanyl-tRNA ligase | 3 (20,13,12) |  |  | 1 |
| BAQ33552 |  | galactose-1-phosphate uridylyltransferase | 3 (3,2,2) |  |  |  |
| BAQ33553 | *rpsD* | 30S ribosomal protein S4 | 3 (4,3,6) |  |  |  |
| BAQ33576 |  | conserved hypothetical protein | 3 (7,5,6) |  |  | 1 |
| BAQ33577 |  | ABC transporter ATP-binding component | 3 (10,6,4) |  |  |  |
| BAQ33579 |  | ABC transporter permease component | 2 (11,4) |  |  |  |
| BAQ33586 |  | conserved hypothetical protein | 3 (8,7,4) |  |  |  |
| BAQ33590 | *cysS* | cysteinyl-tRNA ligase | 2 (4,1) |  |  |  |
| BAQ33593 | *rpsP* | 30S ribosomal protein S16 | 3 (3,3,2) |  |  |  |
| BAQ33594 |  | conserved hypothetical protein | 3 (2,1,1) |  |  |  |
| BAQ33600 |  | conserved hypothetical protein | 2 (3,1) |  |  | 1 |
| BAQ33602 | *rpmB* | 50S ribosomal protein L28 | 3 (2,2,1) |  |  |  |
| BAQ33606 |  | conserved hypothetical protein | 3 (12,11,8) | yes | LPXTG | 2 |
| BAQ33607 | *rplI* | 50S ribosomal protein L9 | 3 (3,2,1) |  |  |  |
| BAQ33608 | *rpsR* | 30S ribosomal protein S18 | 3 (8,7,5) |  |  |  |
| BAQ33610 | *rpsF* | 30S ribosomal protein S6 | 3 (7,6,5) |  |  |  |
| BAQ33615 | *rplT* | 50S ribosomal protein L20 | 3 (6,3,3) |  |  |  |
| BAQ33616 | *rpmI* | 50S ribosomal protein L35 | 3 (2,1,2) |  |  |  |
| BAQ33619 | *gap* * | glyceraldehyde-3-phosphate dehydrogenase | 3 (3,2,4) |  |  |  |
| BAQ33620 |  | conserved hypothetical protein | 3 (4,4,2) |  |  |  |
| BAQ33625 | *galE* | UDP-glucose 4-epimerase | 3 (12,5,5) |  |  |  |
| BAQ33632 |  | galactokinase | 3 (9,8,3) |  |  |  |
| BAQ33633 |  | galactose-1-phosphate uridylyltransferase | 3 (5,5,6) |  |  |  |
| BAQ33639 | *pepP* | Xaa-Pro aminopeptidase | 3 (8,7,6) |  |  |  |
| BAQ33644 |  | hypothetical protein | 3 (30,28,19) | yes |  | 1 |
| BAQ33652 |  | cell wall associated fibronectin-binding protein | 3 (2,1,3) | yes | LPXTG | 1 |
| BAQ33657 | *rpoC* | DNA-directed RNA polymerase beta' subunit | 3 (13,11,2) |  |  |  |
| BAQ33658 | *rpoB* | DNA-directed RNA polymerase beta subunit | 3 (8,3,1) |  |  |  |
| BAQ33666 | *rplS* * | 50S ribosomal protein L19 | 3 (5,3,2) |  |  |  |
| BAQ33667 |  | nitrate ABC transporter ATP-binding component | 3 (7,5,3) |  |  | 1 |
| BAQ33672 |  | putative cell surface protein | 3 (5,6,8) | yes | LPXTG | 1 |
| BAQ33673 |  | amylopullulanase | 3 (4,2,5) | yes |  | 1 |
| BAQ33676 |  | conserved hypothetical protein | 2 (2,1) |  |  |  |
| BAQ33706 | *rpsK* | 30S ribosomal protein S11 | 3 (3,2,1) |  |  |  |
| BAQ33707 | *rpsM* | 30S ribosomal protein S13 | 3 (8,7,8) |  |  |  |
| BAQ33709 | *infA* | translation initiation factor IF-1 | 3 (3,3,2) |  |  |  |
| BAQ33710 | *adk* | adenylate kinase | 3 (9,7,6) |  |  |  |
| BAQ33712 | *rplO* | 50S ribosomal protein L15 | 2 (1,2) |  |  |  |
| BAQ33713 | *rpmD* | 50S ribosomal protein L30 | 3 (5,5,5) |  |  |  |
| BAQ33714 | *rpsE* | 30S ribosomal protein S5 | 3 (4,3,3) |  |  |  |
| BAQ33715 | *rplR* | 50S ribosomal protein L18 | 3 (4,4,4) |  |  |  |
| BAQ33716 | *rplF* | 50S ribosomal protein L6 | 3 (9,7,7) |  |  |  |
| BAQ33717 | *rpsH** | 30S ribosomal protein S8 | 3 (6,2,3) |  |  |  |
| BAQ33719 | *rplE** | 50S ribosomal protein L5 | 3 (9,9,11) |  |  |  |
| BAQ33720 | *rplX* | 50S ribosomal protein L24 | 3 (6,3,4) |  |  |  |
| BAQ33721 | *rplN* | 50S ribosomal protein L14 | 3 (1,1,2) |  |  |  |
| BAQ33722 | *rpsQ* | 30S ribosomal protein S17 | 3 (4,3,2) | yes |  |  |
| BAQ33723 | *rpmC* | 50S ribosomal protein L29 | 3 (12,11,7) |  |  |  |
| BAQ33724 | *rplP* | 50S ribosomal protein L16 | 3 (2,1,2) | yes |  |  |
| BAQ33725 | *rpsC* | 30S ribosomal protein S3 | 3 (4,2,5) |  |  |  |
| BAQ33726 | *rplV* | 50S ribosomal protein L22 | 3 (11,9,7) |  |  |  |
| BAQ33727 | *rpsS* | 30S ribosomal protein S19 | 3 (7,5,5) |  |  |  |
| BAQ33728 | *rplB* | 50S ribosomal protein | 3 (12,8,10) |  |  |  |
| BAQ33729 | *rplW* | 50S ribosomal protein L23 | 3 (6,3,3) |  |  |  |
| BAQ33730 | *rplD* | 50S ribosomal protein L4 | 3 (7,5,4) |  |  |  |
| BAQ33732 | *rpsJ* | 30S ribosomal protein S10 | 3 (7,7,5) |  |  |  |
| BAQ33734 | *rpsI* * | 30S ribosomal protein S9 | 3 (4,3,3) |  |  |  |
| BAQ33735 | *rplM* | 50S ribosomal protein L13 | 3 (4,4,2) |  |  |  |
| BAQ33737 |  | conserved hypothetical protein | 2 (4,3) |  |  |  |
| BAQ33746 | *rpmG* | 50S ribosomal protein L33 | 3 (2,1,1) |  |  |  |
| BAQ33748 | *groES* | chaperone GroES | 3 (7,6,4) |  |  |  |
| BAQ33754 |  | conserved hypothetical protein | 2 (4,2) |  |  |  |
| BAQ33755 | *gltX* | glutamyl-tRNA ligase | 2 (2,1) |  |  |  |
| BAQ33756 |  | conserved hypothetical protein | 3 (3,1,2) |  |  |  |
| BAQ33766 | *argS* | arginyl-tRNA ligase | 3 (3,3,1) |  |  |  |
| BAQ33774 | *zwf* | glucose-6-phosphate 1-dehydrogenase | 2 (2,1) |  |  |  |
| BAQ33778 |  | 6-phosphogluconate dehydrogenase | 3 (5,5,4) | yes |  | 1 |
| BAQ33781 |  | ribonucleotide-diphosphate reductase alpha subunit | 3 (2,1,4) |  |  |  |
| BAQ33782 |  | ribonucleoside-diphosphate reductase beta subunit | 3 (3,2,3) |  |  | 1 |
| BAQ33805 |  | high-affinity Fe2+/Pb2+ permease | 3 (1,1,2) | yes | lipobox | 7 |
| BAQ33816 |  | hypothetical protein | 3 (11,11,11) | yes |  | 1 |
| BAQ33818 | *atpD* | ATP synthase beta subunit | 3 (15,14,11) |  |  |  |
| BAQ33820 | *atpA* | ATP synthase alpha subunit | 3 (4,3,3) |  | lipobox |  |
| BAQ33822 | *atpF* | ATP synthase subunit B | 3 (2,3,2) | yes |  | 1 |
| BAQ33864 |  | transcriptional regulator | 2 (2,2) |  |  |  |
| BAQ33865 | *rho* | transcription termination factor | 3 (13,9,4) |  |  | 1 |
| BAQ33868 |  | putative acetyltransferase | 2 (4,2) |  |  |  |
| BAQ33869 | *gatB* | aspartyl/glutamyl-tRNAamidotransferase subunit B | 3 (9,8,7) |  |  |  |
| BAQ33870 | *gatA* | aspartyl/glutamyl-tRNAamidotransferase subunit A | 3 (9,5,6) |  |  |  |
| BAQ33882 | *clpB* | chaperone ClpB | 3 (6,5,3) |  |  | 1 |
| BAQ33883 |  | 2-hydroxyhepta-2,4-diene-1,7-dioate isomerase | 2 (2,1) |  |  | 1 |
| BAQ33887 | *ppk* | polyphosphate kinase | 3 (6,6,1) |  |  |  |
| BAQ33893 | *upp* | uracil phosphoribosyltransferase | 3 (7,5,8) |  |  |  |
| BAQ33899 | *ftsY* | putative signal recognition particle-docking protein FtsY | 3 (6,3,2) | yes |  | 1 |
| BAQ33910 |  | ABC transporter ATP-binding component | 3 (11,9,8) |  |  |  |
| BAQ33912 |  | conserved hypothetical protein | 2 (2,1) |  |  | 1 |
| BAQ33922 | *parB* | chromosome partitioning protein ParB | 2 (2,1) | yes |  |  |
| BAQ33925 |  | conserved hypothetical protein | 3 (4,4,1) |  |  |  |

a) Protein ID, gene name and description from Genome Project of *G. vaginalis* JCM 11026 (Oshima *et al.*, 2015), listed in numerical order of protein ID

(<http://www.ncbi.nlm.nih.gov/Taxonomy/Browser/wwwtax.cgi?id=585528>).

b) Proteins were included if they were identified in at least two replicates with at least two peptides in one replicate. Number of unique peptides identified in each replicate is indicated.

c) Signal peptide (SP) prediction: by SignalP 4.1 server and PSORT server of Sec secretion pathway (Petersen *et al.*, 2011), by TatP 1.0 server for Tat secretion pathway and by PRED-LIPO server for lipo signal peptide.

d) Proteins predicted to be anchored to the cell-membrane or cell-wall via different domains or motifs: **LPxTG**, proteins with a C-terminal LPxTG cell wall anchoring signal for covalent attachment to peptidoglycan by sortase or **Lipobox**, lipoproteins with an N-terminal lipobox that mediates the covalent binding of a conserved cysteine residue to a lipid on the cell wall.

e) Number of transmembrane alpha-helix (TMD) indicated was predicted with PSORT server. Additionally with TMHMM Server version 2.0 was predicted TMD for 6 additional proteins (BAQ32696, BAQ32762, BAQ32803, BAQ32876, BAQ32970 and BAQ33899).

Proteins indicated with * are considered as moonlighting or at least with dual localization in other Gram-positive microorganisms (cytoplasm and bacterial surface) (Dallo *et al.*, 2002;Bendtsen *et al.*, 2005;Sellman *et al.*, 2005;Severin *et al.*, 2007;Henderson and Martin, 2011;Kainulainen and Korhonen, 2014;Wang *et al.*, 2014;Espino *et al.*, 2015).

**Bibliography**

Bendtsen, J.D., Kiemer, L., Fausboll, A., and Brunak, S. (2005). Non-classical protein secretion in bacteria. *BMC Microbiol* 5**,** 58. doi: 10.1186/1471-2180-5-58.

Dallo, S.F., Kannan, T.R., Blaylock, M.W., and Baseman, J.B. (2002). Elongation factor Tu and E1 beta subunit of pyruvate dehydrogenase complex act as fibronectin binding proteins in *Mycoplasma pneumoniae*. *Mol Microbiol* 46**,** 1041-1051. doi: 10.1046/j.1365-2958.2002.03207.x.

Espino, E., Koskenniemi, K., Mato-Rodriguez, L., Nyman, T.A., Reunanen, J., Koponen, J., Ohman, T., Siljamaki, P., Alatossava, T., Varmanen, P., and Savijoki, K. (2015). Uncovering surface-exposed antigens of *Lactobacillus rhamnosus* by cell shaving proteomics and two-dimensional immunoblotting. *J Proteome Res* 14**,** 1010-1024. doi: 10.1021/pr501041a.

Henderson, B., and Martin, A. (2011). Bacterial virulence in the moonlight: multitasking bacterial moonlighting proteins are virulence determinants in infectious disease. *Infect Immun* 79**,** 3476-3491. doi: 10.1128/IAI.00179-11.

Kainulainen, V., and Korhonen, T.K. (2014). Dancing to another tune-adhesive moonlighting proteins in bacteria. *Biology (Basel)* 3**,** 178-204. doi: 10.3390/biology3010178.

Oshima, K., Hisamatsu, S., Toh, H., Nakano, A., Kiuchi, M., Kuroyanagi, H., Morita, H., and Hattori, M. (2015). Complete Genome Sequence of *Gardnerella vaginalis* Strain JCM 11026T, Isolated from Vaginal Tracts of Women. *Genome Announc* 3. doi: 10.1128/genomeA.00286-15.

Petersen, T.N., Brunak, S., Von Heijne, G., and Nielsen, H. (2011). SignalP 4.0: discriminating signal peptides from transmembrane regions. *Nat Methods* 8**,** 785-786. doi: 10.1038/nmeth.1701.

Sellman, B.R., Howell, A.P., Kelly-Boyd, C., and Baker, S.M. (2005). Identification of immunogenic and serum binding proteins of Staphylococcus epidermidis. *Infect Immun* 73**,** 6591-6600. doi: 10.1128/IAI.73.10.6591-6600.2005.

Severin, A., Nickbarg, E., Wooters, J., Quazi, S.A., Matsuka, Y.V., Murphy, E., Moutsatsos, I.K., Zagursky, R.J., and Olmsted, S.B. (2007). Proteomic analysis and identification of Streptococcus pyogenes surface-associated proteins. *J Bacteriol* 189**,** 1514-1522. doi: 10.1128/JB.01132-06.

Wang, G., Xia, Y., Cui, J., Gu, Z., Song, Y., Chen, Y.Q., Chen, H., Zhang, H., and Chen, W. (2014). The roles of moonlighting proteins in bacteria. *Curr Issues Mol Biol* 16**,** 15-22.
