# Supplementary material for: Unraveling Gardnerella vaginalis Surface Proteins Using Cell Shaving Proteomics
Source: Front Microbiol. 2018 May 15;9:975. doi: 10.3389/fmicb.2018.00975 (PMC5962675; doi:10.3389/fmicb.2018.00975)
Supplement: Supplementary file 2 [file Table_2.DOCX]

**Table S2. Subcellular classification of *G. vaginalis* identified proteins using the PSORT and Gpos-mPLoc servers.**

| Protein_ID^a^ | Description^a^ | Subcellular localization PSORT^b^ | Subcellular localization Gpos-mPLoc^c^ | Classification^d^ |
| --- | --- | --- | --- | --- |
| BAQ32657 | DNA gyrase subunit B | cytoplasm | Cell membrane/cytoplasm | both |
| BAQ32662 | conserved hypothetical protein | cytoplasm | Cell membrane | both |
| BAQ32664 | putative phosphatase | plasmatic membrane | Cytoplasm | both |
| BAQ32680 | phosphoenolpyruvate carboxylase | plasmatic membrane | Cell membrane | surface associated |
| BAQ32685 | conserved hypothetical protein | cytoplasm | Cytoplasm | inside |
| BAQ32687 | ferredoxin/ferredoxin-NADP reductase | cytoplasm | Cytoplasm | inside |
| BAQ32694 | hypothetical protein | plasmatic membrane | Extracellular | surface associated |
| BAQ32696 | conserved hypothetical protein | outside | Extracellular | surface associated |
| BAQ32710 | serine protease | plasmatic membrane | Extracellular | surface associated |
| BAQ32713 | RNA methyltransferase | cytoplasm | Cell membrane/cytoplasm | both |
| BAQ32724 | 2-dehydropantoate 2-reductase | cytoplasm | Cytoplasm | inside |
| BAQ32758 | M protein repeat protein | plasmatic membrane | cell membrane | surface associated |
| BAQ32759 | pullulanase precursor | plasmatic membrane | extracellular | surface associated |
| BAQ32762 | putative ABC transporter substrate binding component | lipoprotein | Extracellular | surface associated |
| BAQ32771 | conserved hypothetical protein | plasmatic membrane | cell membrane | surface associated |
| BAQ32776 | chaperone protein DnaK * | cytoplasm | cytoplasm | inside |
| BAQ32777 | chaperone protein GrpE | cytoplasm | cytoplasm | inside |
| BAQ32780 | conserved hypothetical protein | cytoplasm | Cell membrane/cytoplasm | both |
| BAQ32781 | putative penicillin-binding protein | plasmatic membrane | cell membrane | surface associated |
| BAQ32789 | conserved hypothetical protein | cytoplasm | Cell membrane/cytoplasm | both |
| BAQ32791 | formate-tetrahydrofolate ligase | plasmatic membrane | cytoplasm | both |
| BAQ32792 | Cna protein B-type domain-containing protein | plasmatic membrane | Extracellular | surface associated |
| BAQ32799 | aminopeptidase C | cytoplasm | cytoplasm | inside |
| BAQ32802 | dipeptide/oligopeptide ABC transporter ATP-binding component | plasmatic membrane | cell membrane | surface associated |
| BAQ32803 | dipeptide/oligopeptide ABC transporter substrate binding component | lipoprotein | cell membrane | surface associated |
| BAQ32810 | carbohydrate kinase | outside | cytoplasm | both |
| BAQ32812 | 6-phosphogluconate dehydrogenase-like protein | cytoplasm | cytoplasm | inside |
| BAQ32815 | hypothetical protein | plasmatic membrane | Extracellular | surface associated |
| BAQ32817 | hypothetical protein | plasmatic membrane | Extracellular | surface associated |
| BAQ32818 | putative ABC transporter substrate binding component | lipoprotein | cell membrane | surface associated |
| BAQ32822 | glycerol-3-phosphate dehydrogenase | plasmatic membrane | cytoplasm | both |
| BAQ32823 | D-alanine-D-alanine ligase | cytoplasm | cell membrane | both |
| BAQ32826 | homoserine dehydrogenase | cytoplasm | cytoplasm | inside |
| BAQ32829 | hypothetical protein | plasmatic membrane | Extracellular | surface associated |
| BAQ32831 | ribonuclease G | cytoplasm | Cytoplasm/extracellular | both |
| BAQ32832 | 50S ribosomal protein L21 | cytoplasm | cytoplasm | inside |
| BAQ32833 | 50S ribosomal protein L27 | cytoplasm | Extracellular | both |
| BAQ32834 | GTPase Obg | cytoplasm | cytoplasm | inside |
| BAQ32838 | transcription antitermination protein | cytoplasm | cytoplasm | inside |
| BAQ32840 | UDP-N-acetylglucosamine 1-carboxyvinyltransferase | plasmatic membrane | cytoplasm | both |
| BAQ32844 | 30S ribosomal protein S15 | cytoplasm | cytoplasm | inside |
| BAQ32845 | polynucleotide phosphorylase/polyadenylase | plasmatic membrane | cytoplasm | both |
| BAQ32846 | putative oxidoreductase | cytoplasm | cytoplasm | inside |
| BAQ32849 | conserved hypothetical protein | cytoplasm | cell membrane | both |
| BAQ32850 | aminopeptidase | cytoplasm | cytoplasm | inside |
| BAQ32853 | translation initiation factor IF-2 | cytoplasm | cytoplasm | inside |
| BAQ32856 | riboflavin kinase | cytoplasm | cytoplasm | inside |
| BAQ32865 | 50S ribosomal protein L10 | cytoplasm | cytoplasm | inside |
| BAQ32866 | 50S ribosomal protein L7/L12 * (*rplL*) | cytoplasm | cytoplasm | inside |
| BAQ32867 | 50S ribosomal protein L11 * (*rplK*) | cytoplasm | cytoplasm | inside |
| BAQ32868 | 50S ribosomal protein L1 | cytoplasm | cytoplasm | inside |
| BAQ32876 | putative sugar ABC transporter substrate binding component | lipoprotein | cytoplasm | both |
| BAQ32877 | putative sugar ABC transporter ATP-binding component | cytoplasm | cell membrane | both |
| BAQ32880 | putative carbohydrate kinase | cytoplasm | cytoplasm | inside |
| BAQ32883 | putative reductase | cytoplasm | cytoplasm | inside |
| BAQ32894 | ribose-5-phosphate isomerase A | cytoplasm | cytoplasm | inside |
| BAQ32896 | phosphoglucomutase | cytoplasm | cytoplasm | inside |
| BAQ32898 | conserved hypothetical protein | plasmatic membrane | Extracellular | surface associated |
| BAQ32907 | glucose-1-phosphate thymidylyltransferase | plasmatic membrane | cytoplasm | both |
| BAQ32908 | dTDP-4-dehydrorhamnose reductase/dTDP-4-keto-6-deoxyglucose-3,5-epimerase | unknown | cytoplasm | inside |
| BAQ32912 | putative NAD-dependent epimerase/dehydratase | cytoplasm | cytoplasm | inside |
| BAQ32921 | seryl-tRNA ligase | cytoplasm | cytoplasm | inside |
| BAQ32922 | thioredoxin | cytoplasm | Extracellular | both |
| BAQ32935 | phosphoglucosamine mutase | plasmatic membrane | cytoplasm | both |
| BAQ32936 | peptide deformylase | plasmatic membrane | cytoplasm | both |
| BAQ32945 | hypoxanthine-guanine phosphoribosyltransferase | plasmatic membrane | cytoplasm | both |
| BAQ32949 | ABC transporter ATP-binding component | cytoplasm | cell membrane | both |
| BAQ32957 | hypothetical protein | outside | Extracellular | surface associated |
| BAQ32960 | peptidyl-prolyl cis-trans isomerase | cytoplasm | cytoplasm | inside |
| BAQ32961 | transcription elongation factor GreA | cytoplasm | cytoplasm | inside |
| BAQ32970 | penicillin-binding protein | plasmatic membrane | cell membrane | surface associated |
| BAQ32974 | UDP-N-acetylmuramoylalanine-D-glutamate ligase | plasmatic membrane | cell membrane | surface associated |
| BAQ32977 | UDP-N-acetylmuramate-L-alanine ligase | cytoplasm | Cell membrane/cytoplasm | both |
| BAQ32980 | 30S ribosomal protein S7 | cytoplasm | cytoplasm | inside |
| BAQ32981 | elongation factor G (FusA) * (*ef-G*) | cytoplasm | cytoplasm | inside |
| BAQ32982 | elongation factor Tu * (*ef-Tu*) | cytoplasm | cytoplasm | inside |
| BAQ32987 | isoleucyl-tRNA ligase | cytoplasm | cytoplasm | inside |
| BAQ32990 | putative ABC transporter substrate binding component | plasmatic membrane | cell membrane | surface associated |
| BAQ33005 | conserved hypothetical protein | cytoplasm | Extracellular | both |
| BAQ33013 | glycyl-tRNA ligase | cytoplasm | cytoplasm | inside |
| BAQ33015 | cell division protein FtsZ | cytoplasm | cytoplasm | inside |
| BAQ33018 | conserved hypothetical protein | cytoplasm | cell membrane | both |
| BAQ33037 | lysyl-tRNA ligase | cytoplasm | cytoplasm | inside |
| BAQ33038 | phosphoglycerate mutase * (*gpmA*) | cytoplasm | cytoplasm | inside |
| BAQ33039 | phosphate-specific transport system accessory protein | cytoplasm | cytoplasm | inside |
| BAQ33044 | conserved hypothetical protein | cytoplasm | cytoplasm | inside |
| BAQ33047 | thymidylate synthase | cytoplasm | cytoplasm | inside |
| BAQ33049 | conserved hypothetical protein | cytoplasm | cytoplasm | inside |
| BAQ33051 | conserved hypothetical protein | plasmatic membrane | Extracellular | surface associated |
| BAQ33052 | conserved hypothetical protein | cytoplasm | cytoplasm | inside |
| BAQ33061 | conserved hypothetical protein | cytoplasm | cytoplasm | inside |
| BAQ33074 | acetate kinase | cytoplasm | cytoplasm | inside |
| BAQ33075 | phosphate acetyltransferase | plasmatic membrane | cytoplasm | both |
| BAQ33076 | xylulose-5-phosphate/fructose-6-phosphate phosphoketolase | cytoplasm | cytoplasm | inside |
| BAQ33077 | bifunctional GMP synthase and glutamine amidotransferase protein | plasmatic membrane | cytoplasm | both |
| BAQ33083 | phenylalanyl-tRNA ligase alpha subunit | cytoplasm | cytoplasm | inside |
| BAQ33084 | phenylalanyl-tRNA ligase beta subunit | cytoplasm | cytoplasm | inside |
| BAQ33088 | tyrosyl-tRNA ligase | cytoplasm | cytoplasm | inside |
| BAQ33095 | conserved hypothetical protein | cytoplasm | cytoplasm | inside |
| BAQ33096 | preprotein translocase subunit * (*secA*) | cytoplasm | cytoplasm | inside |
| BAQ33102 | RNA polymerase sigma factor | cytoplasm | Cytoplasm/extracellular | both |
| BAQ33117 | conserved hypothetical protein | cytoplasm | Extracellular | both |
| BAQ33121 | peptidyl-prolyl cis-trans isomerase | cytoplasm | cytoplasm | inside |
| BAQ33132 | trigger factor | cytoplasm | cytoplasm | inside |
| BAQ33134 | protease Clp proteolytic subunit | plasmatic membrane | cytoplasm | both |
| BAQ33136 | DNA-binding protein | cytoplasm | cytoplasm | inside |
| BAQ33138 | adenylosuccinate lyase | cytoplasm | cytoplasm | inside |
| BAQ33149 | chaperonin GroEL * | cytoplasm | cytoplasm | inside |
| BAQ33152 | two-component response regulator | cytoplasm | cytoplasm | inside |
| BAQ33154 | cold shock protein | cytoplasm | cytoplasm | inside |
| BAQ33155 | conserved hypothetical protein | cytoplasm | cell membrane | both |
| BAQ33156 | ATP-dependent Clp protease ATP-binding subunit | cytoplasm | Cell membrane/cytoplasm | both |
| BAQ33195 | Ribosome-binding ATPase | cytoplasm | cytoplasm | inside |
| BAQ33200 | conserved hypothetical protein | plasmatic membrane | cell membrane | surface associated |
| BAQ33208 | Enolase * (*eno*) | plasmatic membrane | cytoplasm | both |
| BAQ33209 | L-lactate dehydrogenase | unknown | cytoplasm | both |
| BAQ33210 | conserved hypothetical protein | plasmatic membrane | Extracellular | surface associated |
| BAQ33222 | leucyl-tRNA ligase | cytoplasm  (ambiguous) | cytoplasm | both |
| BAQ33226 | conserved hypothetical protein | cytoplasm | cytoplasm | inside |
| BAQ33240 | orotate phosphoribosyltransferase | cytoplasm | cytoplasm | inside |
| BAQ33242 | elongation factor P | cytoplasm | cytoplasm | inside |
| BAQ33244 | carbamoyl phosphate synthase small subunit | cytoplasm | cytoplasm | inside |
| BAQ33245 | carbamoyl phosphate synthase large subunit | cytoplasm | Cell membrane/cytoplasm | both |
| BAQ33247 | guanylate kinase | cytoplasm | cytoplasm | inside |
| BAQ33248 | DNA-directed RNA polymerase omega subunit | cytoplasm | Cell membrane/cytoplasm | both |
| BAQ33257 | conserved hypothetical protein | cytoplasm | Cell membrane/cytoplasm | both |
| BAQ33273 | transaldolase | cytoplasm | cytoplasm | inside |
| BAQ33274 | transketolase | cytoplasm | cytoplasm | inside |
| BAQ33277 | methionyl-tRNA formyltransferase | unknown | cytoplasm | inside |
| BAQ33280 | conserved hypothetical protein | plasmatic membrane | cytoplasm | both |
| BAQ33288 | long-chain-fatty acid CoA ligase | plasmatic membrane | cytoplasm | both |
| BAQ33290 | 30S ribosomal protein S2 | cytoplasm | Cell membrane/cytoplasm | both |
| BAQ33291 | elongation factor Ts * (*tsf*) | cytoplasm | cytoplasm | inside |
| BAQ33292 | uridylate kinase | cytoplasm | cytoplasm | inside |
| BAQ33293 | ribosome recycling factor | cytoplasm | cytoplasm | inside |
| BAQ33303 | truncated hydrolase | cytoplasm  (ambiguous) | Cell membrane/cytoplasm | both |
| BAQ33304 | RNA polymerase-binding protein | cytoplasm | cytoplasm | inside |
| BAQ33307 | conserved hypothetical protein | cytoplasm | Cell membrane/cytoplasm | both |
| BAQ33313 | aspartyl-tRNA sinthetase | cytoplasm | cytoplasm | inside |
| BAQ33314 | histidyl-tRNA ligase | cytoplasm | cytoplasm | inside |
| BAQ33315 | conserved hypothetical protein | cytoplasm | cytoplasm | inside |
| BAQ33322 | dehydrogenase | cytoplasm | cytoplasm | inside |
| BAQ33340 | conserved hypothetical protein | cytoplasm | cytoplasm | inside |
| BAQ33345 | gamma-glutamyl phosphate reductase | cytoplasm | cytoplasm | inside |
| BAQ33357 | phosphoribosylformylglycinamidine cyclo-ligase | cytoplasm | cytoplasm | inside |
| BAQ33363 | phosphoribosylaminoimidazole carboxylase ATPase subunit | cytoplasm (ambiguous) | cell membrane | surface associated |
| BAQ33365 | ABC transporter ATP-binding component | plasmatic membrane | cell membrane | surface associated |
| BAQ33368 | conserved hypothetical protein | plasmatic membrane | Extracellular | surface associated |
| BAQ33371 | 30S ribosomal protein S20 | cytoplasm | Cytoplasm/extracellular | both |
| BAQ33399 | pyridoxal 5´-phosphate synthase subunit | plasmatic membrane | cytoplasm | both |
| BAQ33408 | conserved hypothetical protein | plasmatic membrane | cell membrane/extracellular | surface associated |
| BAQ33410 | cell division ATP-binding protein FtsE | cytoplasm | cell membrane | both |
| BAQ33411 | peptide chain release factor 2 | cytoplasm | cytoplasm | inside |
| BAQ33412 | methionine aminopeptidase | cytoplasm | cytoplasm | inside |
| BAQ33413 | metalloendopeptidase | plasmatic membrane | cytoplasm | both |
| BAQ33416 | prolyl-tRNA ligase | cytoplasm | cytoplasm | inside |
| BAQ33418 | oligoribonuclease | cytoplasm | cytoplasm | inside |
| BAQ33421 | inosine-5'-monophosphate dehydrogenase (GuaB) * (*impd*) | cytoplasm  (ambiguous) | cytoplasm | both |
| BAQ33425 | peptide chain release factor 1 | cytoplasm | cytoplasm | inside |
| BAQ33427 | putative cell surface protein | plasmatic membrane | extracellular | surface associated |
| BAQ33428 | 50S ribosomal protein L25 | cytoplasm | cytoplasm | inside |
| BAQ33431 | hypothetical protein | plasmatic membrane | cell membrane | surface associated |
| BAQ33436 | conserved hypothetical protein | cytoplasm | Cell membrane/cytoplasm | both |
| BAQ33444 | conserved hypothetical protein | cytoplasm | Cytoplasm/extracellular | both |
| BAQ33450 | pyruvate kinase * (*pyK*) | cytoplasm | cytoplasm | inside |
| BAQ33456 | 30S ribosomal protein S1 | plasmatic membrane | cytoplasm | both |
| BAQ33457 | bifunctional methylenetetrahydrofolate dehydrogenase and methenyltetrahydrofolate cyclohydrolase | cytoplasm | cytoplasm | inside |
| BAQ33477 | NADH oxidase | cytoplasm  (ambiguous) | cytoplasm | both |
| BAQ33480 | YhgE/Pip N-terminal domain-containing protein | plasmatic membrane | cell membrane | surface associated |
| BAQ33494 | cysteine synthase | cytoplasm | cytoplasm | inside |
| BAQ33535 | conserved hypothetical protein | cytoplasm | Extracellular | both |
| BAQ33541 | CTP synthase | cytoplasm | cytoplasm | inside |
| BAQ33544 | chorismate synthase | cytoplasm | cytoplasm | inside |
| BAQ33548 | alanyl-tRNA ligase | plasmatic membrane | cytoplasm | both |
| BAQ33552 | galactose-1-phosphate uridylyltransferase | cytoplasm | Cytoplasm/extracellular | both |
| BAQ33553 | 30S ribosomal protein S4 | cytoplasm | cytoplasm | inside |
| BAQ33576 | conserved hypothetical protein | plasmatic membrane | cytoplasm | both |
| BAQ33577 | ABC transporter ATP-binding component | cytoplasm | cell membrane | both |
| BAQ33579 | ABC transporter permease component | cytoplasm | cytoplasm | inside |
| BAQ33586 | conserved hypothetical protein | cytoplasm | cytoplasm | inside |
| BAQ33590 | cysteinyl-tRNA ligase | cytoplasm | cytoplasm | inside |
| BAQ33593 | 30S ribosomal protein S16 | cytoplasm | cytoplasm | inside |
| BAQ33594 | conserved hypothetical protein | cytoplasm | cytoplasm | inside |
| BAQ33600 | conserved hypothetical protein | plasmatic membrane | cytoplasm | both |
| BAQ33602 | 50S ribosomal protein L28 | cytoplasm | cell membrane/extracellular | both |
| BAQ33606 | conserved hypothetical protein | plasmatic membrane | cell membrane/extracellular | surface associated |
| BAQ33607 | 50S ribosomal protein L9 | cytoplasm | cytoplasm | inside |
| BAQ33608 | 30S ribosomal protein S18 | cytoplasm | Cell membrane/cytoplasm | both |
| BAQ33610 | 30S ribosomal protein S6 | cytoplasm | cytoplasm | inside |
| BAQ33615 | 50S ribosomal protein L20 | cytoplasm | cell membrane | both |
| BAQ33616 | 50S ribosomal protein L35 | cytoplasm | Cytoplasm/extracellular | both |
| BAQ33619 | glyceraldehyde-3-phosphate dehydrogenase * (*gap*) | cytoplasm | cytoplasm | inside |
| BAQ33620 | conserved hypothetical protein | cytoplasm | cytoplasm | inside |
| BAQ33625 | UDP-glucose 4-epimerase | cytoplasm | cytoplasm | inside |
| BAQ33632 | galactokinase | cytoplasm | cytoplasm | inside |
| BAQ33633 | galactose-1-phosphate uridylyltransferase | cytoplasm | cytoplasm | inside |
| BAQ33639 | Xaa-Pro aminopeptidase | cytoplasm | cytoplasm | inside |
| BAQ33644 | hypothetical protein | plasmatic membrane | cell membrane | surface associated |
| BAQ33652 | cell wall associated fibronectin-binding protein | plasmatic membrane | Extracellular | surface associated |
| BAQ33657 | DNA-directed RNA polymerase beta' subunit | cytoplasm | cytoplasm | inside |
| BAQ33658 | DNA-directed RNA polymerase beta subunit | cytoplasm | cytoplasm | inside |
| BAQ33666 | 50S ribosomal protein L19 * (*rplS*) | cytoplasm | cytoplasm | inside |
| BAQ33667 | nitrate ABC transporter ATP-binding component | plasmatic membrane | cell membrane | surface associated |
| BAQ33672 | putative cell surface protein | plasmatic membrane | cell wall/extracellular | surface associated |
| BAQ33673 | amylopullulanase | plasmatic membrane | Extracellular | surface associated |
| BAQ33676 | conserved hypothetical protein | cytoplasm | cell membrane | both |
| BAQ33706 | 30S ribosomal protein S11 | cytoplasm | Cell membrane/cytoplasm | both |
| BAQ33707 | 30S ribosomal protein S13 | cytoplasm | cytoplasm | inside |
| BAQ33709 | translation initiation factor IF-1 | cytoplasm | cytoplasm | inside |
| BAQ33710 | adenylate kinase | cytoplasm | cytoplasm | inside |
| BAQ33712 | 50S ribosomal protein L15 | cytoplasm | cytoplasm | inside |
| BAQ33713 | 50S ribosomal protein L30 | cytoplasm | cytoplasm | inside |
| BAQ33714 | 30S ribosomal protein S5 | cytoplasm | cytoplasm | inside |
| BAQ33715 | 50S ribosomal protein L18 | cytoplasm | cytoplasm | inside |
| BAQ33716 | 50S ribosomal protein L6 | cytoplasm | cytoplasm | inside |
| BAQ33717 | 30S ribosomal protein S8 * (*rpsH*) | cytoplasm | cytoplasm | inside |
| BAQ33719 | 50S ribosomal protein L5 * (*rplE*) | cytoplasm | cytoplasm | inside |
| BAQ33720 | 50S ribosomal protein L24 | cytoplasm | cytoplasm | inside |
| BAQ33721 | 50S ribosomal protein L14 | cytoplasm | cytoplasm | inside |
| BAQ33722 | 30S ribosomal protein S17 | cytoplasm  (ambiguous) | cytoplasm | both |
| BAQ33723 | 50S ribosomal protein L29 | cytoplasm | cytoplasm | inside |
| BAQ33724 | 50S ribosomal protein L16 | cytoplasm  (ambiguous) | cytoplasm | both |
| BAQ33725 | 30S ribosomal protein S3 | cytoplasm | cytoplasm | inside |
| BAQ33726 | 50S ribosomal protein L22 | cytoplasm | cell membrane | both |
| BAQ33727 | 30S ribosomal protein S19 | cytoplasm | cytoplasm | inside |
| BAQ33728 | 50S ribosomal protein | cytoplasm | Cytoplasm/extracellular | both |
| BAQ33729 | 50S ribosomal protein L23 | cytoplasm | Cell membrane/cytoplasm | both |
| BAQ33730 | 50S ribosomal protein L4 | cytoplasm | cell membrane | both |
| BAQ33732 | 30S ribosomal protein S10 | cytoplasm | cytoplasm | inside |
| BAQ33734 | 30S ribosomal protein S9 * (*rpsI*) | cytoplasm | cell membrane/extracellular | both |
| BAQ33735 | 50S ribosomal protein L13 | cytoplasm | cytoplasm | inside |
| BAQ33737 | conserved hypothetical protein | cytoplasm | Extracellular | both |
| BAQ33746 | 50S ribosomal protein L33 | cytoplasm | cell membrane | both |
| BAQ33748 | chaperone GroES | cytoplasm | cytoplasm | inside |
| BAQ33754 | conserved hypothetical protein | cytoplasm | Extracellular | both |
| BAQ33755 | glutamyl-tRNA ligase | cytoplasm | cytoplasm | inside |
| BAQ33756 | conserved hypothetical protein | cytoplasm | Extracellular | both |
| BAQ33766 | arginyl-tRNA ligase | cytoplasm | cytoplasm | inside |
| BAQ33774 | glucose-6-phosphate 1-dehydrogenase | cytoplasm | cytoplasm | inside |
| BAQ33778 | 6-phosphogluconate dehydrogenase | plasmatic membrane | cytoplasm | both |
| BAQ33781 | ribonucleotide-diphosphate reductase alpha subunit | cytoplasm | cytoplasm | inside |
| BAQ33782 | ribonucleoside-diphosphate reductase beta subunit | plasmatic membrane | cytoplasm | both |
| BAQ33805 | high-affinity Fe2+/Pb2+ permease | plasmatic membrane | Extracellular | surface associated |
| BAQ33816 | hypothetical protein | plasmatic membrane | cell membrane/extracellular | surface associated |
| BAQ33818 | ATP synthase beta subunit (AtpD) | cytoplasm | Cell membrane/cytoplasm | both |
| BAQ33820 | ATP synthase alpha subunit (AtpA) | cytoplasm  (ambiguous) | Cell membrane | surface associated |
| BAQ33822 | ATP synthase subunit B | plasmatic membrane | cell membrane/extracellular | surface associated |
| BAQ33864 | transcriptional regulator | cytoplasm | cell membrane/cytoplasm | both |
| BAQ33865 | transcription termination factor | plasmatic membrane | cytoplasm | both |
| BAQ33868 | putative acetyltransferase | cytoplasm | cytoplasm | inside |
| BAQ33869 | aspartyl/glutamyl-tRNA amidotransferase subunit B | cytoplasm | cytoplasm | inside |
| BAQ33870 | aspartyl/glutamyl-tRNA amidotransferase subunit A | cytoplasm | cytoplasm | inside |
| BAQ33882 | chaperone ClpB | plasmatic membrane | cytoplasm | both |
| BAQ33883 | 2-hydroxyhepta-2,4-diene-1,7-dioate isomerase | plasmatic membrane | cytoplasm | both |
| BAQ33887 | polyphosphate kinase | cytoplasm | Cytoplasm/extracellular | both |
| BAQ33893 | uracil phosphoribosyltransferase | cytoplasm | cytoplasm | inside |
| BAQ33899 | putative signal recognition particle-docking protein FtsY | outside | Cell membrane | surface associated |
| BAQ33910 | ABC transporter ATP-binding component | cytoplasm | cell membrane | both |
| BAQ33912 | conserved hypothetical protein | plasmatic membrane | Extracellular | surface associated |
| BAQ33922 | chromosome partitioning protein ParB | outside | cytoplasm | both |
| BAQ33925 | conserved hypothetical protein | cytoplasm | cytoplasm | inside |

a) Protein ID and description from (<http://www.ncbi.nlm.nih.gov/Taxonomy/Browser/wwwtax.cgi?id=585528>), listed in numerical order of protein ID.

b) Subcellular localization analysis using PSORT server (<http://psort.hgc.jp/form.html>).

c) Subcellular localization analysis using Gpos-mPLoc server (<http://www.csbio.sjtu.edu.cn/bioinf/Gpos-multi/>).

d) Subcellular classification according to results with the two bioinformatics tools used: i) **Inside**: if a protein was predicted to have exclusively cytoplasmic location with the two algorithms was considered it to be inside of the cell, ii) **Both**: if one of the algorithms predicted that the subcellular localization is intracellular (cytoplasmic) and the other predicted that is outside of the cytoplasm (i.e. membrane, cell wall associated and/or extracellular); the protein was considered to be both inside and outside of the cytoplasm, iii) **Surface associated**: if a protein was predicted to have an exclusively extra-cytoplasmic location (i.e. membrane, cell wall associated and/or extracellular) with the two algorithms was considered as surface-associated.

Proteins indicated with * are considered as moonlighting or at least with dual localization in other Gram-positive microorganisms.
