# Supplementary material for: Unraveling Gardnerella vaginalis Surface Proteins Using Cell Shaving Proteomics
Source: Front Microbiol. 2018 May 15;9:975. doi: 10.3389/fmicb.2018.00975 (PMC5962675; doi:10.3389/fmicb.2018.00975)
Supplement: Supplementary file 3 [file Table_3.DOCX]

**Table S3.** Analysis of signal peptide and transmembrane alpha-helix domains of identified proteins in *G. vaginalis* using bioinformatic tools.

| Protein_ID^a)^ | SignalP^b)^ | tatP^c)^ | PRED-LIPO^d)^ | PSORT^e)^ | PROTTER^f)^ | TMHMM^g)^ |
| --- | --- | --- | --- | --- | --- | --- |
| BAQ32694 | Yes | - | Yes | Yes / 1TMD | Yes / 1TMD | 2TMD |
| BAQ32696 | Yes | - | Yes | Yes / - | Yes / - | 1TMD |
| BAQ32758 | Yes | - | Yes | Yes / 1TMD | Yes / 1TMD | 1TMD |
| BAQ32762 | Yes | Yes | Yes* | Yes / - | Yes / - | 1TMD |
| BAQ32771 | Yes | Yes | Yes | - / 2TMD | Yes / 1TMD | 2TMD |
| BAQ32781 | - | Yes | - | - / 2TMD | - / 1 TMD | 1TMD |
| BAQ32792 | Yes | - | Yes | Yes / 1TMD | Yes / 1TMD | 1TMD |
| BAQ32803 | - | Yes | Yes* | Yes / - | Yes / - | 1TMD |
| BAQ32810 | - | - | - | Yes / - | - | - |
| BAQ32815 | Yes | - | Yes | Yes / 1TMD | Yes / 1TMD | 2TMD |
| BAQ32817 | Yes | - | Yes | Yes / 1TMD | Yes / 1TMD | 2TMD |
| BAQ32818 | Yes | - | Yes* | Yes | Yes / - | - |
| BAQ32829 | Yes | Yes | Yes | Yes / 1TMD | Yes / 1TMD | 2TMD |
| BAQ32876 | Yes | - | Yes* | Yes / - | Yes / - | 1TMD |
| BAQ32957 | Yes | - | Yes* | Yes | Yes / - | - |
| BAQ32990 | - | - | - | - / 1TMD | - / 1TMD | 1TMD |
| BAQ33200 | - | - | - | Yes / 7TMD | - / 7TMD | 7TMD |
| BAQ33209 | - | - | Yes | - / - | - / - | - |
| BAQ33363 | - | Yes | - | - / - | - / - | - |
| BAQ33368 | - | Yes | - | - / 2TMD | - / 2TMD | 2TMD |
| BAQ33408 | Yes | - | - | - / 1TMD | Yes / - | 1TMD |
| BAQ33427 | - | - | Yes | - / 2TMD | - / 1TMD | 1TMD |
| BAQ33431 | Yes | - | Yes | Yes / 1TMD | Yes / 1TMD | 1TMD |
| BAQ33606 | Yes | - | Yes | - / 2TMD | Yes / 1TMD | 2TMD |
| BAQ33644 | Yes | Yes | Yes | Yes / 1TMD | Yes / 1TMD | 2TMD |
| BAQ33652 | Yes | - | Yes | Yes / 1TMD | Yes / 1TMD | 2TMD |
| BAQ33672 | - | - | Yes | - / 1TMD | Yes / 1TMD | 2TMD |
| BAQ33673 | Yes | - | Yes | Yes / 1TMD | Yes / 1TMD | 2TMD |
| BAQ33722 | - | Yes | - | - / - | - / - | - |
| BAQ33724 | - | Yes | - | - / - | - / - | - |
| BAQ33805 | Yes | - | - | Yes / 7TMD | - / 8TMD | 8TMD |
| BAQ33816 | Yes | - | Yes | Yes / 1TMD | Yes / 1TMD | 2TMD |
| BAQ33922 | Yes | - | Yes | Yes / - | Yes / - | - |
| BAQ32664 | - | - | - | - / 1TMD | - / 1TMD | 1TMD |
| BAQ32680 | - | - | - | - / 1TMD | - / - | - |
| BAQ32710 | - | - | - | - / 1TMD | - / 1TMD | 1TMD |
| BAQ32759 | - | - | - | - / 1TMD | - / 1TMD | - |
| BAQ32791 | - | - | - | - / 1TMD | - / - | - |
| BAQ32802 | - | - | - | - / 1TMD | - / - | - |
| BAQ32822 | - | - | - | Yes / 1TMD | Yes / - | - |
| BAQ32840 | - | - | - | - / 1TMD | - / - | - |
| BAQ32845 | - | - | - | - / 1TMD | - / - | - |
| BAQ32898 | - | - | - | - / 1TMD | - / 1TMD | 1TMD |
| BAQ32907 | - | - | - | - / 1TMD | - / - | - |
| BAQ32935 | - | - | - | - / 1TMD | - / - | - |
| BAQ32936 | - | - | - | - / 1TMD | - / - | - |
| BAQ32945 | - | - | - | - / 1TMD | - / - | - |
| BAQ32970 | - | - | - | - / - | - / 1TMD | 1TMD |
| BAQ32974 | - | - | - | - / 1TMD | - / - | - |
| BAQ33051 | - | - | - | - / 2TMD | - / 2TMD | 2TMD |
| BAQ33075 | - | - | - | - / 2TMD | - / - | - |
| BAQ33077 | - | - | - | - / 1TMD | - / - | - |
| BAQ33134 | - | - | - | - / 1TMD | - / - | - |
| BAQ33208 | - | - | - | - / 1TMD | - / - | - |
| BAQ33210 | - | - | - | - / 1TMD | - / 1TMD | 1TMD |
| BAQ33280 | - | - | - | - / 1TMD | - / - | - |
| BAQ33288 | - | - | - | - / 1TMD | - / - | - |
| BAQ33365 | - | - | - | - / 1TMD | - / - | - |
| BAQ33399 | - | - | - | - / 1TMD | - / - | - |
| BAQ33413 | - | - | - | - / 1TMD | - / - | - |
| BAQ33456 | - | - | - | - / 1TMD | - / - | - |
| BAQ33480 | - | - | - | - / 6TMD | - / 7TMD | 6TMD |
| BAQ33548 | - | - | - | - / 1TMD | - / - | - |
| BAQ33576 | - | - | - | - / 1TMD | - / - | - |
| BAQ33600 | - | - | - | - / 1TMD | - / - | - |
| BAQ33667 | - | - | - | - / 1TMD | - / - | - |
| BAQ33778 | - | - | - | Yes / 1TMD | - / - | - |
| BAQ33782 | - | - | - | - / 1TMD | - / 1TMD | 1TMD |
| BAQ33822 | - | - | - | Yes / 1TMD | - / 1TMD | 1TMD |
| BAQ33865 | - | - | - | - / 1TMD | - / - | - |
| BAQ33882 | - | - | - | - / 1TMD | - / - | - |
| BAQ33883 | - | - | - | - / 1TMD | - / - | - |
| BAQ33899 | - | - | - | Yes / - | - / 1TMD | 1TMD |
| BAQ33912 | - | - | - | - / 1TMD | - / 1TMD | 1TMD |

*a) G. vaginalis* ATCC14018 Protein_ID from GeneBank

(<http://www.ncbi.nlm.nih.gov/genome/1967?genome_assembly_id=233483>).

b) SignalP server v.4.1 (<http://www.cbs.dtu.dk/services/SignalP>) predicts sec signal peptide of Sec secretion pathway.

c) TatP server v.1.0 (<http://www.cbs.dtu.dk/services/TatP>) predicts tat signal peptide of Tat secretion pathway.

d) PRED-LIPO server (<http://www.compgen.org/tools/PRED-LIPO>) predicts sec signal peptide and lipo signal peptide (indicated with *).

e) PSORT server (<http://psort.hgc.jp/form.html>) predicts sec signal peptide and alpha-helix transmembrane domain (TMD).

f) PROTTER server ([http://wlab.ethz.ch/protter/#](http://wlab.ethz.ch/protter/)) predicts sec signal peptide and TMDs.

g) TMHMM server v.2.0 (<http://www.cbs.dtu.dk/services/TMHMM>) predicts TMDs.

Positive prediction is indicated with Yes for signal peptide and number of transmembrane domains (TMD), no prediction is indicated with -.
