# Supplementary material for: Unraveling Gardnerella vaginalis Surface Proteins Using Cell Shaving Proteomics
Source: Front Microbiol. 2018 May 15;9:975. doi: 10.3389/fmicb.2018.00975 (PMC5962675; doi:10.3389/fmicb.2018.00975)
Supplement: Supplementary file 4 [file Table_4.DOCX]

**Table S4.** Analysis of *G. vaginalis* identified proteins belonging to the ABC superfamily by Pfam sequence search and blastp.

| Pfam ID  (accession number)^a^ | Pfam family description^a^ | Protein_ID^b^ | Description^b^ | Blastp against ATCC14019^c^ (%identity) |
| --- | --- | --- | --- | --- |
| ABC_tran (PF00005) | ATP-binding domain of ABC transporters | BAQ32802 | Dipeptide/oligopeptide ABC transporter ATP-binding component | Peptide ABC transporter ATP-binding protein (100%) |
|  |  | BAQ32877 | Putative sugar ABC transporter ATP-binding component | L-arabinose ABC transporter ATP-binding domain (100%) |
|  |  | BAQ32949 | ABC transporter ATP-binding component | ABC-transporter ATP-binding protein (100%) |
|  |  | BAQ33365 | ABC transporter ATP-binding component | Multidrug ABC transporter ATP-binding protein (100%) |
|  |  | BAQ33577 | ABC transporter ATP-binding component | ABC-transporter ATP-binding protein (100%) |
|  |  | BAQ33667 | Nitrate ABC transporter ATP-binding component | ABC-transporter ATP-binding protein (100%) |
|  |  | BAQ33910 | ABC transporter ATP-binding component | ABC-transporter ATP-binding protein (100%) |
| SBP_bac_5 (PF00496) | Bacterial extracellular solute-binding proteins, family 5 Middle | BAQ32803 | Dipeptide/oligopeptide ABC transporter ATP-binding component | ABC transporter substrate-binding protein (100%) |
|  |  | BAQ32990 | Putative ABC transporter substrate binding component | ABC transporter substrate-binding protein (100%) |
| SBP_bac_8 (PF13416) | Bacterial extracellular solute-binding protein | BAQ32762 | Putative ABC transporter substrate binding component | ABC transporter substrate-binding protein (99%) |
| ZnuA (PF01297) | Zinc-uptake complex component A periplasmic | BAQ32818 | Putative ABC transporter substrate binding component | ABC transporter substrate-binding protein (100%) |
| Peripla_BP_4 (PF13407) | Periplasmic binding protein domain | BAQ32876 | Putative sugar ABC transporter substrate binding component | ABC transporter substrate-binding protein (100%) |
| UPF0051 (PF01458) | Uncharacterized protein family | BAQ33579 | ABC transporter permease component | ABC-transporter ATP-binding protein (100%) |

a) Family and description of Pfam 28.0 (May 2015, 16230 families) server sequence search (<http://pfam.xfam.org/search>).

b) Protein ID and description from Genome Project of *G. vaginalis* JCM 11026 (<http://www.ncbi.nlm.nih.gov/Taxonomy/Browser/wwwtax.cgi?id=585528>).

c) Protein blast in order to find homology with *G. vaginalis* ATCC14019 proteome (<http://blast.ncbi.nlm.nih.gov/Blast.cgi?PAGE=Proteins>).
