# Supplementary material for: Unraveling Gardnerella vaginalis Surface Proteins Using Cell Shaving Proteomics
Source: Front Microbiol. 2018 May 15;9:975. doi: 10.3389/fmicb.2018.00975 (PMC5962675; doi:10.3389/fmicb.2018.00975)
Supplement: Supplementary file 5 [file Table_5.DOCX]

**Table S5.** Sequence analysis of proteins with unknown function by Pfam and blastp against the *G. vaginalis* ATCC14019 strain.

| protein_ID^a^ | Description^a^ | Pfam ID  (accession number)^b^ | Pfam description^b^ | Blastp against ATCC14019^c^  (%identity) |
| --- | --- | --- | --- | --- |
| BAQ32662 | conserved hypothetical protein | DUF3662 (PF12401) | Protein of unknown function | Hypothetical protein (99%) |
| BAQ32685 | conserved hypothetical protein | Ferritin (PF00210) | Ferritin-like domain | DNA starvation/stationary phase protection protein (100%) |
| BAQ32694 | hypothetical protein | - | - | Peptidase (100%) |
| BAQ32696 | conserved hypothetical protein | DUF2974 (PF11187) | Protein of unknown function | Triacylglycerol lipase (100%) |
| BAQ32771 | conserved hypothetical protein | FIVAR (PF07554) | Uncharacterised sugar-binding domain | Peptidase (99%) |
| BAQ32780 | conserved hypothetical protein | Hydrolase (PF00702) | Haloacid dehalogenase-like hydrolase | Peptidase (99%) |
| BAQ32789 | conserved hypothetical protein | PBP (PF01161) | Phosphatidiylethanolamine-binding protein | Hypothetical protein (100%) |
| BAQ32815 | hypothetical protein | - | - | Hypothetical protein (99%) |
| BAQ32817 | hypothetical protein | - | - | Hypothetical protein(68%) |
| BAQ32829 | hypothetical protein | - | - | - |
| BAQ32849 | conserved hypothetical protein | - | - | Cell division protein (100%) |
| BAQ32898 | conserved hypothetical protein | YkuD (PF03734) | L,D-transpeptidase catalytic domain | Hypothetical protein (99%) |
| BAQ32957 | hypothetical protein | - | - | Hypothetical protein (100%) |
| BAQ33005 | conserved hypothetical protein | - | - | Hypothetical protein (100%) |
| BAQ33018 | conserved hypothetical protein | DivlVA (PF05103) | Involved in septum formation | Dihydrouridine synthase (100%) |
| BAQ33044 | conserved hypothetical protein | Usp (PF00582) | Universal stress protein family | Universal stress protein family (100%) |
| BAQ33049 | conserved hypothetical protein | LMWPc (PF01451) | Protein tyrosine phosphatase | Phosphotyrosine protein phosphatase (100%) |
| BAQ33051 | conserved hypothetical protein | DUF3043 (PF11241) | Protein of unknown function | Membrane protein (100%) |
| BAQ33052 | conserved hypothetical protein | Peptidase_M20 (PF01546) | Peptidase family M20/M25/M40 | Dipeptidase (100%) |
| BAQ33061 | conserved hypothetical protein | Response_reg  (PF00072) | Response regulator receiver domain | Two-component system response regulator (100%) |
| BAQ33095 | conserved hypothetical protein | Ribosom_S30AE_C (PF16321) | Sigma 54 modulation/S30EA ribosomal protein C terminus | RaiA ribosome-associated inhibitor A (100%) |
| BAQ33117 | conserved hypothetical protein | DUF3071 (PF11268) | Protein of unknown function | Hypothetical protein (100%) |
| BAQ33155 | conserved hypothetical protein | DUF3027 (PF11228) | Protein of unknown function | Hypothetical protein (100%) |
| BAQ33200 | conserved hypothetical protein | UPF0182 (PF03699) | Uncharacterised protein family | Hypothetical protein(100%) |
| BAQ33210 | conserved hypothetical protein | DivIC (PF04977) | Septum formation initiator | Septum formation initiator (100%) |
| BAQ33226 | conserved hypothetical protein | TsaE (PF02367) | Threonylcarbamoyl adenosine biosynthesis protein TsaE | tRNAthreonylcarbamoyladenosine biosynthesis protein TsaE (99%) |
| BAQ33257 | conserved hypothetical protein | Hydrolase_3 (PF08282) | Haloacid dehydrogenase superfamily | Haloacid dehalogenase (99%) |
| BAQ33280 | conserved hypothetical protein | ROK (PF00480) | ROK family | Polyphosphate glucokinase(100%) |
| BAQ33307 | conserved hypothetical protein | WYL (PF13280) | C-terminus of a DNA-binding helix-turn-helix domain | WYL domain-containing protein (100%) |
| BAQ33315 | conserved hypothetical protein | - | - | DNA repair ATPase (100%) |
| BAQ33340 | conserved hypothetical protein | Transcrip_reg (PF01709) | Transcriptional regulator | Transcriptional regulator (100%) |
| BAQ33368 | conserved hypothetical protein | - | - | Membrane protein (100%) |
| BAQ33408 | conserved hypothetical protein | CHAP (FP05257) | Cysteine, Histidine-dependent Amidohydrolases/Peptidases domain | Amidase (100%) |
| BAQ33427 | putative cell surface protein | Flg_new (FP09479) | *Listeria*-bacteroides repeat domain | Hypothetical protein (99%) |
| BAQ33431 | hypothetical protein | - | - | Hypothetical protein (100%) |
| BAQ33436 | conserved hypothetical protein | DcpS_C (FP11969) | Scavenger mRNA decapping enzyme C-terminal binding | Hydrolase (100%) |
| BAQ33444 | conserved hypothetical protein | Pyridox_oxidase (FP01243) | Pyridoxamine 5´-phosphate oxidase | Pyridoxamine 5´-phosphate oxidase (100%) |
| BAQ33535 | conserved hypothetical protein | DUF3710 (FP12502) | Protein of unknown function | Hypothetical protein (100%) |
| BAQ33576 | conserved hypothetical protein | GTP_EFTU (FP00009) | GTP-binding elongation factor family, EF-Tu/EF-1A subfamily | GTP binding protein (100%) |
| BAQ33586 | conserved hypothetical protein | - | - | Hypothetical protein (100%)  SPFH_like superfamily |
| BAQ33594 | conserved hypothetical protein | KH_4 (FP13083) | K homology domain present in nucleic acid-binding proteins | RNA-binding protein (100%) |
| BAQ33600 | conserved hypothetical protein | PfkB (FP00294) | PfkB family carbohydrate kinase | Kinase (99%) |
| BAQ33606 | conserved hypothetical protein | FIVAR (PF07554) | Uncharacterised sugar-binding domain | Peptidase (99%) |
| BAQ33620 | conserved hypothetical protein | tRNA_edit (PF04173) | YbaK protein domain involved in oligonucleotide binding | Aminoacyl-tRNAdeacylase(100%) |
| BAQ33644 | hypothetical protein | - | - | Hypothetical protein (100%) |
| BAQ33672 | putative cell surface protein | Rib (PF08428) | Rib/alpha-like repeat present in bacterial surface proteins | Peptidase (99%) |
| BAQ33676 | conserved hypothetical protein | CPSase_L_D2 (FP02786) | Carbamoyl-phosphate synthase L chain, ATP binding domain | Carboxylate-amine ligase (100%) |
| BAQ33754 | conserved hypothetical protein | FHA (FP00498) | Forkhead-associated domain | Hypothetical protein (100%) |
| BAQ33756 | conserved hypothetical protein | - | - | Hypothetical protein (100%) |
| BAQ33816 | hypothetical protein | - | - | Hypothetical protein (100%) |
| BAQ33912 | conserved hypothetical protein | Thioredoxin_4 (FP13462) | Thioredoxin | DSBA oxidoreductase (100%) |
| BAQ33925 | conserved hypothetical protein | R3H (FP01424) | R3H domain with highly conserved arginine and histidine residues, binding to ssDNA | Hypothetical protein (100%) |

a) Protein ID and description from Genome Project of *G. vaginalis* JCM 11026 (<http://www.ncbi.nlm.nih.gov/Taxonomy/Browser/wwwtax.cgi?id=585528>).

b) Family and description of Pfam 28.0 (May 2015, 16230 families) server sequence search (<http://pfam.xfam.org/search>).

c) Protein blast in order to find homology with *G. vaginalis* ATCC14019 proteome (http://blast.ncbi.nlm.nih.gov/Blast.cgi?PAGE=Proteins).
