# Supplementary material for: Unraveling Gardnerella vaginalis Surface Proteins Using Cell Shaving Proteomics
Source: Front Microbiol. 2018 May 15;9:975. doi: 10.3389/fmicb.2018.00975 (PMC5962675; doi:10.3389/fmicb.2018.00975)
Supplement: Supplementary file 6 [file Data_Sheet_1.DOCX]

**Supplemental data**

**Mapping of identified peptides and bioinformatic analysis of the primary sequence of proteins identified in *G. vaginalis* surfome.**

These proteins included those with the one of the following characteristics predicted: of two or more TMDs, an unknown subcellular localization, LPXTG motif, lipobox domain or tat-SP. Furthermore, this analysis was also performed with the proteins that were identified with greater than 10 peptides on average and with predicted extra-cytoplasmic subcellular location listed in Table 1. The protein_ID is shown in yellow. Transmembrane domain (TMD) and signal peptides (SP) determined by the bioinformatics tools described in Material and Methods are shown in red and in green, respectively. Peptides identified by MS are marked in blue. Underlined aminoacids correspond to the lipobox domain and the LPXTG motif is indicated in pink. For the 17 proteins with a predicted lipobox motif, the conserved **c**ysteine is indicated in bold in the primary sequence. A schematic representation of secondary structure of the proteins is shown below following the model obtained with the PROTTER server. The **SP** is showed, “**in**” to indicate the fragment of the protein with cytoplasmic localization, **arrows** represent TMDs (**↑** when the orientation is inside to outside and **↓** when the orientation is outside to inside) and “**out**” to indicate the fragment of the protein with extracellular localization.

>lcl|AP012332.1_prot_BAQ33200.1_548 [gene=GAVG_0548] [protein=conserved hypothetical protein]

**3 unique peptides were identified:**

ELSDAINDAMSEEAVK

SGNWQAYGK

VAPYLTLDGR

MLKVIIEIIIGVARAAFGAYMAKKPENTKVYKRSDYSSDGKKKSNQNNDFADTSADDSIDQDVVVESIDEDNSSVNVVDKDASKDESNSENKDKTSSKFAKKIKDLADYYSDDDDLGDLFSDILEAKKKLENEGDSPKNSDTSNASEDSKDSKSNKSDNKSSNSKENNRYMRIDFDVNKFVGKSRKWRWIIVAIVVAIAFICSIIFGFSTFITDCMWYAQLGFESVIWIQLAAKIGVWALYALLMAAFGYLSAYIAIKKRPGSEDGVYVKEKGNILDTKKGISSKLAMHVAGVVSLIVGAVFGMQFYNHWVQILLMFNYQSFGIKDPQFGFDNGFYVFVLPGLRLFTRAFIVLLAVSLLFSVITNALMGAVRITLPVGGKGIFNITKSARRQISAWFMLVIIFWSVLQILDVFAIVNLDGSKITGGSYTDMNAGVPSSIAMAVITLIVGIVITTWLLKSHALNGNVKIGTRFAVAVKAWRTPAIAVASLVVCAMVLSFAWPALLQRFKVAPNAQELEATYIQRNIDATKFAYGLNNVKKESYNATSEGKSGALAKEAESTAQIRLLDPQVTSPTFRQLQQSKQYYTFADTLSVDKYDIDGVSQDTVIAARELDLAGNDNRNWVNDHTVYTHGYGVVAAYGNKVTADGQPQFMEYGIPTQGKLTKLKKYEPRIYFSPNAPKYSIVGSPKGTAPWEFDYPTGSNGALTTFKGNGGPRVGNFFSRLLHAIRFESDQILFSDRVTSDSQILYDRDPKTRVSKVAPYLTLDGRVYPAVVDGRVKWIVDGYTTSDSYPYSQMTDFGQVTQDSTTTTSRSIKGLTNQRANYIRNSVKATVDAYDGSVDLYVWDKKDPVIKAWRSIFPGHYHDISKISGDLMSHIRYPESLFKVQRHLLAKYHVDSASQFFSGEDFWQTPVDPTESQSLQREDILQPPYYLTLQTRGANKPVFSLVSTYIPAGKITREILTGFLSVDSDAGNVAGKVSENYGKLRLQELPKVSNVPGPGQAQNNFNANANVSKELNLLESGSTKVKRGNLLTLPLGGGLVYVEPVYVQSSGSTSYPLLKKVLVAFGDQVGFADTLDEALNQVFGGNSGANAGDASNNSGSNENVSNKNNANNSNKSDAKSSANAPAKSNSISEKARIALKRAAQALKDSDSAMRSGNWQAYGKAQKELSDAINDAMSEEAVK

**Schematic representation: in-↑-↓-↑-↓-↑-↓-↑-out**

>lcl|AP012332.1_prot_BAQ33805.1_1153 [gene=GAVG_1153][protein=conserved hypothetical protein]

**2 unique peptides were identified:**

AVDLVNEAYYQYYEK

NVMNAISGSR

MHACAKARQKVASASAFLLSICILISCAMGVVGLVGLVGEPTIAYATDADKTYNSWSEVSSAVTSQIEQGKNEYNSANMAGAATRFQAAYNSVYVASNFITVVRDTIGQDKVQSQSDQFQQLQTLVYQQNQSAQISEVASTLERDIAQTASQLDANTQLDKPNVYAAKLRTQIAKERKKLDAAKKKNLGRNGRTWSQVAREMNVILDKSASTYKAAKGNKQEVAKAVDLVNEAYYQYYEKLGFEKNVMNAISGSRVSTVEYQFKECRQAMNNGKTFEQTKKFVTDLKSMLIEDAAKLDGGAASNANPFMQFITSSFGQAFIILLREGLEALLVVAAIIAYLIKSGHKSMVKYIYMGLVAGIIASLIVAALFGLLFNGSGPQQEITEGVVALFAMLMLLYTSNWMISRSSVQAWNEYIRNQTTAAVSKGSLISLALLSFLAVFREGAETVIFYQAIFAVSSGADSMIWGGFVAAAAVLVVIFLLIRFASVRIPIRPFFTGTSVLMCILVVIFAGGGVHALIEGDALAGVYIQGLPTNDWLGFYPYVETISAQVVAAIVVIALLCVSILRTHKSQATVRG

**Schematic representation: in-↑-out-↓-↑-↓-↑-↓-↑-↓-in**

>lcl|AP012332.1_prot_BAQ33480.1_828 [gene=GAVG_0828] [protein=conserved hypothetical protein]

**2 unique peptides were identified:**

IVNGSDQLNSGAK

GGADAISQGFEAVNTGSNK

MRVNTIVLAFRELNNAIKGKARKLVIGTVALIPLLYGSLYLWAFTNPYKTLDTVPVAVVVEDNGAIINGKMRNVGNEIKNRLKNQKDGSEGFQWNFVNSAKANKGLKNGDYFMVATIPASFSKCIASAQYDTPTKAKLHVKYDQASNMLASQIGKTAFRTIRSEISEAIAQEYWEHMFAKVNSASDSLGAAANGAGALHEGSKSLQGGINKLADGSNKLSASLRKLQGGLGELQKGANTLTEANARSKEGAQYLAQKTQDLANGAQQLSEGTQRLSAATDEFYEGAQKLAASSNELEEHVKAGNAETKTKLAGLLMQAQNPAVTKEDVLANLQSLAEGLGENSNFLETNLSALSAGADKLSDGSLQIKGGADAISQGFEAVNTGSNKLAAGANSLAEGLAQVSNGSEKLNNGVNAAAQGSAKIVNGSDQLNSGAKKLSDNTPKLVEGAKKLHDGLKDAQKSGKINNIKQKSKMMSAPVALKEHDYSHVENYGTGFAPYFISIGLWVGALMATFVLRVMDRRAILNGMNPLKSALISFTPFVIMGVVQAVILMGVVHGALKLQIDNVGAFYALGIIASIVFMAIMQMIMVVFKIPGRIVAIVLLMLQITSSAGTFPVQMTPSFFRAVHPYLPMTYSILGLRQAMAGRNSGAIASSIGILVIFGIIAIAITSIVDCVKRTVTMFDLHPVITLEA

**Schematic representation: in-↑-out-↓-↑-↓-↑-↓-↑-in**

>lcl|AP012332.1_prot_BAQ32758.1_106 [gene=GAVG_0106] [protein= M protein repeat protein]

**67 unique peptides were identified:**

KIETETQNIANAEQR

KVSDKESELAELENTIK

AKEEAEKAAQSLLAR

AVEEAKQEAEDTAR

IETETQNIANAEQR

AAEELVENATAK

ESKQDAEKDLNVASR

EAAAEGVDNKIENITK

TNIETAFDLMIK

NNTPANAEILQKVEESK

IDKEISEAKTNVEATK

VTDEAKQNADQVNK

SPATAEIPAR

VSDKESELAELENTIK

NNTPANAEILQK

AAQSLLAR

VTDEAKQNADQVNKIR

TNVEATKQLAETAK

AVEEAKQEAEDTARK

HSAVPANQTESTHTAK

NNAENSIAETNK

LGDPSHIEK

TNIETAFDLMIKEVANR

SKLEAELAEAK

EEAEKAAQSLLAR

LGDPSHIEKLTNAQTK

NLNQLKEDLK

QDAEKDLNVASR

KVQDEKANFENAK

LEAELAEAK

SESTKVEAEKLEK

SKLGDPSHIEK

VQDEKANFENAK

IDKETAEKNLNQLK

ELQKLESDR

KVTDEAKQNADQVNK

SKLGDPSHIEKLTNAQTK

NLNQLKEDLKR

SPATAEIPARTPR

EAQNKHNEANKAVEEAK

KINEAVEK

ILELKASIEETKR

LGDPSHIEKLTNAQTKK

AAASAAPTASSANTTKSEESK

TLDADNDQNKINQLK

AAASAAPTASSANTTK

VEAEKLEKEAQQK

VAEYERELQKLESDR

SKLEAELAEAKQK

ESELAELENTIK

TAEENLKTLDADNDQNK

KAEEELAKADK

KTEAQDIEEQLK

NNAENSIAETNKK

ENPGTVQGSGTSK

IDKEISEAK

LSTEAKKAEEELAK

KAEEELAK

ASIEETKR

NEASTAKENSEKLSTEAK

ESELAELENTIKELK

HNEANKAVEEAK

VEAEKLEK

DAEKAKEEAEK

KSESNSLAK

KQAEEAEKTAAQK

AKILELK

MNAKIAAAISLGASSLALIGSISIANAANAADATTTDTSHNPTAHVKTISDTGFNAVSNVVSYGPEDFKPQTVNSPVTGDADAGKQEKENPGTVQGSGTSKNNTPANAEILQKVEESKNLQKEAQNKHNEANKAVEEAKQEAEDTARKINEAVEKKNNAENSIAETNKKIETETQNIANAEQRIEEKSKAVKEAEKDKKVTDEAKQNADQVNKIRSEEKAAKDELAEDAKDDNEETAEDLANEEEAKSKLEAELAEAKQKSESTKVEAEKLEKEAQQKEAAAEGVDNKIENITKQIKTAEENLKTLDADNDQNKINQLKEKVASTKKQAEEAEKTAAQKQLAYTKQKEKVAEYERELQKLESDRSKLGDPSHIEKLTNAQTKKTEAQDIEEQLKNEASTAKENSEKLSTEAKKAEEELAKADKSSQIRDLLDTISELTSEINSLTRISDNAKDARKAANEKKSESNSLAKKVSDKESELAELENTIKELKESKQDAEKDLNVASRDAEKAKEEAEKAAQSLLARTEEAQKAEKKVQDEKANFENAKIDKETAEKNLNQLKEDLKRIDKEISEAKTNVEATKQLAETAKQKLNDAKTNVEATKAKILELKASIEETKRTLISKYAASKTNIETAFDLMIKEVANRAKELDNSTNTLNAVENSLTAAKQKAEESLNPQVQKPAPSTPAPAPAPSTPQNAPQNNAAPSAPQASAPSVSQSAPQAAPKHSAVPANQTESTHTAKAAEELVENATAKSPATAEIPARTPRAAASAAPTASSANTTKSEESKNTSNDSAKDENQKESEESKNEDSKSEDNSSDKSAQASGANNGEHAADNSTTLIAAIAGSITAGIAAIGAGWHFMRIRRK

**Schematic representation: SP-out-↓-in**

>lcl|AP012332.1_prot_BAQ33431.1_779 [gene=GAVG_0779] [protein=hypothetical protein]

**47 unique peptides were identified:**

QIEDASQQIDSANR

QVQDAEQQVAAATEVK

TNLNEENKNLNAAK

NDNNALQNVNVR

AAGSASEDADEIVK

DTGIFYTFNGDTK

VGSADSLQNMK

AAGSASEDADEIVKLQK

GLKEQELEHVK

FLQYVINTEK

SSYTSLVESAK

ELLDGYENSLKQEK

SDSALSNTDLEKVK

VQYDLNLPDKK

TAENLAWGDYDGHKNGR

VTKQDETVDSVTR

SDSALSNTDLEK

YDNASVMDTTPYK

TLSSSIDAANAK

EAEKLELETK

QAQLEQLK

VKTLSSSIDAANAK

LVESYKNLDNLR

IKETLATSTQKINK

GLKEQELEHVKAEK

AKENNKNEDLIADAER

IREFEESVK

TSEELESKEKELEK

TEVGKADEAVKNANSNLAK

EAAEYYDALNEYRR

YKELLDGYENSLKQEK

EAAEYYDALNEYR

IKENAQLAASNAENAKK

IALDAFTNAAYDFDSAK

ENAQLAASNAENAK

YGDPNADWGIEENAAIAK

IKENAQLAASNAENAK

TAENLAWGDYDGHK

ELLDGYENSLK

DNQYDFGMK

YKELLDGYENSLK

KVQYDLNLPDKK

GKADAAEKVYDAESK

LAQQWQTK

QDETVDSVTR

GESYVIPGQEFQDGSYK

VSEAEKQFNLANK

MNAKIAAAISLGASSLALFAMANVANADVVNQNSDNANVEINANHENPDPQEAKANELAREAEKLELETKTEVGKADEAVKNANSNLAKVNKKVEDASKKASEAIEAKNQAEKDAEDAKKQDAEAKEKVDAAQKIVDEAEKIKENAQLAASNAENAKKQAETQLEQNQNELQNAQKAVDEAKTNLNEENKNLNAAKENLEKEKSSYTSLVESAKSLEEAKKKTDNELKDKETELKEKQAQLEQLKKSDSALSNTDLEKVKQIAKEKESVYKQAQEKVTKIKETLATSTQKINKTSEELESKEKELEKIGDYKKLVESYKNLDNLRGKADAAEKVYDAESKNDSNTENSLEKKSDFYKFLQYVINTEKAKENNKNEDLIADAERAQKILKGESYVIPGQEFQDGSYKEAQTIEAPTWYEDIVKPGLGRVGSADSLQNMKEAAEYYDALNEYRRNDNNALQNVNVRLSLIAESIVHSFYSAAINNHAVNHEKYDNASVMDTTPYKTAENLAWGDYDGHKNGRMKYSNNDCKVLDDGKTSCSKQIKTQSTEPKPTDNKWNALDGWYTIEKNRYEEARDTGIFYTFNGDTKIENKLSEAGKQFLRDNQYDFGMKLSQNPNTRLFEENDENGKLTNLFSLSVGHYTTFARKDISAGGFAEGDLYIQEMTRPSGGKQETVTKNEDVAVWHGSNEYSISVAKYKELLDGYENSLKQEKAAGSASEDADEIVKLQKLNAEANKANANLFEAKYNLRKVQYDLNLPDKKSAKGLKEQELEHVKAEKEKANNEAKKYGDPNADWGIEENAAIAKKLAQQWQTKYTHLNGKVKTLSSSIDAANAKNLPNKIALDAFTNAAYDFDSAKNELKKSQEEFAKLTIEAKKYENEYKVASTNVEEAEKQSQLTGEAKQAKIREFEESVKQLKSTKDDLQIKANDAAKNLAEANEKVTKQDETVDSVTRLLDSARNRVSEAEKQFNLANKTKEKAEQKVAETQSQVNKAQKQIEDASQQIDSANRKVTEAQKQVQDAEQQVAAATEVKNKADATKSNTTKLVSDAAAKAAQAKQKANEAKSAIEQAQRITKEAQSQFESAKKKAENVKMQVLQAKQKVAELHEKVKTLLAGAKNNVIERYNATFAAVNQSFNNFENTLILRFTTLDAVEQSLNESTSTLDKTLKDVNSLVTSDNENNGDGGNNGGNNESPTVPQTNPSHDASTDLTPAPTPSKEVESGNANQNTGSTSATQNNTSNSTSSSESFENYYNQYLAGYANNLFGNEQLGFHAAIPAAQSQSANDANGANGANAQNDNGVAANNVAGAAHTNTLSASSANRIAAALASGSKAAAKDLAKDLAKAQDAAKDSAAKSESNKDNAKNDAKDSAKSKSKSESKNDSKSESSESKDESKNAQASGGAQDGAQSASNNTTLKVVAALAAVVAGIAAIGGGTYFARHRRP

**Schematic representation: SP-out-↓-in**

>lcl|AP012332.1_prot_BAQ33644.1_992 [gene=GAVG_0992] [protein=hypothetical protein]

**36 unique peptides were identified:**

NTAEEAQNGLNAANK

NASTTTANFDGFLDYVIK

ITNITNSISTADSK

AVTNANTAFEQAK

VGGADSLQNMK

NKIDGELTQAVTQAK

EKVAEEAAGDVAAKDK

TAENNVTDKNTALEK

EKVAEEAAGDVAAK

TEADNNLNAANNEVNAK

QLLANYESSLGESAK

GIFLESVGHYTNFASK

SKSEDAQKDVTTADNEVK

VADAQGYLDTATQK

VAEEAAGDVAAKDK

TKVADAQGYLDTATQK

IDALNNPTTTTNEEVER

YKQLLANYESSLGESAK

EAATYYEALNK

QIQNLQGELDQLKEK

ISGFAAAIQK

ALLADAQR

TAADAAKNDAQLQK

KNEAAEQAIKDAK

LFESADDNSKLK

QILNGERVEIMK

QTSAETELKNAK

ISGFAAAIQKHDNEIK

NLNSAEDALEKNPDAK

ITVASAQSALAAAK

ALKDEQAKQTAAEEAK

YDKAVEGTFVTGEK

EKAEAVKTAEQNVEK

AELQENDAINK

IAAALASGAK

NLNSAEDALEKNPDAKK

MNAKLAVAISLGASSLALFGMAGVANAETVTPPNSGTNPQPVVQAPQSGNANPSGNSGNENPQPGSTNQGGNSGQNQPGSTPAGQQVGGNSEGTKPEEKKNEAAEQAIKDAKSKSEDAQKDVTTADNEVKQANEKATEAKNTAEEAQNGLNAANKSKDKADKEKTEADNNLNAANNEVNAKEKVAEEAAGDVAAKDKALKDEQAKQTAAEEAKKKAENEQAAAEEEKKTAENNVTDKNTALEKAKGTLESKQDEKNDYDKKLKEAQEKLENEKKEQARLDDIADRAKTKQEQADLAVENNKKEQQEKQNKIDALNNPTTTTNEEVERLKREASEAEQKAKDAKEDEDKKDKEAKNKQNDLDNAGKNLNSAEDALEKNPDAKKLSDTEKAVKDAKNAADEAAKNASTTTANFDGFLDYVIKTYKNSTSKEDKALLADAQRAKQILNGERVEIMKQVQNGDKTENVLDRVEVPMWYKDLVKLGKVGGADSLQNMKEAATYYEALNKLRKEDSDKNSASTEDKATLGAVNVRLSLIAESIVNSFYSGANHDHAINHPGYKSSEVMKTNPYNATENLAWGNYDGDSKDHKNQEYGSCHITANGNTSCSVDSEKYNALSGWYTVEKERYDKAVEGTFVTGEKGSEVTHKISDAGKKVLKEHRYDLGRYMRQNPDTKLFESADDNSKLKGIFLESVGHYTNFASKDITAGGFAESDLSLQEVTTTTTVNGKSTVSKEDVVGDVAVWHGSNESSITIAKYKQLLANYESSLGESAKAELQENDAINKLNAEANKANYDLFVKKYELRKLHYDLNMSDANNLDAADAAKPEVGLQDQEKNALDVLKQKAQELVAYYSQTDVTWAATQETVNRLLSQAKSEVQKIESKITNITNSISTADSKKSEVEKLKKAVTNANTAFEQAKTDVQEANKALTQAQETANRLNTEATSAQQKYKEAEQKAQTAAQQTSEERNKQIQNLQGELDQLKEKAKTLEQTAKQAKTAADAAKNDAQLQKNKIDGELTQAVTQAKQKVDQAQQAVQDAIKAKDSAVEALAAANKNVTELTEKISGFAAAIQKHDNEIKAAKTKVADAQGYLDTATQKQTSAETELKNAKQTAKEKAEAVKTAEQNVEKAIAAISAAKESVKQAKITVASAQSALAAAKTKVTQAQTKVESYKTKFAAAVAALPEQVRVTYQANFAAVTGNFTTMLATLNSCSTTLSSATSTLQKSAESLSKAVPEPAPEPPDPVPPAPVPPAPVPVPTPPTPPVVPAPPVVPEHHNEGGSNTNTGSGTQEQNTESGNANQNTGSTSATQNNTSSSTSNNTSSSESFENYYNQYLAGYANNLFGNEQLGFHAAIPAAQSQSANDANGANGANAQNANGVAANNVTGAAHANTLSASSANRIAAALASGAKAAAKDLAKAQDAAKDSAAKSESNKDSKSESSESKDESENAQASGGAQDGAQSAYNNTTLKVVAALAAVVAGIAAIGGGTYFARHRRA

**Schematic representation: SP-out-↓-in**

>lcl|AP012332.1_prot_BAQ32771.1_119 [gene=GAVG_0119] [protein=conserved hypothetical protein]

**27 unique peptides were identified:**

YNEELTAQQIQDKLK

YNEELTAQQIQDK

VAIFPSDGNTVDNDKSR

EANPAPAPQVNSEITLSK

TESGSKVESATAVDNK

VVITDNQAYDANQFNR

ATVENKQPEATNYAK

DENQKQSEPVVGQDR

AVAEFTSDVK

VAIFPSDGNTVDNDK

EQPEQTKPTEQTVVK

AQAFTAPEILK

AALDAPTEATGKK

KEQPEQTKPTEQTVVK

SEALALTVAR

TGENGKFVQTPDDPDKK

AALDAPTEATGK

IDKPIVDEVDITK

TAIYEANKDNQELGLSK

FVQTPDDPDKK

TYLVSDLGYK

YEFIVNTDGSAK

NTDKVTTYTTGAQTLK

RATVENKQPEATNYAK

VESATAVDNK

QPEATNYAKDENQK

GVYTNPYQK

MTTKSAKHANKTFGSAKDESAKAVDMRKVAALSAGAASVIAGAAIAFGATPAMAAETPASAPVNTNDPAKEQSVIANKNKTEGEDSKKTATETNKKEESKKEQPEQTKPTEQTVVKTEGKADATKTESGSKVESATAVDNKVTNSTSATDKNNADSSTTEETSTPAENVVKKRTRRATVENKQPEATNYAKDENQKQSEPVVGQDREANPAPAPQVNSEITLSKETKDTLPNLYAWGSSDNVYIEKGQNQEVTFNFAKPSDGSTITKVAIFPSDGNTVDNDKSRKFLEYYSANENEHKPYSGKYEFIVNTDGSAKLTMTKLYSEANMAAEKYTANRCIYVYGTKDGKESVLYKTNIVRAATLVPPKTAGSIVLKYNEELTAQQIQDKLKAALDAPTEATGKKSIRAQIDAASRSNGVGGRTGENGKFVQTPDDPDKKVVITDNQAYDANQFNRINTVTSKNTDKVTTYTTGAQTLKTYLVSDLGYKSEALALTVARYDTRIDKPIVDEVDITKLTDDQKANIRKKLAQLNHVSQDKVTFNDQGEAVISFDGVDAADAPKIALKDLVMKKLAETDVAVPTGDKAVFVANPLGYSNAELDRIKTAIYEANKDNQELGLSKDNYKDQITLSYITGDLTCAGDANKGRSNGLQENNISVTIKTDKAVAEFTSDVKSNKLTRLPDIRTDYNVELVKNKLDGRDSDEGFSWSDDKHTTLIYRYDSTKAQAFTAPEILKLIKATPKDQKTGLRPLTGGEALDHEGANGKARKSHVYYSIDKNGEPTTELTLGMMSGPYWIGNPQVANSDVNMGDEESKVGQYTWDTEAGSVTVAAKQNKVFKTRLFVAPYTLTYYRGVYTNPYQKDPNNTPKAINIIFVPQTNHKKDDLSKSIAEHKTDKVEGKEVPTQSKYYNASDKVKKDYEDALKVAKQTLEKVGTTPDDQLTEQLKAEVDNATIKLDKARKALDGDATKKDELDKSITEDGTPAEGQQATTGTKASDKYKNVSNPDFKTADGKPDETRNNAAKEAKKAYDKALEDANKVKADDNATQKAVDDAKAKLDEARKKLNDFTTNKDELNNAIAQHGHVNTGDATKQGDEKLKTADPTYQNSTPEQRTAYDNAVKKAGEVVADPNASQKEVNDAIKKLKDAKDALDANATDKAPLDAAVQKTLDNPDPKDPNKHSVFYTNAAAKKDSDPAAKKAVEDYDKALAEAKRVLGEKNATKADVEKAKKDLEDAEKVLYADTYQTKTTDLAEALADNFSGYLMPAYFNAFDKAQAGDEQAKKDFKAYNDAYHAAKDLMEDLKKPGSTVDQEKVDVVKNQLIEARKVIDKYATDTSKISAALLHSLAITNSPAYKNASANTDPNSDEAKAKKAYDDALKELQKAFNDQMDKDRGDDGNEIPESVIPKKGGDTNSASYLDGIQSHAKQQPLNRDVTRLLEKLNDAVKGLDKFATKTDDLIKSINEDATTHPSPAFKNASHPDYKQEDGTTADKTKNDAAKKAIDEYGDALNAAKDLLKNPAATQKQVNDALKTLNEKRAALNDYNTDTTKLEKSVGEHGKEKQGDTAATEGTVTSDAYRNASDPHFMKEEGGKLVPDKEKNTKAVEAKKAYDEALTKAQELLKKHDSADTPQDAKPTQTEINAALDALDKARTEVEKYKTVTTDLETEINKSTADGAAQPTAGSFEESPEFKNADAKKGKGDKDNEDVKAYKEALKKARNLVKAATEAGKKNSERPTQQQVDEALEALKNAKKQITDNYKTNAAALKAAKDFAAGDFKNTPEYKNAKALKDNQNADADKKSKAKTDVDALDKNTNDGALNKAIGILQAFDDDGKPNVTTGARIPTQKEVDDALKTLQEAMKTVAEGYKTDVKPLSNEVGDKDTQGNPVTPPFEASVAYKNALEKAKTEDHATTDPNSATKKLEAYNEKLKAAQELINKVNNPDPNAKPEDRPTQAQVDKALQDLKDAKTAIDNAFKTNASSLKKEADDKDETGQEHNPKFEQTTEYLNALAKKTGDEDIPDVKAYKDALKKAQDLLKKFNDDGSAPKQGEKDIPTQKEVDEALKNLKDIKDKITKNYVTSPHDLQEEVDKSKDGDTDTSTDVFENTPEFKNADAKKGEDGKSDNSDMKAYKDALEKAKNLLDAFDRTTGKVKDQLPKGMTKAPTQKELDDALDALQAAKKKITDGYKTDPSKLKSEADANGDFTKTPEYQNAQAKGDDASKQALEDYKKALEDANKVLGDKDATQAQVDDALKKLQDAKSKLSDGYKTDKSDLTVEADKDSDFTKTPEYQNAAGSPEADAYKQALEDANKVLGDKNATQAQVDDALKKLQDAKKKLADSHKTDKSDLNTEADNDPDFRKSIPFIIGKAADLAEYQQALNDANSVLNDPNATQAQVDQALRRLRDAKQKLIDAYNRLVNTGVGVNDVNNTSVNNVVDKGALQAEVDAALGDVSANANGVVADSNLVSEFNAALNYARLVLADSNATQGQVDSALARLRAARAALRAGMLAARNSAGMNLKRGDVSGVNTGASSSVFAALAAVFAGLGVVGAASKRRKHSAR

**Schematic representation: SP-out-↓-in**

>lcl|AP012332.1_prot_BAQ32803.1_151 [gene=GAVG_0151] [protein=dipeptide/oligopeptide ABC transporter substrate binding component]

**23 unique peptides were identified:**

KVSDKPLVVQYDLNPK

VSDKPLVVQYDLNPK

TYDYKEQTALANK

IEQPGSELLSMFQAGYK

AVVQAFDVATYNK

NNLPADAKFNVENAK

AKWNDGTDIDYTAFK

KAVVQAFDVATYNK

TLEAAGYK

VKYNPDYITSVKK

VKYNPDYITSVK

IQFQGMSWK

FNVENAK

KIDAVLAK

YDNPQPR

VSDKPLVVQYDLNPKAK

ATWEVMNGK

YNPDYITSVKK

VDKRYDNPQPR

NNLPADAKFNVENAKK

NPKWWGK

YNPDYITSVK

SDSNFTGTGSK

MKKTNAGKLTVFAAAALSVAMLLGA**C**GGTTATTADKAKGGMTEEPAAGVDTSYTGALPMPKVDKRYDNPQPRDNVKDGGTYTFSLSDMGPNWNYASNDGNTAYMSTLWGFYQPSLPYYDTVKGEKVKYNPDYITSVKKVSDKPLVVQYDLNPKAKWNDGTDIDYTAFKATWEVMNGKNEAYSVPSHEGYDCIESVEQGANPKQVIVKYNKPCATWELLFAPLVHPKATDPTVFNQGWVNNPHNEWGAGPFEIQSATEDQVVFVRNPKWWGKKAKLDKVVVKRMEDTAAMNAFQNGEIDSVDSISTKDKIKAARSVKGAQLRYGYSTKIRVINLNAKAGAFKDKAVRKAVVQAFDVATYNKIQFQGMSWKIEQPGSELLSMFQAGYKNNLPADAKFNVENAKKTLEAAGYKMGKDGYYAKNGKTVQFTFTFFGDDSTQAALANAFQAMMKKAGMKCKTVNNPASKFSKTVSSFDFQMLPMAWVSQSPLSFLASASQVYGSKSDSNFTGTGSKKIDAVLAKIGKTYDYKEQTALANKGESMAFAEYGTLPVSAPPTYQAYKKGFANGGPAGYANVFVENIGWQK

**Schematic representation: SP/↑-out**

>lcl|AP012332.1_prot_BAQ33548.1_896 [gene=GAVG_0896] [protein=alanyl-tRNA synthase]

**22 unique peptides were identified:**

TLEKGTTILDLAVEK

IGMVTILSESSIGSGVR

SNVLAIVEAGK

SVSAPANVEVILDR

MLGVQDPVLPTLFPVSK

LGAMHLFGEK

IDEALDAVKSEVAKLD

AVDKDALLR

GTTILDLAVEK

ALREELGPQATQR

HNVDVSVYDDIKK

VLGGGGGGKPDFAQGGGTDASK

TDLHIVGELDQK

IDEALDAVK

NIDTGAGLER

FDFQWPK

TLDIDEVGK

SALMIMSDGVRPSNVGR

IGMVTILSESSIGSGVRR

NVGNFGSFDALR

VVEIGDGWSR

NVGNFGSFDALRK

MRTAEIAKRYLDYFGNHGHMVVPSASLISPNPTTLFTIAGMVPFIPYLLGEQTPPSRRMTSNQKCVRTLDIDEVGKTTRHGTFFQMVGNFSFGDYFKEEAIHYAWELLTTPQDKGGYGFDPEKLWVTTYTDDDEARAIWKNEGFDPEHMQVFGMEDNFWTTGGPGPGGPCSEIYVDRGPKYGNDGGPAADESRFIEIWDLVFENFQVDNVKSKTDLHIVGELDQKNIDTGAGLERLAYLMQGKENIYETDEVYPVIEAAEKLSGVKYGDNNDADVRFRVVADHVRSALMIMSDGVRPSNVGRGYVLRRLLRRTVRAMRMLGVQDPVLPTLFPVSKAAMEPSYPELNNTFHEVSEAAYGEEDAFRRTLEKGTTILDLAVEKAKKEHKDDPIVGGSDAFTLHDTYGFPIELTLEMAAEQGVKVDEAKFRELMSEQKGRARADALKKRHNVDVSVYDDIKKELEKPIDFLGYTDFSSRSNVLAIVEAGKGAVKSVSAPANVEVILDRTPFYAERGGQIADQGEIISDDGAILEVDDVQRPIKDLIVHSCRLTEGTLSVGAPVNTNIDQVRRAALARSHTATHTLHKALREELGPQATQRGSVVDPDRLRFDFQWPKAVDKDALLRVEARVNERIRDDLQVVPKEMPIDDAFKLGAMHLFGEKYGDIVRVVEIGDGWSRELCGGTHAKSTGKIGMVTILSESSIGSGVRRIDALCGEEAYEYGAREHALVAQLAGMVNARPDELAERLESLLNKMKDSDRKLSSVYESQLNDAVPELVEAAKQSGATVKLAVKNVGNFGSFDALRKTTLDVRSRLGEDTPVIVALCGVNEEDRPMVAVATNDAARDLGAKAGDLVRTASKVLGGGGGGKPDFAQGGGTDASKIDEALDAVKSEVAKLD

**Schematic representation: in-↑-out**

>lcl|AP012332.1_prot_BAQ33427.1_775 [gene=GAVG_0775] [protein=putative cell surface protein]

**21 unique peptides were identified:**

LLTADSSSNSAPIQVIIR

DSGEYFVTNVDSSSPSNR

TSFNPDSFDPSYVNSVAK

QDTSSSEIAKPVK

HGLLNTGATFDR

KLENFVDNDNSDAR

GKQDTSSSEIAKPVK

QSAQPETSQPASAK

VENSVKPADTKPADTK

SFDTNNFEDR

ASYTKPAAAPAAK

DEKTNPAETGKVAEKPK

SAASSADSNADNSATKTATK

HGLLNTGATFDRTTR

GAGNGNGKGDGKGDGNGLYK

TNPAETGKVAEKPK

TTEKPAVAKPK

DYDADGYHYVFK

LAIVAETLGK

FILENYTK

AEPNQNSAQTEHAENK

MARTKNSGSDVLSIKSAKHKSVSGDASNAKSVKSVKKNAKTAKRSSSSTATSASGHRLGVAAVALASTLSMVLPGAAALARTSFNPDSFDPSYVNSVAKTSGKGAGNGNGKGDGKGDGNGLYKHGLLNTGATFDRTTRQMVGSAKEYSVKNTDSNSIAEGVENQETVAGDSSVGVKDSGEYFVTNVDSSSPSNRSFDTNNFEDRSAASSADSNADNSATKTATKGKQDTSSSEIAKPVKSAKKSDTTQTKDEKTNPAETGKVAEKPKSNASVPAHKTTEKPAVAKPKVENSVKPADTKPADTKASYTKPAAAPAAKQSAQPETSQPASAKTVAKTKPEAKAEPNQNSAQTEHAENKQPATEQNTSAQPSSTHEENANTVNDENKPKVRSRRSVDESANGSNKGSSVTPASNAPETLSASAPSAGTESSSGAGNTASPSAEGNGNAQGGQGAQGQSAENPTAQGTAQGSHDNAQGAVTGTGVTGKDKSATPSASDTTPSPDSTPAGTQNTGTTTNNAATDPNSQVIKPNTDNPEAKSTEQANEQVQTDKKPKATYNLQIRYTIGGAANKQLVQPYELTIDKAGFDNLGKDGKYEYIELPKSAGYRPSVYHSGTYQYYIKNDKNEFVIDDGTNADAVRYLRLDKNLIKEYAVKKRQAVGGNNGAQAPQASSSQTTTNPQSSSSTQMPAEDGIQYYGELNINYAPKTAKYYVRHLVQDLDNKDKFIDAPNLGIGKVITITHKDGTSENIHVTEITGTVGSDVTAVSTYIPGYEPEHNLISSPLSDSEDEKDKLVLNLRYYRKAYEVTYDSAGGTDVTAQKVYYQQDVPQVKEPTRRGYTFKGWSLVDPNQKSDPLYDENTIVSLDDYKMPDHNVQFRANWEANNTTSYRVNVWVQKADLVDKDNPNSLKNYDFVGLVERKNVKTDSEVALNKMDDAGVANDTNKLNGENEDNYVENPELGLTKEELQGKDSDHKTGLISKFNWMNDTPVTSLDGYNTANPGAAENKGKDIFTRYFHVNKELTKKLNSEEHDFVPGRPDLGKRSKSQLCADDLNNTLNLVYDRNTYELIFAKPADINNGRLNNAAIKREDEHGNKTIYCYAGGGNCSDEYDDKNEDDSGNAINHKGYRVNVRYGQKLTDIWPDINELDFNEENIGSLGWTLGYAGDGRRCYRDTPPYRFTIKEFADPSLRVDFKNGAGIAPRISDDPSNPSAHQYELKDNQRLLTADSSSNSAPIQVIIRKQSIASAKNGGDDIGDNDYELSTDSYSKDDTDNSGYLYTAPSIAGFNPANGYDKTKVGDSLDSFDFEDNREEWYNESHGHGNWDDLSDGEKNAFAKKYHLAFRKYRGVPVDEQEDYSGDATEFETNKLLEFRYNRKSYDVQFYNADGKAIKDGNGSAKESLPFEYSLTKRGKKDLTGEDKDLYGNDTSYDASVTKDDAGNTSKQFDGKYTFTLNNKTYSIVRPADLPEDYVFKGWAVDQAGTQFINGENKDITMPVNGIKLYAAWGKPTNIKHTVTLDYHMPGTDENGNTIQDVVKKKEFARYNVIDEKNDIKVPTRKGYDFYGWEITKNGKTLPYAFGNKVVEDIKLDAVWVRDTRYNGTFKHIFLKPGYTFADYKKEGLSDAEKAAMVDHISTQTVSGLREHLRYNAEAVYSDETHFPDKHFTSFEASSDEKQNTGEFIYQTYNTRKYKVKYIDQNGKELLPESEVSSVNRNYDVAFYKPIEGFMPETTQKNIIYTTDSEGKQTNGIPTITFKYKDVRVLKRQDDNQYRPTNYTRYVFKVDDKQGSMGSVVDWQNNNVADGSALVYDAIKGTKAYQMPLPTVKAKQGYEFDGWTSQIGSYDAGSTKLQYADGVSRLPIRSEEQNSPEVIYTAKFKLKAPVAAAPQVLKPTENISIANSDDAKKLITNAGDYPDGAKFSFADGEKFDNTPGLHKIKVQVKLGDNSAEAEVLYRVLPDLVYASDWDKFKATDYGKAHIDEYAPITFTGKNDEGTIVGHDGDNANHPAGGETTLTAYVYKGKEVRIRVPQAFGKDHGQDNYYYVFKGWATKVVEPTAENPNPEQKYDIDPEGRYKDVTVDDNSGKTYTAVYKKIEYFSSTSDGGEVPKDAVVAIFKPAPGRLWKDGTSGPKVFYVKKGTDLEKITKEGSGALAWLNNQLTGAKGTWSRSSMLNDGKKVDSVPNVAESANKWKVDEPFQEFVADQTPWTEPTVQTDYLVAVQDKPDTLPKLTDFITNMPQLKADAATNNGVEDIKVEYDLPTEAEQNTLKQKMLKKPALYTVPLKVTVKYKDAADYKTYRLVGRLKVLYQLMYDKSLPKPDSTQPSHQPGDTNPLTGVAAERDLVLNKDKYVKVNFINANQVDHKDQGSLDKDTTKLYYVLKDDVTGVKAPQAVGKDYDADGYHYVFKGWRKLENFVDNDNSDARSRARRSLANQPEIPVTTLMVSEPASATSVDVQNANGEKLLTNDEIRKLKYSENTTYQAVYEKVMNIIDAKSNEKIPDNYVPYVFLPAFGRKWDDGSYKPKVIYFKSDSTTYENDVNNKTNELKQQLKGFSKWEVYDAGNKPVPLTDTNKNRQIRVYVANQIADMPVNVSQFVKSVGDEVPAPDELVSGVDPNNLEIAGMHGAGVAYSSGANSVRIAKPGITTVRVRVETSDPNKKGAKIVRYENVPIQVLPNVIAERDLPSSNSQAGKFILENYTKVTYVAGNGGTMQSRVHTYWVRKDRISEINNYIPDVLANKGYIFKNWDEVSYSKHIDTIYNSADNHKSTDIERETLAKIAERYGMSFTANVIRKSKNSTFAAIKNILAEAINNENMSYEASRNRLAIVAETLGKSYTAKIIRNSQHSTIEALKNLLVQAGITDADIAMVFPESETIITANFEKMAPMKFKFSGSAREGILTNFRLQNMTEGDVNNTTVTVDGKTLTINELVAQGLTSVNGINANYAGTECKISGTPKIVDGKPKVELTFTTTDKYGREAEITVEIDVISESKPAPVPTPAPVPTPVPPAPAPAPSKPEEEPRQEEYPYIPMPEAPLSEVVPEQAAEPKQATEPEAVKDSAKQPSEALPQTGSDVTQSALIASLLASVGLAGFAAKHRRRKNEDNES

**Schematic representation: in-↑-out-↓-in**

>lcl|AP012332.1_prot_BAQ32792.1_140 [gene=GAVG_0140] [cna protein B-type domain-containing protein]

**22 unique peptides were identified:**

SVIENVSSETDNSTSK

GSDSSVDSDAIPGEPVTDR

IQETVATYAR

NSYVGNISLSAGEDKK

SKVSTVPIEIVIPASLSGR

DPNPEKDKPKDPDNKPK

DYNPVKPDTPKSPDPK

DEPKDPKPNTEPK

FKIPFGSKDYTLNGTK

KIMGNIGIEK

NGNDNNPIKLR

ILTVNANDFVVDAGSVANK

NSYVGNISLSAGEDK

GVNHSGNVLSNGDVVK

QALKNHHLTVFR

VAYENTEVFGDILDSK

VWFDANHNGIQDK

HIDAGAFK

IMGNIGIEK

DYNPVKPDTPK

KDEPKDPKPNTEPK

FIVPEAGDVFRPYLR

MKMKIVTASLLTATLLTASLSSSALA**Q**SSNDNAFTNRENIPTNKSSINNIDLIPAPTQHFGKPDGKLPEVRTSISYVDGDSTDNHADTNKTNKILRPGSKLTLRATAKFIVPEAGDVFRPYLRLAIPKILTVNANDFVVDAGSVANKVVQISDSKSFDNHNLGNYNLFDVYFNYNEWSSDFANEMPYLAAIKGVNHSGNVLSNGDVVKNNQTVSIEIPTVVDSNANTSDLVDLQNYKKGVKPLIQGRVSYMPFPTPIEYTSTIGGLKDFPNDKILQDNPCLVRSDLVTGWDGRDDYGFWFTGLSMETSGVDYDSRSTMVESQNGVWLPQLIPANIEHNQFVTIYRNGNDNNPIKLRFKIPFGSKDYTLNGTKQALKNHHLTVFRSPNNNAEARKIMGNIGIEKVAYENTEVFGDILDSKYFDGWGTVPEQSRPSELLISGEFNPDSTVFGPDNISNGIWMSGKASASIKSYYRALDCNDNYPDSTLKYPPIINKIQETVATYARSNRSKVSTVPIEIVIPASLSGRVWFDA**N**HNGIQDKGESSIIGAKVQLVKQYGDSTVIDINGNEVKPITTTSPDGYYEFTNLLPGSYVVKFTLPEGSEYFGFTYTHKGSDSSVDSDAIPGEPVTDRSVIENVSSETDNSTSKNSYVGNISLSAGEDKKHIDAGAFKKDEPKDPKPNTEPKNPQPKHEDPKDPKQNPNPKDPKKDKPKSKDPKPKDPNPEKDKPKDPDNKPKQNPEPKKTDPKDYNPVKPDTPKSPDPKKDDPKPKDKPNNPNYQDRDVPDTKPEDPKKPDNPTKPKDDKSDTPKEPESKEPKDPKPNDEPKKDEPEDPDPKDTNPKSENPKDNENPKNPESKPDDPKSDTPKDPDTDDTESNDPPQYEDSQPKEDSSGPNHSEIRESDNPIPNINNDLDSNNSQSNESSFDNNNADFKQEHVRDKLVNTGVSTVFTAVASVAMLALGATNKIKSYFCKGKHRQ

**Schematic representation: SP-out-↓-in**

First and last amino acid cloned in pET29a are indicated in orange.

>lcl|AP012332.1_prot_BAQ33816.1_1164 [gene=GAVG_1164] [protein=hypothetical protein]

**11 unique peptides were identified:**

LADDEAALAKLESK

AGAVTEAQVAALVAAANK

KLADDEAALAKLESK

LADDEAALAK

TADKAAAKGELAAK

KLADDEAALAK

ATIEQDKQEIK

LADDEAALAKLESKK

KKLADDEAALAK

DGKLNQLVK

NTPSKPETKPVVPGVHVK

MMNKKAIAAFAAGATLLAGFAMATPAMAESLIPMYNQNNGFAEVSDGTDASVLKATIEQDKQEIKAAEDWNDKVSSAYANANTNTATVTVDGVVPADSSQATHVLVLGKDGKLNQLVKAGAVTEAQVAALVAAANKAEAAVDAAKKKLADDEAALAKLESKKVTPKKDDKNTPSKPETKPVVPGVHVKTADKAAAKGELAAKGGNGHGKSGEKLGNAGVGVALTALAASMLAGMGAAVRKMRH

**Schematic representation: SP-out-↓-in**

>lcl|AP012332.1_prot_BAQ33606.1_954 [gene=GAVG_0954] [protein=conserved hypothetical protein]

**13 unique peptides were identified:**

IAEQNTASTESVDSK

KIAEQNTASTESVDSK

TTTNKTPATDKTNATSR

NASVQSVVKTDGK

VATDKNVAETQDNK

TTAENSAPAETTVK

VATDKNVAETQDNKSK

TTAENSAPAETTVKK

SGSSVADQLVAQSK

NASVQSVVK

NASVQSVVKTDGKK

QPETPASTVAQGEDR

ENLLGTEIDTVKR

MVSKSRKHANTVKLDAVVSKAIEKAVDMRKVAALSAGAAGVIAGAAIAFGATPAMAVEANVAPVNTNDSTKEQNLVAKKNAEAEGSKDSDKTATETTKKDDASKIEDSKKAEPSAAKNDQADKKVATDKNVAETQDNKSKTEQNLTSSKNASVQSVVKTDGKKQIENNAEQKKIAEQNTASTESVDSKTTTNKTPATDKTNATSRTTAENSAPAETTVKKSRSKRSIYENKQPETPASTVAQGEDRAGAEHNPDEVAFSKNLPNIYGWATPSNTFDENLEKQQVVYHLPKSADGKTVVRVVILPDSKDSINTDDPEAYKKIIEFDSARVDEMHQSYSGIYELKTNTDGSVDLVMKQPFRDGGISSGQGYCANRSIFLYYDKDNLQHDQTSNNFRVATLVPPKTAGSIVLKYDERLSADKVREVLHNAVNLPTEHKSGSSVADQLVAQSKSAGVGLRVDESTVNNTPDTVDAKIPQGMGSYDGAMFGEINKTNDSGDYSFGERKLKTYLVTDLGMKSPEIPLTVVRYSTRIEKPVVDTVDFSKLTEDQKTQIRKNLAKLNGVSTDKVTFDSSGTATIDFDGLSPQDDPKIPLSDLVLTRVSTDKVAIPSDSENSKVKAVVVANPLGYSQAELKQIKKAIYEANKDNQELGLSAKDYEKQISLGWLTGDTTASGGQNTGISNGMNENKITVTIRTDKAYAQFESDIQKHELTRLIDLRKDYTLSWDASNNKISGRTSDEGLAWMEEGKTLVYRYDPDKGEQINTQAVLGLLKATVKSDVKTDNPQLRENLLGTEIDTVKREGSNGQARRTHRSYTVDDKGEPIGVLNLVKLNGSSYGGFAKPVDNSNKKMGDEQSSVGDFTFDDDSKKVNVAGKTGKFMLGRLFIEPYSLYYYNYVYGENKYNLRNTPKGINVVFVPQTKNKKDDLSKSIGDHKLAADKKTPTESKYYNASAEKKDAYDKALDAAKKTLEKVGNKTDADLTEELKAEVDNATITLNKAREALDGDATKKEELNKSIEANGKAPEGATAATPGTVTTDKYKNVTDQAFQTDDGKPDTKKNEAAKAAKKAYDDALAEAEKVKADDNATQKAVDDAKAKLDAARKELDKYTTNKDKLNAAIAEHGKVNTGDADKQGDEKLKTADPTYQNSTSDERTAYDNAVKKANELSKDPNASQKDVNKAIEDLKKAKDALDKNATDKSPLDAAVQKSFDNPDPNDDSKQSVFYKNAKNKTNDTAAQQAVKNYDDALKKAKDVLANDKATKKDVEDAKKALEAAETALHSDKYSTDKTDLGKALADNFSGYLMPAYFNAFDKAQADGQDSQAAKDFKAYNDAYHAAKDLMTELNKPNSTVTQDEVNKVKDQLIAARKIIDTYATDTSKLSAAAFNDIAIKFSPAYQNLKALAEKENPSEAEKADVEAAKKAKKAYDDAAEKLHNAITNKLPKDQANGQDIPDSNIPKKDGDPNDKDYLKGIQAHKNGEPLNRDVDTILKEMNEAAKALDKFATKTDKLQESINKDTDTQHDPAYKNAKDPHKLGTDGNEDTSGYNDIKQKAKDYDDALTEAKKLLTNPTATQAQVDAALKKLDEKRDALKAQDTNVEALKKSVDKNGKDASGTEQAVEGTKDSDAYRNASDPHFLTADGKPDDTRNNAAKEAKKAYDKALAEAQELLSKHDDTNTPLDAKPTQKQIDDALTELNKKRKALDDYKTNVDALKTEAEKSKADTAQTVGENDFENTPEFKNADAKKGEGNKDNDDVTAYKDALKKARELVKAATDSGKKNSERPTQKQVDEALEALKKAKQTITDGYKTNLDALTAAKDFANGDFKKTPEYKNASALKDDSSDTQKQSKAKTDVGALDETTENSALKKAKDIIDNPTGKTQKEVDDALKTLQAAMDTVTNGYKTVVDPLEKEVGDKDATGKPITPPFEESIPYKNALYKKQSETGNDATADTSATKKLQDYNEKFKAAQDLINKVNNPDPNADSDKKPTNADVKTALEALQKAKKAIDDAFTTSAKDLKDESAKSTADGGTVADADFEATTEFKNADSKKAEDGKSDNADISAYKEALKKARTLLEKFGDDNKPKSDAKDVPTQKEVDEALKKLKEIKDKILADYKTSSTELQQEVDKSKDGDKDTRDDVFENTPAFKNATAKGDEDSKKALEDYNTKLKTARDLLKAFDRTTGKPKTKLPEGVTTVPTQKQLYDALDALQAAKKKITEGYKTDKSDLNTEAGKDSDFTKSPEYQNAAGSSEAEAYKRALDEANAVLKNPNATQAEVDEALKKLQDAKQKLTDSHKTDKSDLNTEADNDPDFRKSIPFIIGKAADLAEYQQALNDADSVLKDPNATQDQVNQALRRLRDAKQKLIDAYNRLINSGSGVGDNTGVGVNDSNTTSVNNVVDKSALQAEVDQALGDVSANASGVVADSNLVSEFNAALNYARLVLADANATQGQVDSALARLRAARAALREGMLAARNSAGVNLKRGDVS**GVNTG**ASSSVFAALAAVFAGLGVAGAASKRRKHSAR

**Schematic representation: SP-out-↓-in**

>lcl|AP012332.1_prot_BAQ32694.1_42 [gene=GAVG_0042] [protein=hypothetical protein]

**6 unique peptides were identified:**

AANDAQTAAYADDTDGAK

AQKELSEAQEKLEK

LEAAVAEAEATVAEK

ELSEAQEKLEK

NPQGTKPVEASK

AANDAQTAAYADDTDGAKK

MMNKKAIAAFAAGATLLAGFAMATPAMAFDFNVGLNDDGSWGTTQKKAAKTYTKAEILKLEAAVAEAEATVAEKTKAANDAQTAAYADDTDGAKKKAAVEKAQKELSEAQEKLEKAQKELNDAKPAPSEADPKNPQGTKPVEASKDATVQPGKPGAKPGQAAGQAGANAAAGAKTTVVEKKKDGGKK**LPTTG**VGVALTALAASMLAGMGAVVRKARH

**Schematic representation: SP-out-↓-in**

>lcl|AP012332.1_prot_BAQ33075.1_423 [gene=GAVG_0423] [protein=phosphate acetyltransferase]

**8 unique peptides were identified:**

GPDVDLVEEATR

ASFQSMSDEEVLSK

DIVNIIIVGER

AADYLLER

TIVLPEGEEDRILK

VGMLSYSTLGSGK

NVVALGVTK

ALSSHYPTTVFRPSAK

MSLVNVTIISADCAQSRNVVALGVTKALSSHYPTTVFRPSAKHDDAFTSELIASSNTKPTLEQVIAACPCAVRKNKDTLRGDIVAHFNELISVTNAQGCVIVASDSTSVFDPDLFRFDASVAADLASPVFLSICAEGRDSKQILQTIEAQCASVSKEYTKVLGVFVTDCKEDLAKEVKENYAQNNNGTPLWTLPSISESESESDKFNDYVADEEIISALKQPFNAPTTPYAFQYSLLGKAKANKKTIVLPEGEEDRILKAADYLLERDIVNIIIVGERESILARGQELGLKSLSKASFQSMSDEEVLSKMISKLCELRAKKGMTEEQAREQLKDASYFGTMLVVLGLADGLVSGSVNSTANTVRPALQVIKTKPGSKLVSGAFIMCFKDHVAVFADCAINLNPDAEQLANIALQSAQTAKAFGLDPKVGMLSYSTLGSGKGPDVDLVEEATRLVKEKDPDLKVVGSIQFDAAWSETVAASKAKGNDVAGHVNVFVFPDLCAGNIGYKAVQRSSGALAVGPILQGLNKPVNDLSRGALVQDIINTVALTALEAQF

**Unknown organization**

>lcl|AP012332.1_prot_BAQ32823.1_171 [gene=GAVG_0171] [protein=D-alanine-D-alanine ligase]

**4 unique peptides were identified:**

DVVLDPAKGADGFLVR

MEEGELPVITK

EAGEDQFYDFDSK

VVFLYGGK

MTKKRVVFLYGGKADEHSISCISAAGVLSAVDESRFEIVRIAITKQGEWIVGGEDPRNFRMEEGELPVITKTAETRDVVLDPAKGADGFLVREADGTLTSLGHIDAVFPVLHGPNGEDGTLQGLLEMMQVPYVGCGVLASAA**C**MDKYYAKQLFKAAGIDVAPGIALDVRKFASDAANHFDAYANEILEQVEEAKLEYPLFVKPSRAGSSFGVTKVESRDAKALADAVYEASKHDWRVLVEQGIDAREIECAVLAARDGDEPQASWPGEVVLDKREAGEDQFYDFDSKYMDSAASHVEVPANLPEETLQRVRNTALAAFKAADGRGLSRVDSFVTPEGHVMINEINTMPGFTPISMYPKAWEATGISYSDLITKLIEGVILD

**Unknown organization**

>lcl|AP012332.1_prot_BAQ32908.1_256 [gene=GAVG_0256] [protein=dTDP-4-dehydrorhamnose reductase/dTDP-4-keto-6-deoxyglucose-3,5-epimerase]

**2 unique peptides were identified:**

AAADSLVANVPQHYIIR

IEANRVDDYTR

MEFEKELSVTKTNIPGLIVFDLPVHGDNRGWFKENWQRAKMTALGLPDFGPVQNNISFNEKRGVTRGIHAEPWDKYISIATGEVFGAWVDLRPGKSFGQVYTTTLNPSKAIYVPRGVGNSFQALQDGTAYTYLVNAHWSLEQKKTYTFVNLADPDLHINWPIPLEESERSEADLHHPMLKNAKPMAPKRTLVTGCNGQLGRAIQNYAKENNLEGFEYVDLDSFDIANKDDYSRYDWDLYGTIINTAAYTSVDGAQTLQGRRAAWNSNVKGVANLAKIAQEHHITLVHISSDYVFDGTQENHKENEDFAPLGVYGETKAAADSLVANVPQHYIIRSSWIVGEGRNFVTRMIDFANRLKNGESVSVQAPIDQTGRLTFASDLVKGMFHLLNTQAQYGTYNLTGSGKIASWHDIAQKVFTQLNVDTSKIEANRVDDYTRISNGSPRPHKCALDLQKIESAGFTPCDWEDLLESYVNRIEQNNK

**Unknown organization**

>lcl|AP012332.1_prot_BAQ33209.1_557 [gene=GAVG_0557] [protein=L-lactate dehydrogenase]

**4 unique peptides were identified:**

VIGTGTLLDSAR

VLEQPITDPAEEK

EGGFTVFYGK

EATGLPYER

MRKVAVIGMGNVGAAVAHQLIIGGHVDDLYLYDSNEAKVKADALDFEDSMDNVPFNVNITVNDYEALKDVEVIVSALGHIKLLDVPHPDRFAELKYNRKEVAEVGAKIKASGFHGVLIDITNPCDAICQLYKEATGLPYERVIGTGTLLDSARLHRAVGKFFGVHPKAVKGYSLGEHGDSQFVAWSTVKVLEQPITDPAEEKNIDLDAVDDETREGGFTVFYGKKYTNYGIAAAAVRLVNAVMTDSREQMPVSNYREEYHSYLSYPAIVGRDGIIEQCKLDLTEEELQKLQHSADTILSKAQME

**Schematic representation: lipoSP-out**

>lcl|AP012332.1_prot_BAQ33222.1_570 [gene=GAVG_0570] [protein=leucyl-tRNA synthase]

**11 unique peptides were identified:**

GLHVGHPLGYLATDVVSR

TYAPDDANSEPEAPLSR

LALEAVADR

LIVLNNHLTSLPHVPR

LVEDLDTIDWPEKVK

GSARPIAELVDEFK

AKLEVDPNIDASELEK

SFATIDTNYVR

VQLDLGDGVK

SIAHADWPVEDSR

ALYDLGYVDSAEPFHR

MEDNDQQYSTADNKATIAEPAFRYNAQLAQDIEQRWQKTWDEQGTFWAANVKGDLKDGEGRLACGRPSFFAMDMFPYPSGKGLHVGHPLGYLATDVVSRYHRMKGENVLHAMGYDAFGLPAEQYAVQTGQHPRITTEANIANMRRQLHRMGLSFDDRRSFATIDTNYVRWTQWIFSKLYDAWYDPDFVRKDGGKGSARPIAELVDEFKSAKREIPGFADKKWGELNKVDQADVLNDFRIAYISKSPVNWCPGLGTVLANEEVTAEGKSERGNFPVFQRNLRQWSMRITAYAHRLVEDLDTIDWPEKVKLMQRNWIGESHGASVHFEVECADGETRDLEVYTTRPDTLFGTTFAVVSVDHPILEHVPEAWEASVPESWKGGYASLKEGLAEYQAQARAKTAKDRTEDAGAKTGLFTGLYAINPVTGAKLPLFVADYVLMGYGTGAIMAVPGGDQRDYDFAKAFGLSVIYTVKPLPDSVEKLEDYDGKAPFVSHDGIVINSSVEHTYKLGDALSINGLRVDEAIKKVTTWLEAAGVGVGKVSYRLRDWLFSRQRYWGEPFPIVYDEDGVAHLVPDSELPIALPDVPDYQPKTYAPDDANSEPEAPLSRNEDWVKVQLDLGDGVKTYYRDTNTMPNWAGS**C**WYYMRYLDPNDAEHMVDSDEYDYWMGTNRPGKEHVSGGVDLYVGGVEHAVLHLLYSRFWHKALYDLGYVDSAEPFHRLFNQGMIQAYAYTDERGQYVPADEVEEGANNGVGEPTFTWHGETVNREFGKMGKSLKNIVTPDYMYENYGADTFRLYEMSMGPLSESRPWNTRNVVGGMRFLQRLWRNVVNEETGACVVTEDALDEKTLKLLNNTIVEVTVEMEAMRPNTAIAKLIVLNNHLTSLPHVPRAAVEPLVLMLSPIAPHICEEMWSKLGHTKSIAHADWPVEDSRYVGADTVTAVVQIKGKVRAKLEVDPNIDASELEKLALEAVADRLGGKKPRKVIVKAPKIVSIVPDES

**Unknown organization**

>lcl|AP012332.1_prot_BAQ33277.1_625 [gene=GAVG_0625] [protein=methionyl-tRNA formyltransferase]

**3 unique peptides were identified:**

VLPILFAGTPEVSVAPLR

ALAQDKEHFDVR

AVLTRPDAPTGR

MVLPILFAGTPEVSVAPLRALAQDKEHFDVRAVLTRPDAPTGRGRKIVPSAVKKAAIELGIPVLEVNPSDEEECIRALKATGAKLAAVVAYGKILRQSVLDALPLGWYNLHFSLLPQWRGAAPVQRAIWAGDDITGATVFKITRGMDEGPILAQMTTEIGAHETAGDLLMRLSNDGADLLCSALVGMESGQIIPVEQDPTPCQIAQKITVEDAHIRFDIPAFAIDRQIRACTPNPGAWCNLHVDCGDLQIISLHVLSCALAKDEDISIHNLSELKPGQIFAGKRNVWVGSSSGVLELLEVKAQGKKAMKAADWARGAHLSQESYLD

**Unknown organization**

>lcl|AP012332.1_prot_BAQ33303.1_651 [gene=GAVG_0651] [protein=truncated hydrolase]

**2 unique peptides were identified:**

FVGPSILESFQR

YVFENIEFDPLRDR

MKVVLLDLDGTLTQSHAGIIA**C**AKKAMSDLGMQIPDDTEMLRFVGPSILESFQRNHMPKELQSEGVKLYRKYYSEVATFIDPDNPNGDMITGNFLNKVYDGIPEQLKLLRKSGYYLATASCKPEYQVKPICDHFGLTNLIDGIYGASKDMSRINKDQVIRYVFENIEFDPLRDRALMVGDRWTDVDGALACNIDCLGCSWGYAEPGELKTHGAYRIIDSVSDLNNAINEYFE

**Unknown organization**

>lcl|AP012332.1_prot_BAQ33363.1_711 [gene=GAVG_0711] [protein=phosphoribosylaminoimidazole carboxylase ATPase subunit]

**3 unique peptides were identified:**

LMPGATIGIIGGGQLGR

SLILEHPEWHVHDYGK

HLTAIPQGTDLLR

MPSLSEVTNGAVERLMPGATIGIIGGGQLGRMMAIAARHMGFRIGVLDPTLDCPVFQVADLQVEANYDDPEGLRELAERCDVLTYEFENVNADALDKVRHLTAIPQGTDLLRVTQDRVCEKEFINKHGIETAKWREVNNLDDLDAAIEEIGLPAILKTRRGGYDGHGQDVLRTEEDVANIHHRSDRGGKFPPSILEGFVDFAFEASILVSGNGNDFVTYPLVKNVHHNSILHMTLAPAVVDPVVEETAHDLALQLAKGFELAGTLGIELFITKDNRVVVNELAPRPHNSGHYTIEACDMDQFEAHIRGIVGWPLKKPKLLSPAVMVNVLGQHVAPTRSLILEHPEWHVHDYGKAEVRKNRKMGHITVLCDDPVKEAAELDATGCWDDELD

**Schematic representation: tatSP-out**

>lcl|AP012332.1_prot_BAQ33421.1_769 [gene=GAVG_0769] [protein=inosine-5'-monophosphate dehydrogenase]

**5 unique peptides were identified:**

GMGSLGAMAPR

FIASEDYDRLK

LLADNKVEKLPLVDAEGK

ENLVTGPSNISKEDAHR

LVGIITNR

MNTESVYAPVPPIFEKLGLAYDDVLLLPNETDVIPSEVDTTTHLTREITMKVPAISAAMDTVTESDMAIAMARNGGIGVLHRNLSIDDQAAQVDIVKRSESGMITDPLTVHPDATLADLDKLCGRFHISGLPVVDSENRLVGIITNRDMRFIASEDYDRLKVKDVMTRENLVTGPSNISKEDAHRLLADNKVEKLPLVDAEGKLTGLITVKDFVKTEQYPDATKDDQGRLRVAAGIGFLGDAWQRACALMEAGVDVLVVDTANGEARLALDMIRRIKADKAFDGVQIIGGNIATRQGAQAMIDAGVDAVKVGVGPGSICTTRVVAGVGVPQLTAVYDAAQACKAAGVPCIADGGIHYSGDIAKALVAGADTVMLGGTLAG**C**EEAPGEKVLLHGKQYKLYRGMGSLGAMAPRGKKSYSKDRYFQADVTSSDKVVPEGVEGEVPYRGPLNAVLYQLLGGLHQSMFYVGAHNIKEMQERGRFIRITDAGLRESHPHDIVMTAEAPNYSGFHN

**Unknown organization**

>lcl|AP012332.1_prot_BAQ33477.1_825 [gene=GAVG_0825] [protein=NADH oxidase]

**2 unique peptides were identified:**

LFQNAQTVIAK

DRVNYIALATNAVR

MTKIIQIGANHAGTA**C**ANTILSYPGNDLTIYDQNSNISFLCCGMALWIGKQIQSSDGLFYQKPEDFVKKGAKVNLESKVLDIDYAKKEVTVELKDGTVIHDSYDKLVLATGSLPNKPRIKGIDLENVQMVKLFQNAQTVIAKLQERDFNNVVVLGGGYIGVELAEAFKRLGKNVTLIDMEDHILNGYFDPEFSDNLKQIMEDKGIKFELGQAVEEIVGDTEVKAVKTSKGTYDADMVICAIGFHPNVALGKDHLKQYENGAFLVDKKQQTSDPDVYAIGDCATIYDNARDRVNYIALATNAVRSGLIAGHNVCGTPVETEGVQGSSAMMIYDYKLVCTGLSLTAAQKEGMDVDYVDFEDTQKPAFMEVENPKVKIRIVYKKDSKVIVGCQLASNYDMSAAIHLFSLAIQKKVTIKELALCDIFFMPHFNQPYNYITMAAYTALLKG

**Unknown organization**

>lcl|AP012332.1_prot_BAQ33722.1_1070 [gene=GAVG_1070] [protein=30S ribosomal protein S17]

**4 unique peptides were identified:**

TIAVELEQR

GYVVSEAMDK

RGYVVSEAMDK

LDSIIER

MAENQERNFRKV**RR**GYVVSEAMDKTIAVELEQRSTHPLYGKVVRSTRKVKAHDEHNEAHIGDLVSIMETRPLSKTKRWRLDSIIERAK

**Schematic representation: tatSP-out**

>lcl|AP012332.1_prot_BAQ33724.1_1072 [gene=GAVG_1072] [protein=50S ribosomal protein L16]

**3 unique peptides were identified:**

VMFEIGGVSEDVAR

GAPEFWIANIHPGR

AIDKLPMK

MLIPKRTKYRKQHRPTRSGMSKGGTAIAFGDFGIQALAPAYITNRQIEAARIAMTRYIKRGGRVWITVFPDRPLTKHPLGARMGSGKGAPEFWIANIHPGRVMFEIGGVSEDVAREALRRAIDKLPMKCRVIAREGGDI

**Schematic representation: tatSP-out**

>lcl|AP012332.1_prot_BAQ33820.1_1168 [gene=GAVG_1168] [protein=ATP synthase alpha subunit]

**4 unique peptides were identified:**

HVLIVFDDLSK

EAYPGDVFYLHSR

TGEVLSVPVGDAYLGR

AIDAMTPIGR

MPTQEVGYVVTAGDGIAHVAGLSG**C**MANELLTFENDTLGLAFNLDAHEIGVVILGDFTGIEEGQEVYRTGEVLSVPVGDAYLGRTVDPLGNPIDGLGAIECKERRILEAQAPDVIHRHPVDEPLSTGLKAIDAMTPIGRGQRQLIIGDRQTGKTAIAIDTIINQRTNWESGDPKKQVRCIYVAIGQKGSTIAAVRQSLEEAGAMKYTTIVASPASDSAGFKYIAPYTGSAIGQHWMYNGKHVLIVFDDLSKQAEAYRSISLLLRRPPGREAYPGDVFYLHSRLLERCAKLSDDMGGGSMTGLPIIETKANDVSAYIPTNVISITDGQIFLQSDLFNAGQRPAVDVGISVSRVGGAAQAKALKKVSGMLKISLARYKSLESFAMFASDLDAASKAQLTRGARLTELLKQKQFSPRSMEQEVVSVWAGTHGKLDDIPVKDVLRFESELLDYLDKGTDILQVIRDTEDFTKETESKLDAAIDDFRRMFKTSAGKPLIVKDSLPPAENPAPVEKEQLVAKPKSDNRESEGK

**Unknown organization**

>lcl|AP012332.1_prot_BAQ33051.1_399 [gene=GAVG_0399] [protein=conserved hypothetical protein]

**2 unique peptides were identified:**

QSEATAQNLRPLVPTDR

TWNPFVK

MTWNPFVKKSADNGAQNGNQSATNENSENKIENKAKTRTKNAPTPKQSEATAQNLRPLVPTDRKASAKAARARLREKENAQYDAMKNGDLAHMPKSEQLPWRVYIRDYVDARFNIGEFFVPFAIVIMLCIFLTQNFLSTKYIALYFALAIVLYAYLFAAIIDVIIMWRKLRKELVKRYGEVSVGKSSRSAMYACSRAIQMRRWRLPKPSTPKRGNWPK

**Schematic representation: in-↑-↓-in**

>lcl|AP012332.1_prot_BAQ33368.1_716 [gene=GAVG_0716] [protein=conserved hypothetical protein]

**3 unique peptides were identified:**

GIPSQYDQTNGR

GGMIDEYNLQEHSR

RGIPSQYDQTNGR

MANRKERRAKARQSRRGIPSQYDQTNGRGRGGMIDEYNLQEHSRRLQENGVDGWKPSSSVTEQEEQLTRNIKRAKAGDDTWNTVRKVIGVCSWAVLLLSAFAFLVIMWLPSQPMVLVITVAVLFALGVFGLFFSFSNSKQNPRLDQHGTAL

**Schematic representation: in-↑-↓-in**

>lcl|AP012332.1_prot_BAQ32664.1_12 [gene=GAVG_0012] [protein=putative phosphatase]

**2 unique peptides were identified:**

SSEKDVEKVETTR

SLAHIEQDSRPNDVK

MTQLDSDIDSRNSDTVYNSLNNKNTNRDKENNFNKASFIDKALFMYSTAVSDVGTVRSNNQDSAFAGERLASICDGMGGHAGGDTASTIAIRSLAHIEQDSRPNDVKAVSSMMETSIMAAHDAIVGKAKRERRLAGMGTTVTAVSLVNGYWVLAHLGDSRAYLLRDNHLIRMTCDHSYVQHLIDTGHITPEEARNHPQRNVVMRVLGDFDIDPRPDMAIRLAHPSDRWLLCSDGVCGVLEDSTIQEVMSSVSNQEECAQKLVSMALKAGSTDNATAVIADATLALDADAFDLPHQTPLIGGAASKDLESIADIVNKAVSSTPVLREGKNSPAQRAAALIQDGEKSSEKDVEKVETTRLVQPSHLRNEEELRSTDTNEIPIVKKKNGRISTDPRDPDVARAVKREQAEAHKANKIRKRWMRIGVAFAILVICSIVASGAYLVYSWTQKQYYIGKGNDRVVIYQGVPTNIFGLELSHQIEETDIRVKKLDKSWQDQLDEGISFSSLKEARGHAKLIRREMRAREIEKHSNKQKSAKSSLDDKDGNNSGVVNSDSDSGGKTS

**Schematic representation: out-↓-in**

>lcl|AP012332.1_prot_BAQ32781.1_129 [gene=GAVG_0129] [protein=putative penicillin-binding protein]

**2 unique peptides were identified:**

GSEGSYGSGLGSGESGQAER

RPDIHIVASVK

MVANSNGQNSHSSNTGRTVRSATSAEEKRRANARTGTARPSATRKPRQRRKHKRIRDTWRTKHPILFWALIIIFTPIILGTLVFAVMYVNTDIPQPDKIAMADKTKVYYADGKTEIGSFAEQNREIISCSALKPYVGNAIVASENRSFYKDGGIDFKGIGRAIIHNITSKGRWGGSTITQQYAERYYLGETKTYLGKLHEAILALKIAQTQDKNTVLCNYMNTIYLGRGAYGIQAAAKAYFGVEAKDLTLSQAAMLAGIIPAPSSWDPAIMPKEANMRFKRVLRIMREDKYITDAEYKSAKMPQTINQTKQNVYSGPQGYLLNMVRSELTQSGAFTKEDLDTGGYRIVTTIDKAKQDLMFSVASPAMGGRGIAPAGLQVGGMSVNPKDGSIISVYAGDDYLSKPLNNATQALYEPGSTMKPFALIGAIQAGTSLNTLFNGNSNLKFEGIDKPVNNYANTNWGTINLYKATANSVNTPFMSLQQKLGRRAVAHTAVTAGLDPKRINGENPFTVLGNDPVHVSEIARAYSTIANQGRRPDIHIVASVKNPDGKEFYRAPTSGRQVFSPADMALTTKALQGVVQYGTSTEARGVGKPIAGKSGTANDETACSFVGFTPSVVTVFAIWYPNAQGNPQPVPSFRGYPGGVGYSAHLFTKYMRHALTGMPTEKFPEAKDNGKIGGEDGKWGLGGNRRTSEYDYSDRASDDKEPYKENKDKVDRTENKPSDSAGAAAGDSSRGSEGSYGSGLGSGESGQAERNSSGAFDSPGSPYGYSGSETQEGAAGGD

**Schematic representation: tatSP-↑-out**

>lcl|AP012332.1_prot_BAQ32990.1_338 [gene=GAVG_0338] [protein=putative ABC transporter substrate binding component]

**9 unique peptides were identified:**

VVADLATDTGTALDGGK

VGIDVSIR

SLDSIETPDNQTIIFK

DSPIVPLIYSK

KGGTLTILSSESK

SPVASGPYK

ADLLWQHVDK

TRVVADLATDTGTALDGGK

ADLLWQHVDKEIMR

MQNTQNIQNYYQIIKKFNRGFALNTLRAIAVILVFSLVYAFSA**C**GTAVQSGQNLQKRLPNASVLNGNPKKGGTLTILSSESKMDFDPARSQGLPITSNSLVFRALTTWKVTPSGKTRVVADLATDTGTALDGGKTWKYTLKKGIKYQDGSPITSKDIKYGLERSFANSLTGGFGYHKALLEGAKDYAGPFSGKSLDSIETPDNQTIIFKLNAAFANWPWVASLAAFAPVPLNSGNAQDYGKSPVASGPYKIVENEVGKQIVMVRNINWDSSADSIRANYPDKIIWKFGVDPSVAAQSMIQGNSGADNAILADFVPPAQRAQAKASYKSSNLLVTSGDGALEYLAMNNRRIKDVKIRKAIQYAVDKQSYQRAKGGAVAGGFATTLITPGVSERKVYNLYSANPRGDVEKAKQLLKESGKSKLKFSLIARPDQAQVASSIQASLKRVGIDVSIRTVDSQIFTDMITGDSGDYDLALSKWQPDFPSAYANIGPLFDSSQIGGGNWNISRYSNPKVDSLIRQAVGVIDKSRADLLWQHVDKEIMRDSPIVPLIYSKNTYIHGSNVENFFVGSFPAYPNYARVSLRN

**Schematic representation: in-↑-out**

>lcl|AP012332.1_prot_BAQ32762.1_110 [gene=GAVG_0110] [protein=putative ABC transporter substrate binding component]

**10 unique peptides were identified:**

ALGDNYAAAQLPK

DNPAAVAQMNTIAK

ANAIGELSDDAAK

AGGWIQAMEK

TFGTALANKEVNDGNAAAK

AAADVYLFPNDQLGDLVK

NAVVAPPDAAK

FAAFLGSK

LTVWSSSEDQK

GFEEALKK

MTNMKKVLGAGLAIATMFGFAA**C**GSSTNAGSSNKANPVKLTVWSSSEDQKAGGWIQAMEKAFKKDHPEVTFKNAVVAPPDAAKTVKQDAKAAADVYLFPNDQLGDLVKANAIGELSDDAAKQVKEDNDDTIIQSVTAQDGKLYGVPYMGNTWFMYYDKSKFSDEDVKSLDKMLEKGKVAFDISNAWYLPGFYLDGNMTLFGQKSNDGKAGIKIGDKAAEITKYVAKFIQNKNFVMDGDGAGLAGIKNHTVDAYFSGSWDAGDVKKALGDNYAAAQLPKFKSEDGEHQMKSFAGSKAVAYNPNSKAPEMAAKFAAFLGSKESQEQMYKLHGDIPVAKSLSDLVKDNPAAVAQMNTIAKTSVLQPTVPEMGAFWDPMKTFGTALANKEVNDGNAAAKIADFQKGFEEALKK

**Schematic representation: SP-out**

>lcl|AP012332.1_prot_BAQ32876.1_224 [gene=GAVG_0224] [protein=putative sugar ABC transporter substrate binding component]

**3 unique peptides were identified:**

LLNPQDITK

DMESIIDSAYAK

GPFNIELFTGSPDDNNAK

MNIGKKAIALFVGIAVVAGLSA**C**SGSRGGASKNVSQGIEKGATIGVSMPTKSEERWNKDGNNLKKKLEDAGYKVILNFADDKPAQQNADIENMINNGAKVVVVAAKDGSAVGPAVEKAHDAGAKVIAYDRLIMNTKAVDYYATFQLEQTGILEANYIIDKLGLKNGAKGPFNIELFTGSPDDNNAKYFFKGAWDLLQPYFKSGALVSPSNHGGGVNKDFKVQDWQKISVQGWKAEQAQKDMESIIDSAYAKGQPLHAILSPYDGISTGVINAIESKRPDLKPGTDNWPIITGQDAMESAVSSIARGKQSQTVFKNVNKLAEAVYQMVLEIAKGKKVSGINGKFNNNKIDVPSKLLNPQDITKDNLTDLVKDGYITQERFDKLVH

**Schematic representation: SP-out**

>lcl|AP012332.1_prot_BAQ32818.1_166 [gene=GAVG_0166] [protein=putative ABC transporter substrate binding component]

**16 unique peptides were identified:**

SGQSLQSNAPSTATK

INVVTTTGVLR

SGKINVVTTTGVLR

KGVPNVQLAEESVK

DLEIPAVFLEPNLISR

GHADLAYDLAK

DVVVEVPTNALK

GVPNVQLAEESVK

ATVIKPNAYDMKR

LLDVDAYMR

IYQLPQAVLGK

HVHGDIDPHLWQSVK

ATVIKPNAYDMK

EIPAEQGYR

NLVTTHDAFAYLGK

TYADMMR

MKIVDLLGKKSVKRAVSAALAVLTALPLGA**C**GAPNNALRANSANNAKSGKINVVTTTGVLRDMVANVAGDAANVSAIVPDNADPHSYEPRLRSIRDVVYADVAFSNYMMLEEHGVITALDANLRKGVPNVQLAEESVKHAADIIPMVEDVSLDTIWLGLATERMAAQPSEEAHSAQDGASTNTQKRAEESPSAAAGELASLNNLNKTKTNSQHASQLEKSGQSLQSNAPSTATKEGRATVIKPNAYDMKRSDRIRLTATKVSGPGALFAYLTGTFGSVETYFNSANGISAEDHVDLPADAHTHLSWAFSKPGEYTLELHADMLNAQNEVVSSLGNTKMRFAVGVNAQKIAKKRGASVVLNKGHADLAYDLAKRGFVYRVDTLQEKRFTTPKRVYYAPRDVVVEVPTNALKEIPAEQGYRFLGAARQKIYQLPQAVLGKHVHGDIDPHLWQSVKNGIAYVRTIADRLCEVDPKNARKYRENADRYINKLLDVDAYMRKKVASIPKEKRNLVTTHDAFAYLGKTYGVNIAGFVTINPSSEPSVQDRRRLQQILRDLEIPAVFLEPNLISRSSVLKELAAQAHVKVCQIYGDAFDSKVRTYADMMRANADSLAKCLK

**Schematic representation: SP-out**

>lcl|AP012332.1_prot_BAQ32810.1_158 [gene=GAVG_0158] [protein=carbohydrate kinase]

**5 unique peptides were identified:**

DQLGYTLAEGDDFHPK

ESLGENTVVSSSALK

TYEQSQLQK

ESLGENTVVSSSALKR

SYREVLSK

MAIHIVVMGVAG**C**GKSTVSEAIRDQLGYTLAEGDDFHPKANIDKMSAGIALTDEDRWPWLDLINKWMVARESLGENTVVSSSALKRSYREVLSKNLNVYFLHLNGSHELIAQRLKDRKGHFMPPSLLPSQFAILEPLGKEENGTVISIEGSVEDMVHRAIKAVKTYEQSQLQKQS

**Schematic representation: SP-out**

>lcl|AP012332.1_prot_BAQ32957.1_305 [gene=GAVG_0305] [protein=hypothetical protein]

**4 unique peptides were identified:**

IVQALIDNTPHSR

DLDNQALFEFNRDNK

ADDIVHDGIDVEDIK

SGLSAAYASVSDVK

MSLMRKTKHVLMNKLKRVTCLCAAASISLLAVASLSG**C**EPNNHAVGDTGKADDIVHDGIDVEDIKLLIVGSKSASLDSKLLHLCEKSGLSAAYASVSDVKNANEAARDAIKSASEQPVSMILINNINIDASDSTDLGVKSNAGGAHVDLKARESWIDAIKYARFAGIPVALVNPKNPPKDSTLYAAKLYIFKDLDNQALFEFNRDNKSNVDKSGKIQHSSLLKIVQALIDNTPHSRDVIINDKN

**Schematic representation: SP-out**

>lcl|AP012332.1_prot_BAQ32680.1_28 [gene=GAVG_0028] [protein=phosphoenolpyruvate carboxylase]

**2 unique peptides were identified:**

SPYVDALSVIQSLALHK

YGNPILAIR

MTTQNQQVTPADATIVTSGLGRKGPEERELPASLKQDMDLCLHILRDVLGEFDEKLLTVFDEVRLNAVNASTAHFAQMVDAQGTEDDGLNKAVEKINGMDLPDMQLLARAFATYFHLANLCEENYRVSVLHQRECEVEESNPVDPVNELTCAYHKLLTEMSPAQAKELLNKLEFHPVFTAHPTEARRKAVAGKIRRISKLLSKHNRLGGSDKRENYRRLYNEIDALLRTSPIALKKPTPVEEADTILDIFDATLFNTIPLVYRRFDDWLLGKKAGLVPPRCPAFFHPGSWIGSDRDGNPNVTAKVSRQVASKFSNHVLKALEERTREVGKCLTMETRTTPPSVELLELWNRQREMSEQLTENASDVSNSEPHRAVMLVMADRLHHTISRNTDLMYHSCEEFIEDLRIVQRSLAEAGDARAAYGPLQDLIWQAQTFGFHMVEMEFRQHSLVHSRALEDIREHGLHGERGPLQPMTREVLDTFRALGAIQKRNGMRAARRYIISFAKSAQHVRDVFELNRLAFAHPQDAPTIDVIPLFEQLEDLQNSVNVLEEIIKIPEVQARLKATGRKMEVMLGYSDSSKDAGPTSATLALHSAQERIAQWAKDHDIDLTLFHGRGGAVGRGGGPANRAVLAQPVGSVNCRFKLTEQGEVIFARYGNPILAIRHLESVAAATLLQSAPSVEKTNTEMTHKYADMAQKLDVAAHNRFIDLLQTDDFTPWFSTVTPLSEIGLLPIGSRPAKRGLGAKSLDDLRTIPWVFSWAQARINLAAWYGLGTA**C**EKFGDLETLRRAYEEWPLFSTFIDNIEMSLAKTDERIARMYLSLGDREDLSDKVLSEMQLTRKWVLQIVGDKWPLQHRHVLGQAIRVRSPYVDALSVIQSLALHKLRNKVDKEELSESQQAEFIYLILCTVSGVAAGLQNTG

**Unknown organization**

>lcl|AP012332.1_prot_BAQ33288.1_636 [gene=GAVG_0636] [protein=long-chain-fatty acid CoA ligase]

**5 unique peptides were identified:**

FGEDLPTQVNVK

QAIDQAATHLKR

QAQETVKQAQEAVTPR

QAIDQAATHLK

VKQAIDQAATHLK

MLREFAVEVIHPTNDGDTIYSLLANRAMRDPQGVIAQWQDDDTRQWHDVTASEMLLKVRATAKGLIALGVERGSKVVIYSPTCYEW**G**VADFACACIGAVTIPIYETDSAIQAAEIISEVKPVIAFAGDDEHAQCLERVRQDNKRMSLKTIFNFKAAGIDAVIDFGKSISDDELNKSICQVKADDLATIVYTSGSTGKPKGAMLSNRNFTHIVYAAYDVLNDMLYKPSRLLLFLPLAHCFARYIQYVAIGSHGVIGYVPNAKHLLADLRSFKPTYLLGVPRVFEKVYNAASQKAGAGIRGRIFAKAVQHFIEWSKDEVEGRTHSIYAKLQHDFYMKTVGSSIRSALGPNLSWLA**C**GGAPINAELAHFFNGLDGITFIQGYGMTETAAPCVVNFEHANQVGSVGRPGPGITIRIADDGEVLVKGPNVFIGYYDKPDLTSDVIDEDGWLHSGDLGEIDDEGFLSITGRKKDIIITAGGKNISPAPLESIISKCPLVSHAVVVGDGKPFVSALIEVEPDMVRSWLKSQGMDENLSMSAVAENDAIRSVVQQFIDQANSTVSRAESIRKFIILDEEFSQETGTLTPSMKVVRPKVLKRYCDLIENQLYAPKPNSRPLPATVKILDKTTETVKQAQETVKQAQEAVTPRVKQAIDQAATHLKRFGEDLPTQVNVKDQHTEYAEKNNNSNDKE

**Unknown organization**

>lcl|AP012332.1_prot_BAQ33408.1_756 [gene=GAVG_0756] [protein=conserved hypothetical protein]

**10 unique peptides were identified:**

LAAIETEIQK

STTTEQFVGK

IKQTGEDFDDAK

LQSAQKDKLDLEEK

QLESQKNSLTSQDAR

GAAGSDPTYGHVAVVER

NAGNYEYVHY

AKADSEAQSAQAAAK

EAAEIIALK

TPHVGAVMVFAR

MESKKCHAKYCKRTVASAIASSFLMLSAGITLYA**C**QPLRADAVTMQDYNRKVQSNAALKKRLAGVNKQLADKILELNDLNEHQIPNQVRAAQQAQEQAQQAMGLVESTNQRLQSAQKDKLDLEEKIKQTGEDFDDAKAAVAQLARKSFHGSNASNVMAMVTKSTTTEQFVGKLQSEAAVARSEANAANDAAVTLSNSMNRRQRLAAIETEIQKLKAKADSEAQSAQAAAKAASEKQRSLQSLRDQGTILRKQLESQKNSLTSQDAREAAEIIALKSQIDAQARALAATAAARMNPNNGNRQQLRGGGSWNTPSLPSLPLSNGAANGMNYAVPGNCPAGSGNCYGHNTGNTAGGSAYPARQCTLWAYLRRSQLGLPVGSFMGNGADWANSGRRLGYLVNRTPHVGAVMVFARGAAGSDPTYGHVAVVERVNSDGSVLISEGGVGFATFPSYRTIRNAGNYEYVHY

**Schematic representation: SP-out**

>lcl|AP012332.1_prot_BAQ33652.1_1000 [gene=**GAVG_1000**] [protein=hypothetical protein]

**3 unique peptides were identified:**

GDASDKGLIDSAMMSAAK

NGVSVNNNLANNAAK

GLIDSAMMSAAK

MRSIWTVLLSVVVWIGRLAQQATKAALIFLISVAMWLPMCLTANTAVAADSGTAANAAISGNSGKKHESNKSGDKPKSAAKPNAADNADISAPSQPPVFIDGQNNAFAKLKNGVSVNNNLANNAAKGDASDKGLIDSAMMSAAKGMAKQGASYTSFGMDRSNEIFNNTERSSNGDKMGYDRSRMEFRPGCNRNYARSECITDDGDETGHRASKGADMERGDNPWREDSGYDQPQGIYTNNDPNFTPYPRGTYLQKRIRNLAYLKNKNGVDSLNRIQLDDVHDYIFMKRTPKRDGNGYIWDVLVNMGARIHGAAKALTYFVVPKDQSLDTSNGQYDQYVERLHFDAKNRGCAKDWVNPNGFCIDEGTRKEPLNDGETLDKAWYRIGNSNAIENVGVFEGGTQRDRDDSNRKLGLCDSYGFDCYHSGNYGFPSEGEHKAGTRLPPDGTQKFNDTYIKLIDNLFSELRGTDKEPRAKIFALRNQFDESKSPYSYHIHYTTSNNGKYGKLSTSYYGAGSYYGDTWHGPLSRYGLGDPYSRNPKYSLSNYRIGSGHTTYEYTTLYQQWYGIPNLVDINVPHYSFLRGTHLGPFTSNNGQLLAHTKNIDVTSLLFQPTRQEVCDKDTEAQCGNNTVWAKGQLSLLPKYNASENKYARGNRWWRKPLDPNTNINTKDSKFGIHDLTVQYWNDKLQMGSAKKVKYDIIGQADVFRPLQTQEWQDNPKYRSSKDSLGKAADYVNYLDNKGKIYEFAHALNPRLYMPDSHSSNNLENYSKYSDSDDSVYYGSIKDGKIPGDAHGLEYPLNDSYLYSQDSGANAAIKDRAIYSVEWTNADGSLKKSNTLKDAETKVAVRVPVVNVVDPCKGDDDKAADKAADKSAQADAAAKNKDSQECKLRPLNDTKRYIWKLYDAPSGKTVDQKLTGDEKAKYAKLAKDYFSNPVKEEYATPTNPNDENRIMLQFGEQPEVKPVYVAAKYAKITYWDESTGVLPLVFTHVDDEKPTIDVKVSINGGEPRSVPENGMKISVGSRIRFLVTGHDDKHVYMGTKEKVKDNNNKTDIQSTSEQFKATAFNKYDQSKPVGVDSATDQSGYVTTSETNGPELKSNDSGVKEFTFHAWDDAGNKVQKKIKLIITGDEFTAPNVRWQKQPNGRYVGKVNLLTKSKQVAVLAVRIWKRGEPKQKPVGETDTFRIFSDCPNKSCHGIMLTRQAGEDWQQLNDDKPDHSYFTNFMPYEKDITFDPKTGEITIPEHLAEIGSRIYAGVGNNASQLQTTLNASSDPLPLDMTWPDDGMVQVNPYELNPSEKQGLIDRIKQKNPRLFQYRKDEIGWSKDETKTLGGTSDKYSCKDNTKQYKICLSVTPDTKNTAQGKSGSEKTFKVVKATGTGNDTKDDQNQETVLDPKQKKITRFVNIRADYDWSYGSGKINGRNTDDGFKWYGRDENSSQYLVYRFNINQGDNKNFNTNEALSLFQGTKKSRWLRNQIQPSLYPLNTNDADSTNKTTIEKLWGGYNRMPNRGDGIGYARKVNNARGEWANIVDLVNYSNLGGGQTIFTNNITDKDNFVQPQGYVMPGGEDYNQQDSGANIGAAILKKSGNKYPLHAQLYIFNGNPWRELEARDEGDKDHTPNVINVWFVPVDKNKPWISVKDANDPSKKLLGECNPTKTNANSCGTVTTIDMNKTDITKQSGLFNVVDLLALDDDFNVADKTTHVSKALKDTLSIDIEAEGATDPKTKKAPRVRFVTGGNTNKELLRGFIEKWRGNDSKNSPTFKMIAQVTDDSGNKSDEAVVGKFKFNWTVAKAPVVRAYDGNHYNDLSGSGVWLHDWEGASAPNYSNKVSVISGNNATRLVVYFARAKKAAAKTGNTVSLRNAGANAIYSRSADATTGINDDSDISESLALCRAKTGDRWSLCDGYMFPEGLSTNDLNQTAFGGDKTAILFPSGFLAPGSVVRARNRTGSYGPWSDMPGVKSENLDELANDANETTSVNNDLKKLSGSENVSQAQNGANSAKEEDMRGVQSVDSLSSKRSSVCRLESKAPKVWSEPSDCVFFPVVVNKKVVQVHPLLLNDSEKRAVNHVLRNGNAGGKWLDSGDNDSLDNATNTRSGVSVDNSNASLDDDDDTLAANESKVTWKRGNYIAPQKQDDGRNNYSNVGSEQDSFILTRGWRRRTVTPEPRRNIVTRFAKLRSDSNSAADYTITWDKKKPYIGERSSDPGFELVGAPGHQSLVYRYNASTKDKRIDLNQLQNALTLKPNVPSGMSEDEFKKIQPSLRVVSGTDKKNGEGSKEVTVQLPDGKTRVDKFGNGSGFFTLNDEYINIPDLVLGNGNYGGGLNVSNTNGMNYSTLGNNYLNMSPQDITVNKWCEGGDTANPNCTSVNSESFNLEKVLGGHKNIETADDIKGTSKKAIAPVYALSLSNGWAMNSLKNTYGYNAQTHFKNVASAPTLLPVYVVPVDVIKPKAESIGSLKSSTMAKPYEVSANDIKFTFTGNPDTNGKVGGKELLVDASDDFDSRDVVEKNLQVCVRWMNNNKPQDKDCTPILKRDNSGNASVDSDKLQQMLVTHGNTAVYAVYAQTKDKSDNKSVGYDESKETAPIIGYIKITGINVSPIPLPFTGGNAAITYTFLFGILMALFIASGAFGRRGWLASVLSGNGFSGELTYSKHCNVSAEPLRCRRLFGLIYKNRH

**Schematic representation: SP-out-LPXTG-↓-in**

>lcl|AP012332.1_prot_BAQ33672.1_1020 [gene=GAVG_1020] [protein=putative cell surface protein]

**10 unique peptides were identified:**

TTNSAYTFISGR

IGQIGSDGLGDYDNTTK

AVDPSGNLNEVTVK

FGTSDDSGKVNSFSVK

TPGETEKAPTGTGTTTDPK

NAGDGVISNATTLTGVYK

GGNSNKQDLSDTFHK

ITATDNSGNVTTLTLTPK

NGTSPQTTNAVLSR

ELVVTDEAGNESK

MANSNRKKHAREQVESSFKNVDSSSAYATMRKVSLSTLATVLTAATCLGGQPILASSNILLPPTTAAFAQEVRNADPTPWLHTPNILEFQRYRFANSNGKIGQIGSDGLGDYDNTTKKSDMYAHLEKNGNDQYLIFDVFFNNDGKDMTGASKQQQYVWQIPFAVADLNNGYYNVDTLSDLSFNFYKRNGTSPQTTNAVLSRDFSLFTKENSQSATLSKPLQDDQHLGNSIYKITLGIRGGNSNKQDLSDTFHKNAGDGVISNATTLTGVYKGKSYGIGLRTTNVDYAVHMHCKVKLRQGVTLDDIQDAYTWANTSTYGRTTNSAYTFISGRERWKEPNLKRTKSDNLPPKLYFNGKEISDSNNSITVYQGDTPTIKFGTSDDSGKVNSFSVKGLPNGNGDVTENSNGMEATEAKHHNHVVNRVKFEYKNGNTPANTDYTVTVSATDASGNKVEKKVTVHLKNLNQKYKAPTTTPITVDWGHKATNEELKKHVSNIEGGGQLDFNTFPETKLPDIAFNGKDTATISTSAKVIYSDNSYHIVTIPVIVRKPLALQHKSELKDHEVKVNVGDTLTKTGNKVDAKQYLGITDKNVKDKIKSAEWVNGEPSTNVAGKKTYTAKVTFSDGSTAEEKVTFTVRPQKPKIETDLTGVAGVKGKEVVVNAGPGTAGSTVTLKDSKGNQIGTGKVGNDGKVTITVANGIPEGNITAVTTTADSVSSDASDAKKATKDDKAPTLTADKDSVTVKVGEDLKIQLTAKDDVKIESIDTATATMAFARNDFSKMMAVAARAKTDYTKNSDKEKVFTYNFSGFKPEEVGTYTLTFSATDAAGHKTEKTVTVKVEKKEDIKSKNIAVDLGHKLTEKDAEKAIANGRKLKKAGATFKWSTETGGTPDTSTVGKEKTGKVIVTIPTNDKNHPRVETVDVKVTVRDNESPKVAIQQKGDGGTYNTIDPGYPDGPKYPPVFTITTYRGDKNDIKITATDNSGNVTTLTLTPKTPGETEKAPTGTGTTTDPKTLTLDGTTPLNTKPGEYTRTLKAVDPSGNLNEVTVKFIVKPQSDKYGDVKGTPVTYNMGSGTKHPDPKSGINADDLKKLPSDATYSWAKEPDWNKPGEQKDAKVRITFKDGSTKDVTTTVTVKDDIAPVINTPSTNKAKRADTEKTAEYYQFKVHNGKPFSIDINAYDNSGKIKSATIATFPGHDGKITIDNQNGNSAEKPATIHIKGTAKRDASITNSSDNKWSRELVVTDEAGNESKLKVQISVYTDAEEHTATAKQVDNPKDNNAITNAITITKYDNPNTPIDKTGITFEVGKIPTDDGNYTVPVTVKYPDGSSSKIEVPIVVNRESNNYDPVGRTITVHWNSKEDIANKIKSFGDGNGLTWNGNSPKQNPTVEIVNTKDVPSTDKPGESKVQVKVKYSDGSSDVATVTVKVLDPQNKDYTPEGQTINIDYNKTPNTGISSKITGVGKDKGISFGKDKQGKDITLPEGASIAIDDKAKTVPDGTQPGTFDIPVTITYKDGSKDHATVKVTVGKPQSETHTPKVDGGITKKYGESGPSDNDIIAKVTVPNKPNGTTITVDKGQTPIDTKTPGTVNVKVTVKYPDGSEDHVEVPVTVGEPDNETYKPSAEAINKKRNEKAPDADEIKKAVKTSTVKNKTAPKETKVELVDPKTPLPTTDKTGTTNVPVKVTYPDGSSTVVQVPVVVTDTDATANTPAVDPVEEPYGTDKEKTKKAVTAAVTVPDFKPKKDGDKPTVTITSADKDIPDGTKPGTYHVKVKVTYPDNTTDEVTVDVTIKNKQSDTFEPKATPVVKPFGKKVTSDDVTGNVTVPNWSKDSKEQPKIELNEPNTKLPDGKTPGHSTVKVKVTYPDGTTDTVDVPVTISDKQSELYPPKADNLEKDNGQKPSDDEIIGKVSVPTWPTNETNKPTYSIAEGDKSKIPNGDKAGKFEVPVTVTYPDGSTTVINVPVTIKSPTATAKVVTVPKGVEPKPEDSIANKDKFPDGTKFEWDNNGKPDTSKEGNSVKGKVKITIPGSDTPQEVEVKVNVVDPSASEVNVPQGKDLPEAKDVIKDSHDTSKFPNGTTFEWKPEDKPDTNNPGAKNGKIIVTIPGQKPVEVSVTVNVLPKPEANVVNVPQNGTLPEAKDVIKDSSDTSKFHNGTTFEWEDSGKPTTDTVGEKTGGKVKVTIPGVNGKSGYTIDVPVTVNVNPVPIAKETTVPQNSNPDPKNSIDNNNKLPKGTTFEWKKDAAPKTDKPGTASGTVIVKIPGQAPQEVPVTITVTASPEGNDVTVLQKTDGKDNTPQPKDVIKNNGDLPENAKTSWKKKPDTSKTGNQPGIVTVKVPGEPDVDISVNVFVVPNPAGKTVSVHVGDSPSAENSIANKNELPNGTKFAWATNGTPDTKTAGNNKSGTVVVTIPGIANPVNVPVTVNVVAQNKPFINDGKAENKPGDKGSADNGKTTITGKGTPEATIKVQNSNGTALKDGNKEITVTVDKDGNFTVDVPHQTPGTTLKLVPNKGGVDGDATTVTVTAKPQKPTITVPTDNQKNDGNVTVTPPTDDTTVVKIEINAKPNSINGPEQPVRTIIAKKDNDGKWKIDGDAPDGVTVNPNTGVVTIPTKNLEDGSTITAVSKNKTDKPSDPATAVTGFKTPQISEQTLKDNPDDSTKQIITGKTLPGATVTVTDNNGNQIGTGTADKDGNFTITIDKQKPGAIVTLTPTNGKGDGAKTGDSVNITIGGNITKPNIDTPTNGGASVTPDLTDTRVNKVVVTYTPEGSETTATITVVAEGDKHEWKIDGDAPEGVTVNPQTGVVTIPANKIKSGSTITAQSEDSSDKTGKSKSEVVTAKVAEKEKPAPEPTPTPAGEPTILTPQDGVEQGSATVTPPADADTLEITYTPEGDTTTPVTITVKKDKNGNWTIDGNTPTGVTVDPKTGKVTIPATQIKDGSTITAQAKKNGQPSNMANGKVGNNPTKKPTPTPEPAPTPEPAPAPTPEHESGSHDSDEHTGNSENNRNKSNNTQLQAPKSPAHHSSGQVAQSKLTNTGSAIAAASTVAALAGAIGAALAVIIRRKKRD

**Schematic representation: SP-out-LPXTG-↓-in**

>lcl|AP012332.1_prot_BAQ32829.1_177 [gene=GAVG_0177] [protein=hypothetical protein]

**9 unique peptides were identified:**

AVFGSSEYTEDSLDSK

GLGFVANSIR

AMLQKAEDEQDAAESHR

AYNDLLDGK

TQLAQEAEQR

AEDEQDAAESHR

ITEAQYQAVK

TSSYQELK

KAVFGSSEYTEDSLDSK

MMNKKAIAAFAAGATLLAGFAMATPAMAAKVPATDTERTQLAQEAEQRGLGFVANSIRTSKTSSYQELKAMLQKAEDEQDAAESHRLLDYPNGTLNNSSAEGVAAEARKRGIANPTVDEFQPDLLAQYAGTRAEKLGKAYNDLLDGKITEAQYQAVKKAVFGSSEYTEDSLDSKQDDKQAPKKDDKKDDTTTPDQKIKDLGKLNLSDAAKKKLAVHYVYKAKLALDEADNNLAEKKADYAAKRKTLVETMTELAARKAAAEKANQDLTDFLASGENNSAKETALRDALNRAHAHEQRAFDAEQKAEAEFNAARDAAFAAVAAYNKALAEYKDAYNDAVRLGVNPAALPPVVTSDPLAADFPAVPGTKQIYADALNGKFGPAAQASAKKTEAAKAQATAPAAAGKAAAKGELAAKGAKGHGKAGEKLGNAGVGVALTALAASMLAGMGAAVRKMRH

**Schematic representation: SP-out-↓-in**
